# Systematic review on fiscal policy interventions in nutrition

**DOI:** 10.3389/fnut.2022.967494

**Published:** 2022-11-29

**Authors:** Jane Hammaker, Daniela Anda, Tomasz Kozakiewicz, Vinitha Bachina, Miriam Berretta, Shannon Shisler, Charlotte Lane

**Affiliations:** International Initiative for Impact Evaluation (3ie), Washington, DC, United States

**Keywords:** fiscal policies, nutrition, sugar-sweetened beverage consumption, taxes, subsidies

## Abstract

**Introduction:**

Both the World Health Organization and the Lancet Series on Adolescent nutrition recommend that governments adopt fiscal policies to combat diet-related non-communicable diseases (NCDs). However, rigorous, systematic evidence regarding the effects of these interventions is lacking.

**Methods:**

We synthesize the available evidence regarding the impacts of taxes and subsidies that directly affect consumer prices on availability and accessibility of foods and beverages, purchasing behavior, diet quality, health and well-being outcomes as well as considerations for implementation, sustainability and equity.

**Results:**

Our initial search returned 2,113 de-duplicated studies, and ultimately 24 impact evaluations and two systematic reviews met final eligibility criteria and represented unique evaluations. Our meta-analysis of these studies suggests that taxes may decrease purchases of taxed beverages (SMD = −0.14 [95% CI: −0.29 to −0.07], *n* = 15). Results should be interpreted cautiously due to considerable heterogeneity (*Q*(14) = 335.19, *p* = 0.01, ${\hat{\tau }}^{2}=0.03$, *I*^2^ = 95.82%).

**Discussion:**

The evidence base is too limited to draw conclusions about the effects of taxes on beverages and calorie-dense foods on purchases, or on the effects of subsidies on purchasing or diet quality. Overall, the evidence base is inconclusive on whether fiscal policies can meaningfully influence the availability and accessibility of foods and beverages, diet quality, and health outcomes. Policymakers implementing fiscal policies should consider information campaigns on health benefits and health risks associated with certain food and beverage consumption. For taxes, exposure to health information may amplify signaling effects of taxes and reduce avoidance behaviors, such as cross-border shopping. Future evaluations should diversify data sources to better understand impacts on diet and health outcomes.

## Introduction

Malnutrition in all its forms, including undernutrition (wasting, stunting, and underweight), overweight and obesity, affects at least 2.6 billion people worldwide (1). In 2021, non-communicable diseases (NCDs) accounted for over 70 percent of deaths globally, led by cardiovascular disease (17.9 million people), cancers (9.3 million), respiratory diseases (4.1 million), and diabetes (1.5 million) (2). Both the WHO and the Lancet Series on Adolescent nutrition recommend that governments adopt fiscal policies, such as taxes and subsidies, to combat diet-attributed NCD risk. The goal of such policies is to either discourage the consumption of calorie-dense beverages and foods or encourage diverse diets that include fruit, vegetables, legumes, nuts and whole grains (3–5). Taxes on non-alcoholic sugar-sweetened beverages (SSBs) have been implemented in over 50 countries, generally in the form of per-unit excise taxes (e.g., a juice in Mexico is taxed at one peso per liter) or ad-valorum excise taxes [e.g., an energy drink in Saudi Arabia is taxed at 50 percent of pre-tax price; (6)]. The effects of these taxes on changes in price, including pass-through rates from distributors to consumers, is well documented. However, the effects of taxes on consumption, diet, and health outcomes remains unclear (7). Subsidies are implemented in nearly every country in the world, and previous reviews have synthesized the effects of monetary subsidies on food purchases and consumption in field experiments (8) and in modeling studies (9). To our knowledge, this is the first attempt to synthesize the empirical evidence base on the impacts of subsidies in which the government pays a *portion* of the price of a good on diet and health outcomes.^1^ Since they affect people globally, we need to know if taxes and subsidies meaningfully improve diet, health, and well-being.

To address this gap and support evidence-informed decision-making, we conducted a systematic review of the effects of fiscal policies linked to food and beverages on the availability of and access to diverse diets. This systematic review challenges and verifies the hypothesis of the international community that these interventions improve diet, health, and well-being. Researchers and implementers can use this work to better understand how to structure and implement taxes or subsidies to facilitate behavioral change among consumers and industry.

## Methodology

This systematic review is based on topically relevant studies identified by the Food Systems and Nutrition Evidence Gap Map and a systematic literature search of key academic databases (10). We assessed literature for quality and summarized it visually and in a narrative format. The review followed the rigorous Campbell Collaboration and Cochrane approaches to systematic reviewing (11, 12).

### Expected theory of change

**FIGURE 1:**
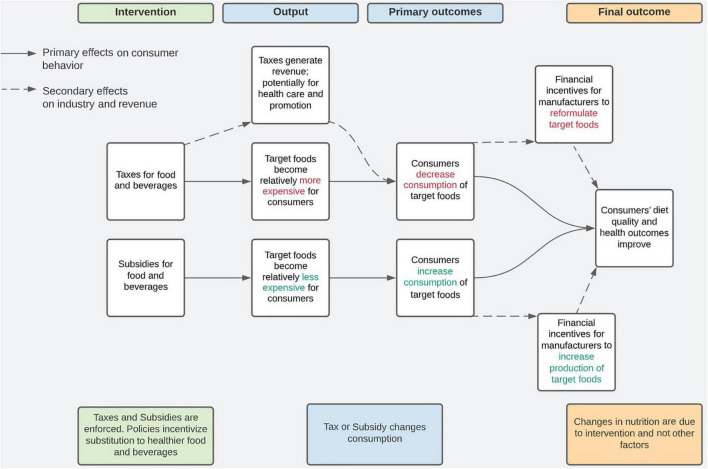
Fiscal policy theory of change, World Health Organization (4).

The theory of change is that policymakers implement taxes and subsidies on foods and beverages to influence the availability and accessibility of foods and beverages. When the price of taxed goods increases, we expect consumers to change their purchasing behavior by decreasing their consumption of taxed foods. When the price of subsidized goods decreases, we expect that consumers will change their purchasing behavior by increasing their consumption of subsidized foods. Taxes generate revenue for the implementor, which, if invested in health or nutrition initiatives, may contribute to changes in consumption in the population. Changes in consumption may generate financial incentives for manufacturers to reformulate or modify production of target foods. Changes in diet attributable to fiscal policies will promote consumers’ diet quality, anthropometrics, health and well-being outcomes (Figure 1).

### Objectives and research questions

The objective of this work is to synthesize the available evidence regarding the impacts of tax and subsidy interventions on availability and accessibility of foods and beverages, purchasing behavior, diet quality, anthropometrics, health and well-being outcomes. We also identify considerations for implementation, sustainability and equity. We specified the following research questions (RQ) *a priori:*

RQ1: What are the effects of fiscal policies on food and beverages on the availability and access to diverse diets?

RQ2: Are there unintended consequences of these actions, such as food substitutions or regressive effects?

RQ3: What policy design features moderate impact? For example, do effects vary by the specific approach taken, food targeted, socio-economic status, or context, including the joint implementation of fiscal policies with other initiatives?

RQ4: What evaluation design strategies are used? What relationships and data sources are key to allowing for evaluation?

RQ5: What are common implementation challenges, sustainability issues, and implications for practitioners in both high-income countries (HICs) and low- and middle-income countries (L&MICs)?

We conduct meta-analysis to synthesize effects (RQ1) and unintended consequences (RQ2) related to substitutions. We conducted qualitative thematic analysis to further probe unintended consequences (RQ2), consider policy design features (RQ3), identify study design strategies (RQ4), and develop implications for implementation, sustainability and equity (RQ5).

### Criteria for including and excluding studies in the review

#### Types of study participants

There were no limitations on study participants’ country of origin, gender, ethnicity, age, or other demographic trait (Table 1).

**TABLE 1:** Description of participations, intervention, comparison, outcomes, study designs.

Criteria	Included	Excluded
Participants	Individuals in L&MICs and HICs	Niche populations, such as astronauts, people in the military, professional athletes, etc.

Intervention	Taxes for of calorie-dense foods and beverages that are high in sugar, fat or salt (e.g., sugar-sweetened)	In-kind food provision (e.g., free school meal handouts) or fully subsidized products
		Food vouchers or cash transfers/cashbacks/food stamps even if they are referred to as subsidies [e.g., the Supplemental Nutritional Assistance Program (SNAP) in the United States]. These interventions do not explicitly decrease the market price of targeted foods and beverages
		Fiscal policies that are not subsidies or taxes, such as price ceilings
		Reduction on import taxes for vegetables and fruits.
	
	Consumer subsidies for nutritious foods (e.g., fruits, vegetables, legumes, pulses, and fortified wheat) and beverages (e.g., fortified milk for pregnant mothers)	General consumption behavior change interventions
		Agricultural input subsidies
		Consumer subsidies not directly aimed at supporting a diverse, nutritious diet (e.g., for corn, rice, wheat, salt, wine grapes unless fortified with micronutrients)
		Price changes, which would not be considered an intervention by itself
		Lab in the field or field experiments such as virtual supermarkets

Comparison	Business as usual, including pipeline and waitlist controls	If there is no counterfactual
	An alternate intervention	

Outcome(s)	Availability and accessibility of targeted foods and beverages	Affordability of diverse, nutritious foods and beverages, including post-tax or post-subsidy changes in price
	Purchasing behavior/patterns	
	
	Diet quality and adequacy	Outcomes unrelated to nutrition, such as tax revenue, public finance
	Anthropometrics	
	Health, including diseases associated with nutrition (e.g., diabetes or heart disease)	
	Well-being (e.g., psychological measures and the acceptability of diet)	

Study designs	Experimental and quasi-experimental impact evaluations	Qualitative impact evaluations
	Systematic reviews of experimental and quasi-experimental impact evaluations	Descriptive or observational studies that do not assess effectiveness
	Ex-post cost evidence	Modeling studies*
		Ex-ante cost evidence

*We excluded studies that use observed data to predict outcomes for both treatment and control groups. However, in some cases, these models are employed for evaluating taxes and subsidies due to the nature of implementation and data availability.

#### Types of interventions

We considered two types of fiscal policies, implemented by governments, for this systematic review:

1. Taxes that increase prices of high-sugar foods and non-alcoholic beverages, such as SSBs, to discourage consumption.
2. Subsidies to decrease prices of targeted foods and beverages, such as fruits, vegetables and pulses, to encourage consumption and diversify nutrient uptake (5).

We considered studies of taxes that directly increase the consumer price of calorie-dense foods and beverages that are high in sugar, fat or salt through the government charging an additional fee to manufacturers, stores, or the consumers. Taxes on producers, processors, or other downstream actors in the value chain were not considered (Table 1).

We define subsidies as interventions in which the government pays a *portion* of the price of a good. Studies must explicitly mention that they evaluated subsidies. Often, authors refer to interventions which provide food, cash, or vouchers as subsidies. While these programs can all be conceptualized as reducing costs and increasing accessibility of food, the behavioral responses of consumers to the various delivery mechanisms are likely to be different. Therefore, we did not include these interventions. Subsidies can be implemented alongside other governmental programs, such as subsidizing a portion of the cost of a school meal which students are expected to pay. We considered subsidies that targeted foods such as fruits, vegetables, pulses, legumes, and fortified grains. We excluded unfortified staple crops, wine grapes, and salt (Table 1).

#### Types of outcome measures

We considered outcomes related to availability and accessibility of foods and beverages, such as food assets and production; purchasing behavior, such as sale of foods or frequency of purchases; diet quality and adequacy, such as composite diet scores or dietary diversity; anthropometrics, such as body mass index; health, such as incidence of non-communicable diseases; and well-being, such as measures of anxiety related to food insecurity. *A priori*, we specified preferred outcomes and alternate outcomes for synthesis (Table 2). We preferred composite measures over disaggregated ones. Additional information on indicators that we considered for each outcome are specified in the protocol (Supplementary material 1).

**TABLE 2:** Included outcomes and indicators extracted for evidence synthesis.

Outcome	Indicators*
Availability and accessibility of foods and beverages	Preferred outcomes: food assets, production (community gardens), and stores
	Other measures: distance and accessibility to markets, were considered if these are not available
Purchasing behavior	Preferred outcome: sales of food in monetary units
	Secondary outcome: frequency or change of purchase of foods
Diet quality and adequacy	Preferred outcomes: composite diet scores such as the nutrient rich food index
	Secondary outcome: dietary diversity and other food variety measures
	Tertiary outcome: intake of specific foods
Anthropometrics	Preferred outcomes: body mass index, weight for length, length for age, and weight for age
	Other measures, such as mid-upper arm circumference (MUAC) and ponderal index, were considered if these are not available
Health	Incidence of diseases *directly* tied to nutrition, especially with regard to nutrition-related non-communicable diseases (NCDs), including diabetes, anemia, metabolic syndrome, and cardiovascular disease. Indirect diseases, such as cancer, will not be considered.
Well-being	Preferred outcome: perceived well-being
	Secondary outcome: anxiety, often regarding food security

*Indicators are listed by preference based on *a priori* specification. Such *a priori* specification reduces bias by preventing subjective reporting of outcomes by the team conducting the systematic review. Most indicators were ultimately not found in the studies.

#### Types of comparators

We considered alternate intervention or business as usual comparators, including pipeline and waitlist controls, as valid comparators. Studies with no valid counterfactual were excluded.

#### Types of study design

We considered experimental and quasi-experimental studies for inclusion in the meta-analysis, including:

• Randomized controlled trial
• Regression discontinuity design
• Controlled before-and-after studies, including
  ⚬ Propensity-weighted multiple regression
  ⚬ Instrumental variable
  ⚬ Fixed-effects models
  ⚬ Difference-in-differences (and any mathematical equivalents)
  ⚬ Matching techniques
• Interrupted time series

We also included ex-post cost-effectiveness analyses and systematic reviews that include a quantitative or narrative synthesis.

#### Date, language, and form of publication

We included studies published after 2000 and written in English.

### Search strategy

An information specialist developed the search string with subject-matter input by the research team. The team verified the sensitivity of the search strings by ensuring that search results included the eligible studies from the Food Systems and Nutrition Evidence Gap Map. Ultimately, one of these studies was not identified through the final search because it was published in a relatively less well-known journal that is not indexed in major databases, the Latin American Economic Review journal. Due to resource constraints, we limited the number of databases searched. Search terms are provided in Supplementary material 1.

#### Electronic searches of bibliographic databases and library catalogs

The information specialist searched the following twelve databases:

• CAB Abstract (EBSCO)
• Agricola (EBSCO)
• Medline (EBSCO)
• Academic Search Complete (EBSCO)
• PsycInfo (EBSCO)
• Africa-Wide (EBSCO)
• CINAHL (EBSCO)
• Scopus
• Embase (Ovid)
• CAB Global Health (Ovid)
• Cochrane Library (this contains 2 databases—Trials Register and the SR database).

#### Other searches

In addition to the search of academic databases, the team searched for additional, relevant studies that had been previously identified from the search by Moore et al. (10) and its recent update. These studies may have been excluded because they considered participants from high-income countries or used ineligible study designs. The following studies were added to the search:

• Studies from the original map excluded using the code ‘High income country’ on title and abstract or full text, with the term ‘tax*’ or ‘subsid*’ on title or abstract.
• Studies from the original map included on title and abstract that have the term ‘tax’ or ‘subsid*’ in title or abstract.
• Studies from the EGM update (as of 21/01/2022) with the code ‘Exclude- High income country’ on title and abstract or full text that have the term ‘subsid*’ or ‘tax*’ on title or abstract.
• Studies from the EGM update (as of 19/01/2022) with the code ‘FSN marker TA screening—FSN relevant’ with subsid* or tax* on title or abstract.

### Selection of studies

#### Screening

For title and abstract screening, the team developed a machine learning classifier in EPPI Reviewer. Two research associates screened studies with a prioritization score of 0.3 (30 percent likelihood of inclusion) or higher independently at title and abstract. One research assistant screened records with a prioritization score of 0.2–0.29. Research associates did not screen records with a probability of inclusion below 20 percent. Two research associates then screened all records included at the title and abstract stage at full text. The research team subsequently trained research assistants on the screening protocols and instructed them to apply exclusion codes in a hierarchical order for consistency in coding. Research assistants discussed differences in inclusion decisions, consulting with the research lead if disagreements could not be reconciled.

#### Data extraction and coding procedures

Once included impact evaluations and systematic reviews were identified, the team conducted an initial round of data extraction to determine the methods, interventions, and outcomes used. Because many studies considered the same intervention and outcomes within the same population, they did not represent unique evaluations; so, they could not all be included in the final analysis. We used the following, hierarchical criteria to select a single study for each intervention-outcome-population combination for inclusion in the meta-analysis: (1) the most biologically relevant outcomes, (2) the most rigorous analytical method, or (3) the longest time frame (Supplementary Appendix Tables 1, 2). The ranking criteria were only employed when selecting among outcomes that fell within the same category outlined in Table 2. Effect estimates from systematic reviews were not considered in meta-analysis as all the policies considered within the systematic reviews were already reflected in the studies included in this review.

Using 3ie’s repository coding protocols, we modified data extraction templates typically used for systematic reviews (Supplementary material 2). For analyzed (Supplementary Appendix Table 1) and linked studies (Supplementary Appendix Table 2), we extracted bibliographic and geographic information, equity considerations, standardized methods, project-specific interventions and outcomes, population of interest (disaggregated by gender and age, where possible), barriers and facilitators to implementation, sustainability, cost, and other considerations for practitioners. For analyzed studies, we also extracted effect sizes relevant to the theory of change (Figure 1). If a single study reported several different analyses on the same outcome (e.g., presented an adjusted and unadjusted model), we selected the model preferred by the authors for extraction. If the authors did not clearly state a preferred model, we extracted data from the model with the most control variables. Two independent reviewers completed data extraction, except in the case of two systematic reviews and linked publications which were extracted by one person each.

#### Critical appraisal

Two independent reviewers appraised all analyzed quantitative impact evaluations and systematic reviews of impact evaluations using a critical appraisal tool based on their study design (Supplementary material 3).

### Analytical approach for quantitative and qualitative data

To reply to RQ1, we selected studies with sufficient data for meta-analysis. We chose the appropriate formulae for effect size calculations in reference to, and dependent upon, the data provided in included studies. We conducted random effects meta-analyses when we identified two or more studies that measured similar underlying concepts, such as purchasing behavior or consumption. We assessed heterogeneity by calculating the Q statistic, I^2^, and τ^2^ to provide an estimate of the amount of variability in the distribution of the true effect sizes (13). We explored heterogeneity using moderator analyses if the data allowed. Moderators considered included taxes vs. subsidies, food targeted and socioeconomic status (SES). There were not enough studies in L&MICs to conduct moderator analysis by country income level. We also tested for the presence of publication bias if at least ten studies were included in the analysis.

There was insufficient data to answer the remaining research questions quantitatively, so we conducted qualitative, thematic analysis on quantitatively analyzed and linked studies. While reviewing data extracted from each study, one coder identified common topics, ideas and conclusions across studies. She created themes around these common ideas and grouped extracted information accordingly. For example, if an author mentioned ‘tax avoidance’ as a barrier, the coder created a theme ‘Barriers—Tax avoidance’ and subsequently grouped similar information from other studies under this theme. Once the qualitative information was organized by theme, the coder prioritized findings by frequency and relevance to quantitative findings. Five reviewers from the research team validated findings from the thematic analysis.

### Data presentation

We provide a narrative summary of the papers identified. This includes an overall description of the available literature and a general synthesis of findings. We summarize key information from each study, including intervention type, study design, country, outcomes, measurement type, effect sizes and confidence rating. Then, we present results from meta-analyses and their associated forest plots in the findings section. We also present qualitative information in a section on theories of change, unintended effects and implementation considerations to provide actionable insights for policy design.

## Results

### Search results and characteristics of the included studies

**FIGURE 2:**
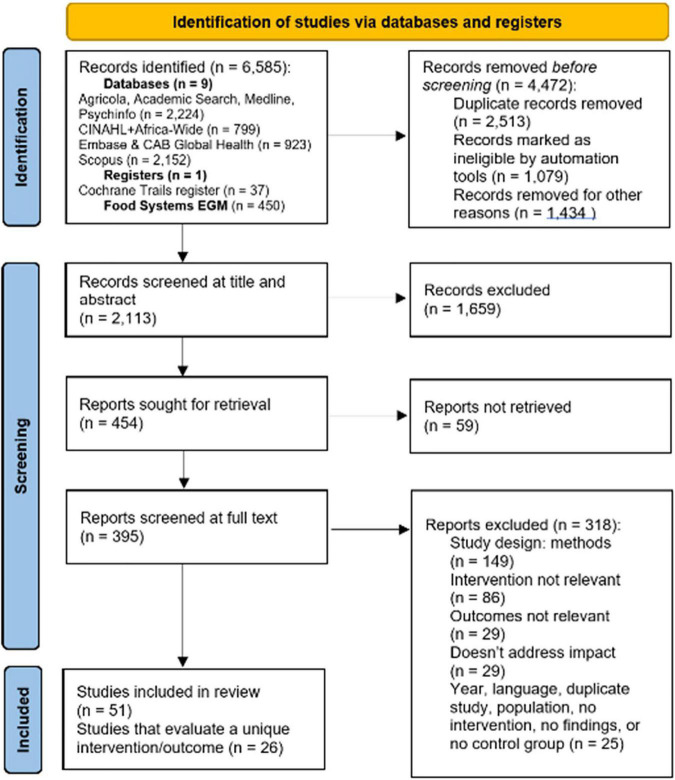
PRISMA diagram, 3ie (2022).

Our initial search returned 6,585 studies, of which 2,113 remained after de-duplication (Figure 2). After title and abstract screening and full text retrieval, 422 impact evaluations and 32 systematic reviews remained for full text review. Ultimately, 49 impact evaluations and two systematic reviews met eligibility criteria (*n* = 51). Half of these did not represent unique evaluations as they considered the same tax and outcome. For example, three studies evaluated the impact of a SSB tax on purchases of taxed beverages in Berkley, CA, United States. Therefore, 24 impact evaluations and two systematic reviews met eligibility criteria, considered unique intervention-outcome-population combinations and were included in the quantitative analyses. Studies included the meta-analysis are presented in Supplementary Appendix Table 1, and linked studies are listed with qualitative information in Supplementary Appendix Table 2. Qualitative information from analyzed studies are presented in Supplementary Appendix Table 3.

#### Impact evaluations

**FIGURE 3:**
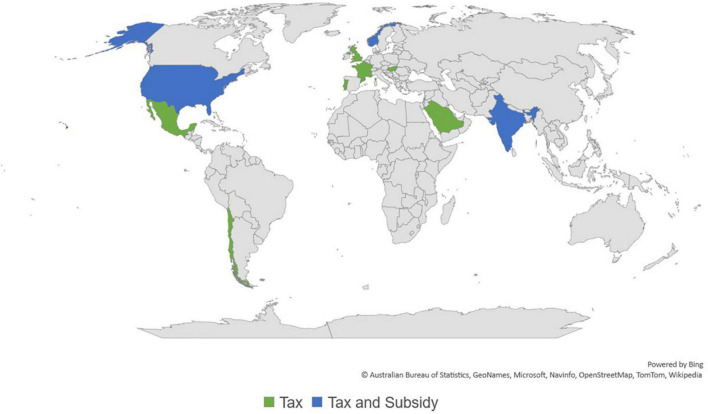
Map of analyzed studies, 3ie (2022).

Most of the 24 impact evaluations were in HICs, primarily in the United States (*n* = 8) and Europe (*n* = 8) (Figure 3). Six studies took place in L&MICs contexts, which included Mexico (*n* = 3) and India (*n* = 3).

We included 20 studies that evaluated taxes in the main analysis. Most of the tax studies evaluated taxes targeting SSBs alone (*n* = 13), but four taxes targeted SSBs and high-sugar foods, and two targeted carbonated or aerated beverages. Eleven countries, states or cities implemented excise taxes on beverages using a per-unit tax, such as the Public Health Product Tax in Hungary that taxes soft drinks at HUF 200 per liter (14) or the Oakland Sugar-Sweetened Beverage Distribution Tax in CA, United States that taxes SSBs at USD 0.01 per ounce (15). Four countries implemented ad-valorum excise taxes on beverages or foods, such as the Excise Tax Implementing Regulations that included a 50 percent excise tax on SSBs in the Kingdom of Saudi Arabia (16) or Mexico’s 8 percent tax on solid foods with high caloric density (17). Two studies evaluated ‘tiered-taxes’ implemented in Catalonia and Portugal that had higher tax rates for high- and low-sugar beverages. While most taxes targeted any SSB, including soda and juice (*n* = 18), two policies exclusively taxed carbonated beverages, two policies imposed additional taxes on caffeine content, and one policy taxed artificially sweetened beverages. Most of the tax studies measured the impact of taxes on purchases of taxed or untaxed beverages.^2^ A few also considered outcomes related to purchases of high-sugar foods (*n* = 4) or diet quality (*n* = 2). Two studies exclusively measured the impact of taxes on diet quality or health without focusing on purchasing outcomes.

Four included studies evaluated subsidies: two targeted fruits and vegetables, one targeted fortified wheat and one targeted pulses. All four studies measured the impact of subsidies on diet quality or health outcomes (Table 3).

**TABLE 3:** Summary of included studies.

Intervention group	Number of studies	Implementation countr(ies)	Outcomes evaluated	Indicators evaluated
Taxes on SSBs, ASBs or carbonated beverages (14–16, 20, 21, 24, 26, 29–31, 34–40, 42)	*N* = 18	Barbados. United States, Spain, Chile, France, Portugal, Saudi Arabia, United Kingdom	All purchases	Calories purchased in beverages; calories purchased in high-sugar foods; sugar purchased in beverages; sugar purchased in high-sugar foods; volume of purchased beverages
			Taxed purchases	Calories purchased in taxed beverages; volume of beverage purchases
			Untaxed purchases	Calories purchased in untaxed beverages; grams of sugar purchased in beverages; volume of purchased beverages
			Diet quality	Consumption of grams of added sugar; ratio of post to pre-tax prevalence of regular consumption of taxed beverage
Taxes on SSBs and high-sugar foods (17, 32, 33, 41)	*N* = 4	Mexico, Hungary, Norway	All purchases	Calories purchased in beverages; volume of purchased beverages
			Taxed purchases	Calories purchased in taxed beverages; volume of beverage purchases
			Untaxed purchases	Calories purchased in untaxed beverages; volume of purchased beverages
			Health	Outpatient visits for dental carries
Subsidies for staples (pulses, fortified wheat) (22, 25)	*N* = 2	India	Subsidized purchases	Purchases of pulses
			Diet quality	Daily household intake of protein
			Health	hemoglobin levels
Subsidies for fruits and vegetables (18, 28)	*N* = 2	Norway, United States	Diet quality	Intake of fruits (excluding fruit juices) and vegetables (excluding potatoes); servings of fruit in previous week

The evidence was overwhelmingly quasi-experimental (*n* = 23), and one impact evaluation used a randomized design. Quasi-experimental studies used difference-in-differences (*n* = 10), interrupted time series (*n* = 9), synthetic control (*n* = 2), instrumental variables (*n* = 1) and both synthetic control and difference-in-differences for two interventions evaluated separately (*n* = 1). Nearly all (*n* = 17) quasi-experimental studies relied on consumer purchase data from global bases such as Kantar World Data, Nielsen and Euromonitor.

#### Systematic reviews

After the initial search and title and abstract screening, 32 systematic reviews were screened at full text. Common exclusion reasons for systematic reviews were evaluation methods (many of the included studies within these SRs did not meet the eligibility criteria), and interventions not relevant to the scope of this review. Ultimately, two systematic reviews met the inclusion criteria. One review included one study from Hungary, and the other conducted a quantitative meta-analysis of evidence from the United States, Chile, France, Mexico, and Spain. Both included reviews synthesized the impacts of taxation. One searched for taxes on sugar and sugar added foods but found only one evaluation of a tax on foods high in sugar, salt and caffeine, including SSBs. The other study reviewed the effectiveness of taxes on SSBs.

#### Risk of bias in impact evaluations and systematic reviews

Overall, the quality of the impact evaluations is low; we assessed all evaluations to have some concerns or high risk of bias for at least two criteria (Table 4). Common quality concerns were related to confounding and reporting bias. Both systematic reviews were assessed with high confidence (Table 5). There were minimal concerns with the causal chain used in the review to analyze studies and the type of evidence incorporated to inform the analysis and reporting.

**TABLE 4:** Risk of bias of analyzed impact evaluations.

Study name	Review criteria: Randomized control trial, difference-in-difference, and instrumental variable designs								
	Assignment mechanism	Unit of analysis	Selection bias	Confounding	Deviations from intended interventions	Performance bias	Outcome measurement bias	Reporting bias	Overall risk of bias:
Øvrum and Bere (18)	3	3	3	3	8	2	8	3	High
Alsukait et al. (16)	N/a	N/a	3	4	2	1	1	8	High
Cawley et al. (15)	N/a	N/a	1	2	2	8	8	1	Some concerns
Cawley et al. (19)	N/a	N/a	1	3	2	1	1	1	High
Gonçalves and Pereira dos Santos (20)	N/a	N/a	2	3	2	1	1	1	High
Rojas and Wang (21)	N/a	N/a	8	3	8	1	1	1	High
Chakrabarti et al. (22)	N/a	N/a	8	4	8	1	1	1	High
Bleich et al. (23)	N/a	N/a	3	8	8	2	1	1	High
Royo-Bordonada et al. (24)	N/a	N/a	2	3	8	2	2	4	High
Chakrabarti et al. (25)	N/a	N/a	2	3	3	1	1	1	High
Powell et al. (26)	N/a	N/a	2	4	8	1	1	3	High
Colchero et al. (27)	N/a	N/a	8	3	3	1	2	2	High
Howard and Prakash (28)	N/a	N/a	8	8	1	2	2	8	Some concerns

**Review criteria: Interrupted time series and fixed effect designs**									

	**Assignment mechanism**	**Unit of analysis**	**Selection bias**	**Confounding**	**Deviations from intended interventions**	**Bias due to missing outcome data**	**Outcome measurement bias**	**Reporting bias**	**Overall risk of bias:**

Alvarado et al. (29)	N/a	4	N/a	0	1	0	0	1	Some concerns
Silver et al. (30)	N/a	1	N/a	1	2	0	0	2	High
Law et al. (31)	N/a	4	N/a	1	1	0	0	0	Some concerns
Hernández-F et al. (32)	N/a	4	N/a	1	0	0	0	1	Some concerns
Øvrebø et al. (33)	N/a	4	N/a	1	0	0	0	1	Some concerns
Pell et al. (34)	N/a	4	N/a	1	0	1	0	1	Some concerns
Aguilar Esteva et al. (17)	N/a	4	N/a	1	0	0	0	1	Some concerns
Powell and Leider (35)	N/a	4	N/a	1	1	0	0	2	High
Nakamura et al. (36)	N/a	4	N/a	1	1	0	0	1	Some concerns

**TABLE 5:** Risk of bias of systematic reviews.

Review criteria	Teng et al. (37)	Pfinder et al. (38)*
Methods used to identify, include and critically appraise studies	High confidence	High confidence
Methods used to analyze the findings relative to the primary question addressed in the review	High confidence	High confidence
Overall reliability of the review	High confidence	High confidence

*Pfinder et al. (38) only includes one evaluation on the Hungary tax, which was evaluated in one analyzed study (14).

### Effects of fiscal policies

We present the meta-analysis results for taxes and subsidies separately in Tables 6, 7, with additional results in Supplementary material 4. Results which consider only one or two studies should be interpreted with caution.

**TABLE 6:** Results from meta-analysis considering the effects of taxes and subsidies.

Outcomes	# of included effects (total number of beneficiaries)	Overall effect size [95% CI]	Estimated percentile change compared to control group [95% CI]	Heterogeneity of overall effect: Q, *I*^2^	Range of effects
**Impacts of taxes on purchases of any beverage**					
Total beverage purchases	5 (9,812)	−0.07 [−0.25; 0.11]	−2.8% [−9.9%; 4.4%]	22.17,** 81.96%	−0.21 to 0.40
Taxed beverage purchases	15 (86,971)	−0.18* [−0.29, −0.07]	−7.1% [−11.4%; 2.8%]	335.19,** 95.82%	−2.51 to 0.91
High-tax beverage purchases	2 (20,835)	−0.11 [−0.30; 0.09]	−4.4% [−11.8%; 3.6%]	23.51,** 95.7%	−0.21 to −0.01
Low-tax beverage purchases	2 (33,598)	−0.02 [−0.05; 0.02]	−0.8% [−2%; 0.08%]	1.35, 25.8%	−0.03 to 0.02
Untaxed beverage purchases	11 (34,977)	−0.02 [−0.06; 0.02]	−0.8% [−2.4%; 0.8%]	19.61,* 49.01%	−0.09 to 0.62
Untaxed beverage purchases among high SES	2 (3,474)	−0.08 [−0.25; 0.09]	−3.2% [−9.9%; 3.6%]	5.78,* 82.71%	−0.17 to 0.00
Untaxed beverage purchases among middle SES	2 (4,848)	−0.03 [−0.17; 0.11]	−1.2% [−6.7%; 4.4%]	3.91,* 74.40%	−0.09 to 0.05
Untaxed beverage purchases among low SES	2 (2,495)	−0.29 [−0.72; 0.14]	−11.4% [−26.4%; 5.6%]	29.30,** 96.59%	−0.51 to −0.07
Taxed, high-sugar food purchases	2 (10,819)	−0.02 [−0.07; 0.02]	−0.8% [−2.8%; 0.8%]	1.57, 36.13%	−0.04 to 0.01
Untaxed, high-sugar food purchases	2 (4,423)	0.12 [−0.08; 0.32]	4.8% [−3.2%; 12.6%]	11.06,** 90.96%	0.02 to 0.22
**Impacts of taxes on diet**					
Diet quality	2 (2,270)	0.19 [−0.34; 0.72]	7.5% [−13.3%; 26.4%]	21.58,** 95.4%	−0.09 to 0.45
**Impacts of subsidies**					
Purchases of pulses	4 (450,998)	0.02 [0.01; 0.03]	0.8% [0.4%; 1.2%]	3.04, 1.6%	0.01 to 0.02
Diet quality	3 (119,039)	0.06 [−0.01; 0.14]	2.4% [−0.4; 5.6%]	12.79,* 84.37%	0.01 to 0.22
Hemoglobin levels	2 (4,676)	−0.005 [−0.06; 0.05]	0.0% [−2.4%; 2%]	0.05, 0.0%	−0.01 to −0

*p ≤ 0.05; **p ≤ 0.01.

**TABLE 7:** Effect estimates from included studies.

First author	Year	Region(s)	Evaluation or synthesis method	Outcome	Standardized effect estimate (confidence interval)	Independent units	Number of repeated measures
**Purchases of any beverage**							
Nakamura et al. (36)	2018	Chile	Fixed effects	Grams sugar sales from all soft drinks*	−0.21 [−0.29; −0.14]	2,836 households	1 observation per household
Nakamura et al. (36)	2018	Chile	Fixed effects	Gram sugar sales from all soft drinks (high SES)	−0.27 [−0.39; −0.16]	1,138 households	60 observations per household
Nakamura et al. (36)	2018	Chile	Fixed effects	Gram sugar sales from all soft drinks (medium SES)	−0.18 [−0.31; −0.06]	963 households	60 observations per household
Nakamura et al. (36)	2018	Chile	Fixed effects	Gram sugar sales from all soft drinks (low SES)	−0.11 [−0.23; 0.01]	1,120 households	60 observations per household
Aguilar Esteva et al. (17)	2019	Mexico	Regression discontinuity design	Total calories contained in all purchased products*	−0.01 [−0.06; 0.04]	6,935 households	104 observations per household
Silver et al. (30)	2017	Berkley	Interrupted time series	% change in volume of taxed or untaxed beverages sold per transaction relative to counterfactual developed based on pre-intervention trends*	0.14 [−1.02; 1.3]	9 stores	1,128 observations per store
Kruz et al. (14)	2020	Hungary	Synthetic control	SSB sales in milliliters*	0.20 [−0.73; 1.13]	16 stores	15 observations per store
Kruz et al. (14)	2020	France	Synthetic control	SSB sales in milliliters*	0.40 [−0.53; 1.33]	16 stores	15 observations per store
Powell et al. (26)	2021	Seattle	Difference-in-difference	Ratios of incidence rate ratios (RIRRs) showing the percentage change in grams of sugar sold in Seattle compared with Portland—from standalone sugar	0.12 [−0.31; 0.55]	81 brands	2 observations per brand
**Purchasing of taxed beverages**							
Alsukait et al. (16)	2020	Saudi Arabia	Difference-in-difference	Carbonated drinks’ annual volume sales (liters per capita)*	−2.51 [−4.06; −0.95]	7 years	1 observation per year
Powell et al. (26)	2021	Seattle	Difference-in-difference	Ratios of incidence rate ratios (RIRRs) showing the percentage change in grams of sugar sold in Seattle compared with Portland—from taxed beverages*	−1.01 [−1.13; −0.90]	1326 brands	2 observations per brand
Powell and Leider (35)	2020	Cook County	Interrupted time series	Taxed beverage volume sold in Cook County, Illinois, relative to St Louis County and City, Missouri—change in level after tax	−1.30 [−1.65; −0.96]	138 weeks	1 observation per week
Powell and Leider (35)	2020	Cook County	Interrupted time series	Taxed beverage volume sold in Cook County, Illinois, relative to St Louis County and City, Missouri—change in slope after tax	−0.08 [−0.41; 0.25]	138 weeks	1 observation per week
Powell and Leider (35)	2020	Cook County	Interrupted time series	Taxed beverage volume sold in Cook County, Illinois, relative to St Louis County and City, Missouri—change in level after tax repeal	1.09 [0.53; 1.65]	51 weeks	1 observation per week
Powell and Leider (35)	2020	Cook County	Interrupted time series	Taxed beverage volume sold in Cook County, Illinois, relative to St Louis County and City, Missouri—change in slope after tax repeal	0.22 [−0.32; 0.77]	51 weeks	1 observation per week
Puig-Codina et al. (42)	2020	Catalonia	Synthetic control	Liters cola purchased per person per month*	−0.67 [−1.59; 0.24]	17 regions	78 observations per region
Alvarado et al. (29)	2019	Barbados	Interrupted time series	Weekly sales of SSBs in mL/capita*	−0.19 [−0.47; 0.08]	200 weeks	1 observation per week
Cawley et al. (15)	2020	Philadelphia	Difference-in-difference	Taxed beverages monthly purchases (in ounces)*	−0.17 [−0.27; −0.06]	1447 households	12 observations per household
Cawley et al. (40)	2020	Oakland	Difference-in-difference	Volume purchased of taxed beverages in ounces*	−0.07 [−0.18; 0.03]	1360 individuals	2 observations per individual
Bleich et al. (39)	2021	Philadelphia	Difference-in-difference	Purchased fluid ounces of taxed beverages*	−0.16 [−0.24; −0.08]	2369 purchases	2 observations per purchase
Kruz et al. (14)	2020	Hungary	Synthetic control	SSB sales in milliliters*	0.91 [−0.05; 1.86]	16 regions	15 observations per region
Kruz et al. (14)	2020	France	Synthetic control	SSB sales in milliliters*	−0.11 [−1.03; 0.82]	16 regions	15 observations per region
Silver et al. (30)	2017	Berkeley	Interrupted time series	% change in volume of taxed beverages sold per transaction relative to counterfactual developed based on pre-intervention trends*	−0.08 [−1.24; 1.08]	9 stores	1128 observations per store
Aguilar Esteva et al. (17)	2019	Mexico	Regression discontinuity design	Total calories contained in all purchased taxed drinks*	−0.05 [−0.10; −0.00]	6935 households	104 observations per household
Øvrebø et al. (33)	2020	Norway	Interrupted time series	Exponentiated (Log) of sale of liters of soda*	0.01 [−0.06; 0.07]	3884 stores	50 observations per store
Rojas and Wang (21)	2021	Seattle	Difference-in-difference	Log volume purchased of taxed SSBs*	−0.02 [−0.04; −0.01]	61139 brands in a region	36 observations for brands in a region
Rojas and Wang (21)	2021	Berkley	Difference-in-difference	Log volume purchased of taxed SSBs*	−0.10 [−0.17; −0.02]	2548 brands in a region	24 observations for brands in a region
Colchero et al. (41)	2016	Mexico	Difference-in-difference and Fixed effects	Log volume purchased (mL/capita/day) of taxed beverages*	−0.07 [−0.13; −0.02]	5698 households	36 observations per household
Colchero et al. (43)	2016	Mexico	Difference-in-difference and Fixed effects	Log of volume purchased (mL/capita/day) of taxed beverages (high SES)	−0.03 [−0.11; 0.04]	2686 households	27 observations per household
Colchero et al. (41)	2016	Mexico	Difference-in-difference and Fixed effects	Log of volume purchased (mL/capita/day) of taxed beverages (middle SES)	−0.09 [−0.15; −0.03]	3885 households	27 observations per household
Colchero et al. (43)	2016	Mexico	Difference-in-difference and Fixed effects	Log of volume purchased (mL/capita/day) of taxed beverages (low SES)	−0.15 [−0.26; −0.05]	1375 households	27 observations per household
Law et al. (31)	2021	India	Interrupted time series	Year-on-year growth rate change trend in % volume aerated drinks sold—change in linear time coefficient	0.83 [−0.15; 1.80]	15 regions	51 observations per region
Law et al. (31)	2021	India	Interrupted time series	Year-on-year growth rate change in % volume aerated drinks sold—change in quadratic time coefficient	−0.77 [−1.74; 0.20]	15 regions	51 observations per region
Law et al. (31)	2021	India	Interrupted time series	Year-on-year growth rate change in % volume aerated drinks sold - change in linear time coefficient in high income states	0.19 [−0.77; 1.14]	15 regions	51 observations per region
Law et al. (31)	2021	India	Interrupted time series	Year-on-year growth rate change in % volume aerated drinks sold - change in quadratic time coefficient in high income states	−0.13 [−1.09; 0.82]	15 regions	51 observations per region
Law et al. (31)	2021	India	Interrupted time series	Year-on-year growth rate change in % volume aerated drinks sold - change in linear time coefficient in low income states	0.79 [−0.18; 1.76]	15 regions	51 observations per region
Law et al. (31)	2021	India	Interrupted time series	Year-on-year growth rate change in % volume aerated drinks sold—change in quadratic time coefficient in low income states	−0.75 [−1.73; 0.22]	15 regions	51 observations per region
**Purchases of high and medium tax beverages**							
Nakamura et al. (36)	2018	Chile	Fixed effects	Log ml purchases high tax soft drinks*	−0.21 [−0.28; −0.13]	2836 households	60 observations per household
Nakamura et al. (36)	2018	Chile	Fixed effects	Log ml purchases of high tax soft drinks (low SES)	−0.01 [−0.13; 0.11]	1120 households	60 observations per household
Nakamura et al. (36)	2018	Chile	Fixed effects	Log ml purchases of high tax soft drinks (middle SES)	−0.15 [−0.28; −0.03]	963 households	60 observations per household
Nakamura et al. (36)	2018	Chile	Fixed effects	Log ml purchases of high tax soft drinks (high SES)	−0.29 [−0.40; −0.17]	1138 households	60 observations per household
Gonçalves and Pereira dos Santos (20)	2020	Portugal	Difference-in-difference and Fixed effects	ln (Liters sold of High Sugar products)*	−0.01 [−0.04; 0.02]	17999 stores	36 observations per store
Gonçalves and Pereira dos Santos (20)	2020	Portugal	Difference-in-difference	ln (Liters sold of Medium Sugar products)	0.00 [−0.03; 0.03]	15772 stores	36 observations per store
Pell et al. (34)	2020	United Kingdom	Interrupted time series	Absolute change (ml/g) purchases of high tax beverages per household	0.21 [−0.06; 0.47]	212 weeks	1 observation per week
**Purchases of low tax beverages**							
Gonçalves and Pereira dos Santos (20)	2020	Portugal	Difference-in-difference	ln (Quantity of liters sold of Low Sugar products)*	−0.03 [−0.05; −0.01]	30762 households	36 observations per household
Nakamura et al. (36)	2018	Chile	Fixed effects	Log ml purchases of low tax soft drinks*	0.02 [−0.06; 0.09]	2836 households	60 observations per household
Nakamura et al. (36)	2018	Chile	Fixed effects	Log ml purchases of low tax soft drinks (low SES)	−0.10 [−0.22; 0.02]	1120 households	60 observations per household
Nakamura et al. (36)	2018	Chile	Fixed effects	Log ml purchases of low tax soft drinks (middle SES)	0.11 [−0.02; 0.23]	963 households	60 observations per household
Nakamura et al. (36)	2018	Chile	Fixed effects	Log ml purchases of low tax soft drinks among (high SES)	0.05 [−0.70; 0.16]	1138 households	60 observations per household
Pell et al. (34)	2020	England	Interrupted time series	Absolute change (ml/g) purchases of low tax beverages per household	−0.51 [−0.78; −0.24]	212 weeks	1 observation per week
**Purchasing of untaxed food and beverages**							
Cawley et al. (15)	2020	Philadelphia	Difference-in-difference	Untaxed beverages monthly purchases (in ounces)*	−0.09 [−0.19; 0.02]	1447 households	12 observations per household
Cawley et al. (40)	2020	Oakland	Difference-in-difference	Volume purchased of untaxed beverages in ounces*	0.03 [−0.07; 0.14]	1363 individuals	2 observations per individual
Nakamura et al. (36)	2018	Chile	Fixed effects	Log of per capita volume of no-tax soft drink purchased by the household*	−0.07 [−0.15; 0.00]	2836 households	60 observations per household
Bleich et al. (39)	2021	Philadelphia	Difference-in-difference	Purchased fluid ounces of nontaxed beverages*	−0.01 [−0.09; 0.07]	2369 purchases	2 observations per purchase
Powell and Leider (35)	2021	Seattle	Difference-in-difference	Percent grams sugar sold from untaxed beverages relative to comparator*	0.00 [−0.25; 0.25]	239 brands	2 observations per brand
Powell and Leider (35)	2020	Cook County	Interrupted time series	Untaxed beverage volume sold in Cook County, Illinois, relative to St Louis County and City, Missouri—change in level after tax	0.27 [−0.07; 0.60]	138 weeks	1 observation per week
Powell and Leider (35)	2020	Cook County	Interrupted time series	Untaxed Beverage Volume Sold in Cook County, Illinois, Relative to St Louis County and City, Missouri—change in slope after tax	−0.21 [−0.54; 0.12]	138 weeks	1 observation per week
Powell and Leider (35)	2020	Cook County	Interrupted time series	Untaxed beverage volume sold in Cook County, Illinois, relative to St Louis County and City, Missouri—change in level after tax repeal	−0.08 [−0.62; 0.46]	51 weeks	1 observation per week
Powell and Leider (35)	2020	Cook County	Interrupted time series	Untaxed beverage volume sold in Cook County, Illinois, relative to St Louis County and City, Missouri—change in slope after tax repeal	0.36 [−0.18; 0.91]	51 weeks	1 observation per week
Gonçalves and Pereira dos Santos (20)	2020	Portugal	Difference-in-difference	ln (Liters sold of zero sugar products)*	0.01 [−0.03; 0.04]	13864 stores	36 observations per store
Aguilar Esteva et al. (17)	2019	Mexico	Regression discontinuity design	Calories purchased in untaxed drinks*	0.01 [−0.04; 0.06]	6935 households	104 observations per household
Aguilar Esteva et al. (17)	2019	Mexico	Regression discontinuity design	Calories purchased from untaxed food	0.02 [−0.03; 0.07]	6935 households	104 observations per household
Colchero et al. (43)	2016	Mexico	Difference-in-difference and Fixed effects	Volume purchased (mL/capita/day) of untaxed beverages*	−0.07 [−0.13, −0.02]	5698 households	36 observations per household
Silver et al. (30)	2017	Berkeley	Interrupted time series	% change in volume of nontaxed beverages sold per transaction relative to counterfactual developed based on pre-intervention trends*	0.29 [−0.88; 1.45]	9 stores	1128 observations per store
Alvarado et al. (29)	2019	Barbados	Interrupted time series	Absolute difference in beverages that are not water or SSBs purchased ml/capita in the final week*	0.30 [0.03; 0.58]	200 weeks	1 observation per week
Puig-Codina et al. (42)	2020	Catalonia	Synthetic control	Liters diet cola purchased per person per month*	0.62 [−0.30; 1.53]	17 regions	78 observations per region
Pell et al. (34)	2020	United Kingdom	Interrupted time series	Absolute change (ml/g) purchases of untaxed beverages per household	0.76 [0.49; 1.03]	212 weeks	1 observation per week
Chakrabarti et al. (22)	2016	India	Difference-in-difference	Month household consumption (purchases) of all pulses (kg/household/month)	0.02 [0.01; 0.03]	112750 households	2 observations per household
Chakrabarti et al. (22)	2016	India	Difference-in-difference	Month household consumption (purchases) of all pulses (kg/household/month) in Himachal Pradesh	0.02 [0.01; 0.03]	112750 households	2 observations per household
Chakrabarti et al. (22)	2016	India	Difference-in-difference	Month household consumption (purchases) of all pulses (kg/household/month) in Punjab	0.01 [0.00, 0.02]	112750 households	2 observations per household
Chakrabarti et al. (22)	2016	India	Difference-in-difference	Month household consumption (purchases) of all pulses (kg/household/month) in Andhra Pradesh	0.02 [0.01, 0.03]	112750 households	2 observations per household
Chakrabarti et al. (22)	2016	India	Difference-in-difference	Month household consumption (purchases) of all pulses (kg/household/month) in Tamil Nadu	0.02 [0.01; 0.04]	112750 households	2 observations per household
**Purchase of untaxed beverages among high SES households**							
Nakamura et al. (36)	2018	Chile	Fixed effects	Log of per capita volume of untaxed soft drink purchased by the household*	−0.17 [−0.29; −0.06]	1138 households	60 observations per household
Colchero et al. (41)	2016	Mexico	Difference-in-difference and Fixed effects	Volume purchased (mL/capita/day) of untaxed beverages*	0.00 [−0.08; 0.08]	2336 households	27 observations per household
**Purchase of untaxed beverages among middle SES households**							
Nakamura et al. (36)	2018	Chile	Fixed effects	Log of per capita volume of untaxed soft drink purchased by the household*	0.05 [−0.07; 0.18]	963 households	60 observations per household
Colchero et al. (43)	2016	Mexico	Difference-in-difference and Fixed effects	Volume purchased (mL/capita/day) of untaxed beverages*	−0.09 [−0.015, −0.03]	3885 households	27 observations per household
**Purchase of untaxed beverages among low SES households**							
Nakamura et al. (36)	2018	Chile	Fixed effects	Log of per capita volume of untaxed soft drink purchased by the household*	−0.51 [−0.63; −0.39]	1120 households	60 observations per household
Colchero et al. (41)	2016	Mexico	Difference-in-difference and Fixed effects	Volume purchased (mL/capita/day) of untaxed beverages*	−0.07 [−0.18; 0.04]	1375 households	27 observations per household
**Purchase of untaxed, high-sugar food**							
Bleich et al. (39)	2021	Philadelphia	Difference-in-difference	Total calories from high-sugar food purchases*	0.02 [−0.06; 0.10]	2369 purchases	2 observations per purchase
Powell et al. (26)	2021	Seattle	Difference-in-difference	Percent grams sugar sold from sweets relative to comparator*	0.22 [0.13; 0.31]	2054 brands	2 observations per brand
**Purchase of taxed, high-sugar food**							
Aguilar Esteva et al. (17)	2019	Mexico	Regression discontinuity design	Total calories contained in all purchased taxed food*	−0.04 [−0.09; 0.00]	6935 households	104 observations per household
Øvrebø et al. (33)	2020	Norway	Interrupted time series	Exponentiated log of sale of candy sold (kg)*	0.01 [−0.06; 0.07]	3884 stores	50 observations per store
**Diet quality**							
Cawley et al. (15)	2020	Oakland	Difference-in-difference	Consumption of grams of added sugar in adults*	−0.09 [−0.30; 0.12]	341 individuals	2 observations per individual
Cawley et al. (40)	2020	Oakland	Difference-in-difference	Consumption of grams of added sugar in children	0.01 [−0.21; 0.23]	318 individuals	2 observations per individual
Royo-Bordonada et al. (24)	2019	Catalonia	Difference-in-difference	Ratio of post to pre-tax prevalence of regular taxed beverages (soft drinks, fruit drinks, energy drinks)*	0.45 [0.36; 0.54]	1929 individuals	2 observations per individual
Royo-Bordonada et al. (24)	2019	Catalonia	Difference-in-difference	Ratio of post to pre-tax prevalence of regular consumption of untaxed beverage	0.61 [0.52; 0.70]	1929 individuals	2 observations per individual
Chakrabarti et al. (22)	2016	India	Difference-in-difference	Daily household intake of proteins (gm/day)*	0.01 [0.00; 0.02]	112750 households	2 observations per household
Howard and Prakash (28)	2011	United States	Instrumental variable	Servings of fruit in previous week*	0.03 [−0.03; 0.08]	5140 individuals	1 observation per individual
Øvrum and Bere (18)	2013	Norway	Randomized control trial	Portions of fruits or vegetables consumed per day *	0.22 [0.11; 0.34]	1149 individuals	1 observation per individual
**Health outcomes**							
Chakrabarti et al. (25)	2019	Punjab	Difference-in-difference	Hemoglobin*	−0.01 [−0.11; 0.09]	1587 individuals	2 observations per individual
		Tamil Nadu		Hemoglobin*	−0.00 [−0.07; 0.07]	3089 individuals	2 observations per individual
Hernández-F et al. (32)	2021	Mexico	Interrupted time series	Intercept change in outpatient visits related to dental caries	0.66 [0.34; 0.98]	156 months	1 observation per month
Hernández-F et al. (32)	2021	Mexico	Interrupted time series	Slope change in outpatient visits related to dental caries	−0.81 [−1.13; −0.49]	156 months	1 observation per month
**Other outcomes**							
Bleich et al. (39)	2021	United States	Difference-in-difference	Total calories from sweetened beverage and high-sugar food purchases	−0.09 [−0.17; −0.01]	2369 purchases	2 observations per purchase

*Indicates estimates included in meta-analysis.

#### Effects of taxes

##### Taxes on sugar-sweetened beverages have no overall effect on purchasing of beverages

**FIGURE 4:**
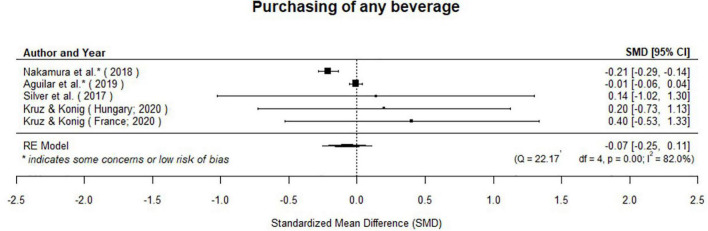
Taxes on SSBs have no overall effect on purchasing of beverages.

The meta-analysis suggests taxes on SSBs have no overall effect on beverage purchases $(\hat{\mu }=-0.07\left(95\%\mathrm{CI}:-0.25\text{to}0.11\right);p=0.42;$Figure 4). We included five effect estimates from four unique studies. Studies considered calories purchased in beverages (17), sugar purchased in beverages (36), and volume of beverages purchased (14, 30).

There was significant heterogeneity in results (*Q*(4) = 22.17, *p* = 0.001, ${\hat{\tau }}^{2}=0.02$, *I*^2^ = 81.96%). However, results did not differ between studies that considered volume and those that considered other outcomes (−0.14 [95% CI: −0.51 to 0.24], *p* = 0.48). There was no variation in results among studies that used synthetic control methods or interrupted time series and computationally similar methods (0.40 [95% CI: −0.31 to 1.11], *p* = 0.27), or between studies scored as high risk of bias and some risk of bias (−0.37 [95% CI: −0.99 to 0.25]; *p* = 0.25).

We examined the studentized residuals and found that two studies (17, 36) had values larger than 2.58 and may be potential outliers in the context of this model. Results did not change meaningfully when (36) was dropped from analysis ($\hat{\mu }=$0.01 [95% CI: −0.05 to 0.04]; *p* = 0.76), but became significant when (17) was removed from analysis $\hat{\mu }=-0.20\left[95\%CI:-0.28\text{to}-0.13\right];p=0.001)$.

##### Taxes on beverages may reduce purchases of taxed beverages

**FIGURE 5:**
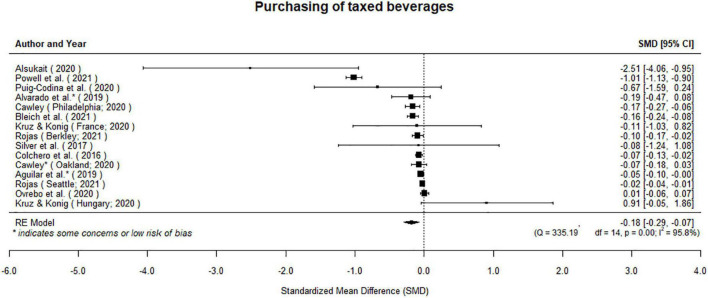
Taxes on beverages may reduce purchases of taxed beverages.

The evidence from twelve studies (with 15 independent effect estimates) suggests taxes on SSBs reduced consumers purchasing such beverages ($\hat{\mu }=-0.18\left[95\%CI:-0.29\text{to}-0.07\right];p=0.001$; Figure 5). We examined the studentized residuals and found that one study (26) had a value larger than 2.94 and may be a potential outlier in the context of this model. Our results remain significant when we removed this effect from the analysis ($\hat{\mu }=-0.07\left[95\%CI:-0.12\text{to}0.03\right];p=0.001)$. Neither the rank correlation nor the regression test indicated any funnel plot asymmetry (*p* = 0.38; *p* = 0.29, Supplementary material 5, Figure 1), indicating that publication bias was not present.

Powell and Leider (35), which evaluated a tax on sugar-sweetened and artificially sweetened beverages in Cook County, IL, United States, report that, after the implementation of the tax, purchases of taxed beverages reduced immediately (*g* = −1.30 [95% CI: −1.65 to −0.96]) but there was no change in trend in purchase patterns (*g* = −0.08 [95% CI: −0.41 to 0.25]). When the county government repealed the tax, consumption increased (*g* = 1.09 [95% CI: 0.53 to 1.65]) but, once again, there was no change in trend in consumption patterns (*g* = 0.22 [95% CI: −0.32 to 0.77]). Law et al. (31), which evaluated changes in take-home aerated soft drink purchases after implementation of India’s Goods and Services Tax (GST), consider linear and quadratic trend changes in the sale of taxes, aerated beverages. They find no change in either coefficient (*g* = 0.83 [95% CI: −0.15 to 1.8] and *g* = −0.77 [95% CI: 1.74 to 0.2], respectively). We could not consider these results within the main analysis because study authors report multiple measures within the same regression.

##### Taxes on sugar-sweetened beverages have no effect on purchases of untaxed beverages

**FIGURE 6:**
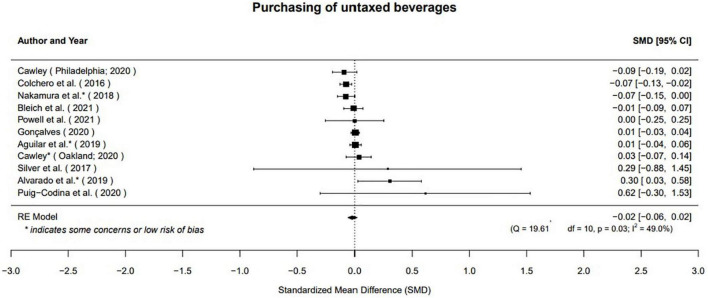
Taxes on SSBs beverages have no effect on purchases of untaxed beverages.

Based on evidence from 11 studies, our meta-analysis suggests there is no effect of taxes on SSBs on the purchasing of untaxed beverages $(\hat{\mu }=0.02\left(95\%CI:-0.06\text{to}0.02\right);p=0.33;$Figure 6). Studies considered calories purchased (17), grams of sugar purchased (26), volume purchased (15, 20, 29, 30, 36, 39–42). There was moderate heterogeneity in results (*Q*(10) = 19.61, *p* = 0.03, ${\hat{\tau }}^{2}=0.00$, *I*^2^ = 49.01%). However, impacts were generally, consistently null or very small. There was no difference in impacts between those that considered volume of purchases and those that considered other outcomes (−0.03 [95% CI: −0.12 to 0.06], *p* = 0.46). Results were not different between studies that used difference-in-difference approaches when compared to those that used either synthetic control, interrupted time series, or computationally similar approaches (0.02 [95% CI: −0.06 to 0.11], *p* = 0.59). Similarly, results were not different among studies we assessed as high risk of bias and the other studies (0.03 [95% CI: −0.04 to 0.11]; *p* = 0.40).

We examined the studentized residuals and found that none of the studies had a value larger than ±2.84 and hence there was no indication of outliers in the context of this model. Neither the rank correlation nor the regression test indicated any funnel plot asymmetry (*p* = 0.45 and *p* = 0.08, respectively, Supplementary material 5, Figure 2), indicating that there was no publication bias present.

Powell and Leider (35) find a tax on sugar-sweetened and artificially sweetened beverages had no effect on purchases of untaxed beverages, nor do they find an effect of repealing the tax. However, Pell et al. (34) find that the *announcement* of England’s tax on SSBs increased purchases of untaxed beverages (*g* = 0.76 [95% CI: 0.49 to 1.03]). We did not consider these results within the main analysis because study authors evaluated the *announcement* of the tax, rather than an implemented fiscal policy.

##### The evidence is too limited to draw conclusions about effects of taxes on sugar-sweetened beverages on the purchasing of untaxed high-sugar foods

Two studies reported on the impacts of standalone SSB taxes on sugary food consumption and find conflicting results. Bleich et al. (39) find that there is no change in the total calories purchased from high-sugar-foods (*g* = 0.02 [95% CI: −0.06 to 0.10]) in Philadelphia, PA, United States. However, Powell et al. (26) find an increase in the amount of sugar sold in sweets (*g* = 0.22 [95% CI: 0.13 to 0.31]) in Seattle, WA, United States. When we consider these effects jointly, we find no effect on the purchasing of sugary foods $\hat{(\mu }=0.12$ [95% CI: –0.08 to 0.32]; *p* = 0.23, Supplementary material 5, Figure 3). However, because the evidence base is limited and heterogeneous, we cannot make a definitive conclusion.

##### The evidence base is too limited to draw conclusion about effects of taxes on sugar-sweetened beverages have on evaluated measures of diet quality

Two studies reported on the impacts of SSB on measures of diet quality, each reporting two effects. Cawley et al. (15) find that the consumption of added sugar did not change in adults (*g* = −0.09 [95% CI: −0.30 to 0.12]) or children (*g* = 0.01 [95% CI: −021 to 0.23]) across several cities in the United States. Royo-Bordonada et al. (24) find no change in the consumption of taxed (*g* = 0.45 [95% CI: 0.36 to 0.54]) or untaxed (*g* = 0.61 [95% CI: 0.52 to 0.70]) beverages in Catalonia, Spain. When the change in taxed beverage and sugar consumption of adults were pooled, we find no overall effect $\hat{(\mu }=0.19$ [95% CI: –0.34 to 0.72]; *p* = 0.48; Supplementary material 5, Figure 4). However, the evidence is too limited to make a definitive conclusion.

##### The evidence base is too limited to draw conclusions about effects of taxes on sugar-sweetened beverages and high-sugar foods on the frequency of dental visits

Mexico’s tax on SSBs and energy dense foods resulted in an immediate increase in outpatient visits for dental carries (*g* = 0.66 [95% CI: 0.34 to 0.98]) but an overall decrease in the trend in the frequency of these visits (*g* = −0.81 [95% CI: −1.13 to −0.49]; (32)). Because these results were based on a single study, they should be interpreted with caution.

##### The evidence base is too limited to draw conclusions about effects of taxes on sugar-sweetened beverages and high-sugar foods on the purchasing of taxed foods

There were no effects of taxes on high-sugar foods and SSBs in Mexico or Norway. In Mexico, Aguilar Esteva et al. (17) find no effect on total calories purchased (*g* = −0.04 [95% CI: −0.09 to 0.00]). In Norway, Øvrebø et al. (33) find no change in the sale of candy (*g* = 0.01 [95% CI: −0.06 to 0.07]). When considered jointly, these results suggest no change in the purchasing of sugary foods $\hat{(\mu }=-0.02$ [95% CI: –0.07 to 0.02]; *p* = 0.34; Supplementary material 5, Figure 5). However, the evidence is very limited and should be interpreted with caution.

##### Sub-group analysis of effects of taxes

**FIGURE 7:**
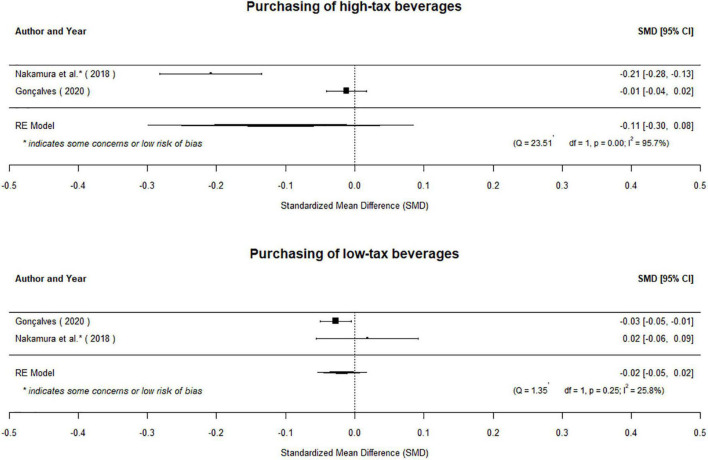
The evidence base is too limited to draw conclusions about the effects of high-tax and low-tax beverages on purchases.

Although two studies interrogate the impacts of high and low tax levels on purchasing patterns, the evidence base is too limited to make definitive conclusions. Gonçalves and Pereira dos Santos (20) find no change in purchases of taxed beverages as a result of high- (*g* = −0.01 [95% CI: −0.04 to 0.02]) or middle-tier (*g* = 0.00 [95% CI: −0.03 to 0.03]) taxes in Portugal. The report a small reduction in purchases of low-tax beverages (*g* = −0.03 [95% CI: −0.05 to −0.01]). However, Nakamura et al. (36) find that a high-tier tax resulted in a reduction in purchases of high-tax beverages (*g* = −0.21 [95% CI: −0.28 to −0.13]) and no change in purchases of low-tax beverages (*g* = 0.02 [95% CI: −0.06 to 0.09]) in Chile. No middle-tier tax was imposed in Chile. When pooled, there was no effect on purchases of the high-tax beverages ($\hat{\mu }=-0.11\left[95\%\text{CI}:-0.30\text{to}0.09\right];p=0.27)$ or low-tax beverages ($\hat{\mu }=-0.02\left[95\%\text{CI}:-0.05\text{to}0.02\right];p=0.30;$ Figure 7). A third study, Pell et al. (34) evaluated the *announcement* of England’s tiered tax on SSBs, which also had no effect on purchases of high-sugar beverages (*g* = 0.21 [95% *CI:*−0.06 to 0.47]) but decreased the purchases of low-sugar beverages (*g* = −0.51 [95% CI: −0.78 to −0.24]). However, we did not consider these results within the main analysis because study authors evaluated the *announcement* of the tax, rather than an implemented fiscal policy. Given the conflicting findings from the limited evidence base, the effects of these taxes are unclear.

**FIGURE 8:**
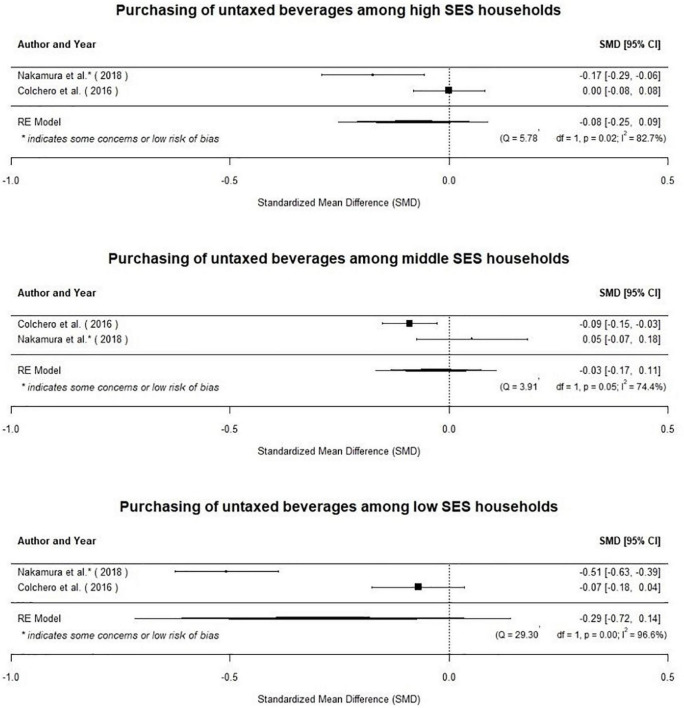
The evidence base is too limited to draw conclusions about the effects of taxes by socio-economic status.

Similarly, the evidence is too limited to draw any conclusions about differential effects across socioeconomic status (SES) groups. Two studies assessed the effects of SSB taxes on consumption of taxed and untaxed beverages by socioeconomic status. Colchero et al. (41) found that, in Mexico, the tax resulted in a no change in SSB purchases among individuals in the highest socioeconomic levels (*g* = −0.03 [95% CI: −0.11 to 0.04]). However, purchases decreased slightly in the lowest (*g* = −0.15 [95% CI: −0.26 to −0.05]) and middle (*g* = −0.09 [95% CI: −0.15 to −0.03]) socioeconomic levels. Nakamura et al. (36) find that consumption of high-tax beverages decreased among middle (*g* = −0.15 [95% CI: −0.28 to −0.03]) and higher (*g* = −0.29 [95% CI: −0.40 to −0.17]) socioeconomic levels but did not change among the lowest socioeconomic class (*g* = −0.01 [95% CI: −0.13 to 0.11]). There was no change in consumption of low-tax beverages in any socioeconomic class. When considered jointly, these two studies show that there was no change in the purchases of untaxed beverages among high (μ = −0.08[95%CI: −0.25 to 0.09]; *p* = 0.35), middle (μ = −0.03[95%CI: −0.17 to 0.11]; *p* = 0.67), or low (μ = −0.29[95%CI: −0.72 to 0.14]; *p* = 0.19; Figure 8). However, given the limited and heterogeneous evidence base, the variation in effects of taxes across socioeconomic classes remains uncertain.

#### Effects of subsidies

Chakrabarti et al. (22) find that a subsidy for pulses in India increased purchases of pulses (μ = 0.02 [95% CI: 0.01 to 0.03]; *p* < 0.001; Supplementary material 5, Figure 6A). Effects were too small to be meaningful; however, they were generally consistent across the four states considered (Himachal Pradesh, Punjab, Andhra Pradesh, and Tamil Nadu, *Q*(3) = 3.05, *p* = 0.38, ${\hat{\tau }}^{2}=0.00,$ *I*^2^ = 1.60%). Because these results are based on a single study, they should be interpreted with caution.

Only one study considered the impacts of subsidies to support a diverse diet on health. Chakrabarti et al. (25) consider the impacts of a subsidy for iron-fortified wheat on hemoglobin levels in Punjab (*g* = −0.01 [95% CI: −0.11 to 0.09]) and Tamil Nadu (*g* = −0.00 [95% CI: −0.07 to 0.07]), India. When considered jointly, the subsidies have no effect on hemoglobin levels ($(\hat{\mu }=-0.005$ [95% CI: –0.06 to 0.05]; *p* = 0.88; Supplementary material 5, Figure 6B). However, since these results also come from a single study, they are also inconclusive.

Subsidies that incentivize diversifying diet with vegetables, fruits, or pulses have no effect on diet quality $(\hat{\mu }=0.06$ [95% CI: –0.01 to 0.14]; *p* = 0.10; Supplementary material 5, Figure 6C) in the included studies. We included three effect estimates, all from unique studies in this meta-analysis. The specific outcomes reported are daily household intake of protein [g/day; (22)], intake of fruits (excluding fruit juices) and vegetables (excluding potatoes) consumed on a typical day (18); and servings of fruit in the previous week (28). According to the *Q*-test, there was significant heterogeneity in the true outcomes (*Q*(2) = 12.79, *p* = 0.01, ${\hat{\tau }}^{2}=0.00$, *I*^2^ = 84.37%). We examined the studentized residuals and found that study (18) had a value larger than ±2.39 and may be a potential outlier in the context of this model. When it was removed, the estimate becomes statistically, but not practically, significant $(\hat{\mu }=0.01$ [95% CI: 0.002 to 0.02]; *p* = 0.03) According to the Cook’s distances, none of the studies could be considered to be overly influential. Given the limited number of studies, this finding needs to be interpreted with caution.

### Design and implementation considerations

We summarize qualitative findings for both analyzed and linked studies in the following sections.

#### How are taxes on sugar-sweetened beverages, high sugar foods or aerated beverages ‘supposed’ to work?

Despite the call to implement these taxes because of their effects on health and wellbeing, few authors include health outcomes, such as obesity and diabetes, in their theories of change (17, 20, 21, 30, 42). Instead, most of the hypothesized theories of change focus on consumption. Authors argue that taxes on SSBs, sugary foods and aerated beverages will increase prices of taxed goods and decrease their consumption, but do not link these primary outcomes to health and wellbeing. In general, we observed three theories for how changes in price may affect consumption of SSBs or high-sugar foods: (1) signaling effects of adverse health risks, (2) price elasticity and substitution effects, or (3) product reformulation.

The ‘signaling effect’ mechanism suggests that the publicizing and adoption of taxes with associated price increases of taxed foods and beverages communicates to consumers that these products are inferior to untaxed products. Awareness of health risks associated with their consumption may influence consumers to reduce purchases (14, 15, 24, 34, 35, 44, 45). Two studies reported that signaling effects from taxes were amplified by information campaigns that may have increased awareness of the health risks associated with calorie-dense foods and beverages that are high in sugar, fat or salt (35, 46). Pell et al. (34) also suggest signaling effects and found that the *announcement* of England’s tax on SSBs increased purchases of untaxed beverages before the policy went into effect.

Several authors observe theorized ‘price elasticity effects’ and conclude that raising the prices of taxed goods discourages purchases (14–16, 21, 29, 30, 33, 40–42). Others argue that consumers may purchase untaxed goods instead, substituting their purchases of taxed goods for untaxed goods (16, 47–50). Alsukait et al. (16) suggest decreased SSB consumption may increase the sale and consumption of bottled water; Taillie et al. (50) anticipate substitution effects from taxed to untaxed goods; Edmondson et al. (47) hypothesize that decreased consumption of taxed beverages will increase sales of juice or milk; and Leider and Powell (49) argue that taxes will increase in sales of other snacks, untaxed beverages or alcohol. Although many authors anticipated changes in purchases of untaxed goods, we find no change in purchases of untaxed beverages (Figure 6).

The final theorized mechanism from taxes to nutritious diets is through manufacturer product reformulation to reduce sugar (20, 34, 51). Both Portugal and the United Kingdom implemented tiered taxes that levied higher taxes for high-sugar products relative to low-sugar products. Gonçalves and Pereira dos Santos (20) and Pell et al. (52) observed that SSB manufacturers in both countries reformulated products to reduce added sugars to pay a lower tax rate. Industry reformulation was also observed in Mexico, which had a progressive tax (51). In contexts where volume of purchases of SSBs or high-sugar foods did not change, product reformulation may still have achieved changes in consumption of free sugars.

#### How are subsidies supposed to work?

Unlike proposed mechanisms of action for taxes, studies considering subsidies generally posit an effect on diet quality and health. Studies of subsidies for nutritious foods suggest the main mechanism of action is that they reduce prices, causing an increase in the purchases and consumption of foods, leading to improved nutrition outcomes (18, 22, 25, 28). For example, Chakrabarti et al. (25) argue that subsidies for iron-fortified wheat flour can reduce incidence of anemia in pregnant women. Chakrabarti et al. (22) posit that subsidies for pulses can improve nutritional outcomes for below-poverty-line families. While there was limited evidence, the studies considering relevant outcomes found that subsidizing pules increased household purchases and daily protein intake. However, the limited evidence does not suggest that subsidies for iron-fortified wheat flour impact anemia.

#### Do fiscal policies to encourage diverse diets have (unintended) regressive effects?

Consumers in lower socioeconomic classes may be more responsive to changes in prices than those in who are better off because changes in price are likely to represent larger relative changes in their total expenditures. This reasoning posits that fiscal policies may have variable impacts across socioeconomic levels. However, with few studies investigating impact across socioeconomic level and heterogeneous impacts, variation in impacts by socioeconomic level is uncertain. There were relatively larger decreases in consumption of taxed products among low-income households relative to high-income and middle-income households in Mexico and Philadelphia (39, 41). Nakamura et al. (36) report that SSB taxes decreased consumption among high- and middle-income households, but did not detect any change in consumption among the lowest socioeconomic class in Chile. Fichera et al. (53) did not observe any impact by socioeconomic status. Most studies did not have the income data necessary to conduct differential analysis by income status. Given the variability of results, it is not possible to determine if the hypothesized variation in impacts across socioeconomic classes occurs.

Only a handful of studies reported differential analysis for other relevant groups. Bleich et al. (39) reported findings for education completed; Cawley et al. (15) and Teng et al. (37) reported findings for both adults and children; Chakrabarti et al. (25) reported findings for women at high-risk for anemia; Nakamura et al. (36) reported findings by weight.

Fiscal policies had slightly more positive effect sizes in HICs relative to L&MICs. In HICs, ten out of 20 studies report impact on consumer purchases in response to taxes. In L&MICs, two studies out of six report impact on consumer purchases in response to a tax or subsidy. There were not enough studies in L&MICs to conduct moderator analysis by country income level.

#### Facilitators of fiscal policies

Based on thematic analysis of all 49 impact evaluations of eligible interventions, we identified key factors that may facilitate the implementation and effectiveness of fiscal policies. Positive effects of fiscal policies were facilitated by (1) information to increase awareness on adverse health effects of SSBs or high-sugar foods and health benefits of subsidized foods, (2) large geographic coverage, and (3) the potential for revenue generation.

##### Information and media campaigns delivered in conjunction with the rollout of fiscal policies may facilitate beneficial effects on consumption and diet

Media campaigns may have increased awareness of adverse health effects of consumption of SSBs or high-sugar foods and health benefits of consumption of subsidized foods (21, 25, 33, 35, 40, 51). A pro-SSB tax campaign, “Berkeley vs. Big Soda,” focused on adverse health outcomes and the “inappropriate behavior in the SSB industry” (46). The campaign’s success was attributed to endorsements from a wide range of supporters, early stage coalition building, and prominent features by community representatives.

Better information may also amplify signaling effects and facilitate policy implementation. Chakrabarti et al. (25) report that new guidelines on safe levels of iron, folic acid and vitamin B12 published in 2016 may have encouraged two state governments in India to implement fortified wheat subsidies through an existing food subsidy program, the Public Distribution System (PDS). On the other hand, Law et al. (31) suggest that the focus of India’s tax on revenue generation rather than health may have led to the null effects.

However, pro-tax campaigns are susceptible to retaliation from manufacturers. Powell and Leider (35) found that powerful manufacturer-backed anti-tax campaigns influenced the quick repeal of the Cook County, Illinois SSB tax just 4 months post-implementation. In June 2018, California state lawmakers passed a bill to prevent any municipality from passing a beverage or food tax for the next 12 years (54). Implementers should anticipate engaging with manufactures of taxed products.

##### Taxes covering larger geographic areas may facilitate compliance

Several studies report that a larger geographic coverage makes it more difficult for consumers to avoid SSB or high-sugar food taxes by cross-border shopping (15, 21, 23, 39, 55–57). Rojas and Wang (21) observed that implementing the tax in Berkley, CA, United States resulted in smaller changes in consumption relative to taxes implemented in larger geographic areas such as Seattle, WA, United States. Taxes that cover larger geographic areas limit opportunities for shopping in nearby, tax free locations, facilitating compliance and changes in consumer purchasing behaviors away from foods and beverages that are high in sugar, fat or salt.

##### Highlighting the potential for tax revenue may facilitate implementation by local governments

When framed by their revenue-generating potential, taxes experienced greater buy-in from implementing governments at local [Cook County, IL as observed by Powell and Leider (35)], state [Catalonia, Spain as observed by Puig-Codina et al. (42)] and national [Portugal, as observed by Gonçalves and dos Santos (20) and Norway, as observed by Øvrebø et al. (33)] levels of governance. While taxes on candy and SSBs in Norway were mainly implemented to create revenues, health benefits were later emphasized by the government (33).

#### Barriers to effects of fiscal policies

We identified several barriers to the implementation and effectiveness of fiscal policies including (1) tax avoidance through cross-border shopping, (2) vendor non-compliance, and (3) low awareness of health risks associated with SSBs, high-sugar foods or aerated beverages. Subsidy evaluations did not report any barriers to implementation.

##### Opportunities for cross-border shopping may limit compliance and provide a barrier to changing consumer habits toward nutritious diets

Cross-border shopping or tax avoidance is a barrier to compliance with the taxes on SSBs or high-sugar foods (15, 21, 23, 39, 40, 56, 57). For instance, Cawley et al. (15) describe how residents of Oakland, CA, United States avoid paying taxes by traveling to stores outside of the city to purchase SSBs at lower prices. Oakland is a medium-sized city, and authors suggest that cross-border shopping may be harder to deter in smaller tax locales. To mitigate cross-border shopping in Saudi Arabia, neighboring countries simultaneously implemented taxes on high-sugar foods or beverages (16). Tax avoidance by cross-border shopping likely weakens the effects of taxes by providing access to foods at cheaper prices in un-taxed locales and reducing the need to change behavior and substitute with more nutritious options.

##### Resistance from industry can present a barrier to both implementation and effects

Resistance from industry presented a barrier to implementation through stores failing to comply with details of new tax regimes. For example, stores in smaller localities did not always pass increases in price of taxed products on to their customers (21, 53, 58). These stores and SSB brands were concerned about competition from cross border shopping. Some stores preferred to incur the cost of the tax to avoid losing customers to stores outside the tax jurisdiction. Stores also did not always explicitly communicate the price change to customers. Powell and Leider (35) observes that while the Cook County, IL, United States tax ordinance required stores to display shelf prices inclusive of the total price with tax, retailers may not have complied. If retailers did not comply, consumers would not have known about the price change before check-out. In Chile, Caro et al. (58) found stores were not willing to incur the additional ‘menu’ costs required to physically change shelf prices to include the new tax. In this way vendor non-compliance could prevent the tax from affecting the prices of high-sugar foods or beverages within tax locales.

A few studies observed negative responses to taxes from producers (35, 41). Colchero et al. (41) report an increase in targeted advertising for SSB products in Mexico after implementation of the tax that may have influenced SSB purchases and consumption. This way of countering the effect of taxes on consumers may have presented a barrier to behavior change.

##### Low awareness of health risks associated with sugar-sweetened beverages and aerated beverages may present a barrier to behavior change

Low consumer awareness of the health risks associated with SSBs and high-sugar foods may have contributed to small changes in consumption post-implementation of taxes. Gonçalves and Pereira dos Santos (20) observed stockpiling of SSBs in the quarter before the tax was implemented and high rates of cross-border shopping. They conclude that SSB taxes in Portugal may not have produced the desired signaling effect to reduce consumption of high-sugar and medium-sugar products. In India, Law et al. (31) report that consumers may not have perceived the aerated beverage taxes as signaling health risks because the tax was rolled out as a part of general Goods and Services Taxes (GST), and not specifically as a health-related policy. Low awareness of health risks could prevent the tax from influencing consumption of foods and beverages that are high in sugar, fat or salt, reducing impact on diet and health outcomes.

#### Cost and sustainability information

None of the included studies conducted cost analysis. However, tax policies have the potential to be cost effective and sustainable because they generate revenue. Three studies report increases in revenue because of these taxes (20, 42, 55). While studies do not include information on how tax revenue was allocated within city, state or national budgets, there is potential for revenue to be invested in nutrition and health programming. Revenue from SSB taxes can be used to fund other health promoting activities, such as the Seattle SSB tax which supports nutrition, child health and education initiatives (59). The justification of these taxes as a revenue generating mechanisms should be considered separately from their justification as a public health tool. However, the signaling effect of these taxes may be diminished if revenue generation rather than health is seen as the goal.

Though some taxes, such as the Cook County, IL, United States taxes on SSBs (35) and the high-sugar food and beverage taxes in Norway (33) and Denmark (60) were repealed, nearly all taxes and subsidies were still implemented as of April 2022 (6). The costs of subsidy interventions were not reported.

## Discussion

We identified 49 impact evaluations related to fiscal policies to support a diverse diet. These represented 24 unique intervention-outcome-population combinations due to repeated evaluation of the same taxes. Most studies took place in high-income countries (*n* = 18). We assessed all 24 analyzed impact evaluations as having some concern or high risk of bias for at least two criteria. We assessed the two SRs with ‘high’ confidence, but Pfinder et al. (38) only included one evaluation. Common quality concerns were related to confounding and reporting bias. We did not observe publication bias. Taxes on SSBs reduced purchases of taxed beverages (Figure 5). The results were inconclusive on whether fiscal policies can meaningfully influence the availability and accessibility of targeted foods and beverages, diet quality, health and well-being outcomes. Although these policies are largely supported due to their assumed effect on diet and health, only seven studies evaluated diet or health outcomes. These outcomes are inherently more challenging to measure relative to purchasing outcomes. Positive effects of fiscal policies were facilitated by information to increase awareness on adverse health effects of SSBs or high-sugar foods and health benefits of subsidized foods, large geographic coverage, and the potential for revenue generation. Tax avoidance through cross-border shopping and vendor non-compliance may have prevented some taxes from achieving desired effects. In the following sections we summarize the effects of taxes, the effects of subsidies, and limitations in the evidence base.

### Effects of taxes on beverages and foods

Taxes on SSBs reduced purchases of taxed beverages with a standardized mean difference of −0.18 ([95%CI :−0.29 to −0.07], *n* = 15, Figure 5). Taxes had no impact on the overall volume of purchases of any beverage or the substitution of taxed beverages with untaxed (‘healthier’) beverages. Much of the reason for the apparent inconsistency in a reduction in taxed beverage purchases but no change in overall purchases is that there are different studies considering these different outcomes. The evidence base is too limited to determine if taxed beverages were substituted with untaxed substitute or complementary purchases. The available evidence is also too limited to determine if taxes on high-sugar foods and beverages impact consumption of taxed foods. Furthermore, it was not possible to make conclusions about the effects of these taxes on diet or health.

We observe mixed evidence for signaling effects of taxes. In many studies, consumers were affected by information or media campaigns. Without additional data on consumer choice, such as qualitative data on food decision-making or metrics of diet quality, we do not know if the effects were signaled by changes in prices of taxed goods or exposure to health information. The taxes may not influence consumers to substitute or purchase more nutritious untaxed beverages instead of taxed beverages or food.

### Effects of subsidies

With only four studies evaluating subsidy interventions, we do not have sufficient evidence to comment on their potential for impacting health and nutrition outcomes. However, these studies generally report null or small impacts on the selected diet quality and health measures evaluated.

### Limitations

This systematic review approach is limited by heterogeneity in pooled analyses, which may be explained in part by variation in outcomes used by study authors (e.g., volumes, grams of sugar, etc.). We conduct sensitivity analyses to determine if results varied by outcomes for purchases of taxed products and purchases of untaxed products and there were no differences. For diet and health outcome categories, we have presented effects individually in addition to presenting the combined analyses. The combined analyses are intended to provide additional information on the effects of these policies.

#### Gaps in the evidence base

Most included studies (*n* = 19) took place in HIC contexts, and only four studies evaluate subsidy interventions. Just two studies exclusively measured the impact of taxes on diet quality or health without focusing on purchasing outcomes, and only one study measured both purchasing and diet quality outcomes. Very few studies included sub-group analysis by socioeconomic status. Gibson et al. (48) explained that few stores in low-income neighborhoods participated in the study, and that common data sources do not include demographic information on consumers. Zhong et al. (62) observed that using purchase data limits generalizability of findings to relevant subpopulations, such as soda drinkers and low-income consumers. Because there are so few evaluations, there is insufficient evidence to be able to determine if key features of the taxes themselves, such as the size of the tax, the type of tax, or targeted beverages of foods, may affect the results.

#### High risk of bias

Nearly all studies used quasi-experimental designs, as the roll out of a fiscal policy would be challenging to evaluate using experimental methods. The most common evaluation designs were difference-in-differences (*n* = 11) and interrupted time series (*n* = 9) that relied on large panel or time series data. We assessed all studies in the evidence base to have concerns of bias, most often related to confounding, independence or contamination from other events or programs occurring simultaneously. High risks for confounding were common in difference-in-difference studies because authors did not include relevant time-varying characteristics in their model specifications (16, 20, 21, 26, 40). This may have been due to limited availability of data of seasonal consumption trends, socio-demographic and economic characteristics of purchasers, store characteristics and others. Nearly all difference-in-difference studies tested parallel trends and verified the core assumption of the method. For interrupted time series studies, there was limited discussion of independence of interventions from other factors which may have confounded the impact of the policies, such as information campaigns (34), or large overhauls of the whole tax system (31).

#### Reverse causation is not addressed

The theory of change justifying the adoption of fiscal policies assumes that changes in price influence purchasing behavior (Figure 1). However, given that nearly all included evaluations occur in democratic countries, it is possible that changes in perception of targeted foods precipitated adoption of a fiscal policy to influence consumption, for example pre-tax media campaigns to advocate implementing SSB taxes (35). While authors did not often explicitly mention potential for reverse causality in-text, we caution that constituents may have elected to tax or subsidize themselves in some contexts. A key determinant of implementing a fiscal policy could be changes in constituents’ attitudes toward calorie-dense foods and beverages that are high in sugar, fat or salt. As such, interrupted-time series, which assume that trends would have remained the same without the intervention and was commonly used in the included evaluations, may not be an appropriate evaluation approach for these policies.

#### Diet quality and health are not evaluated

These policies are largely supported due to their assumed effect on diet and health. However, only seven studies evaluated diet or health outcomes, likely because these outcomes are inherently more challenging to measure relative to purchasing outcomes. Nearly all studies (*n* = 17) used datasets that track consumer point-of-sale purchases at select stores such as Nielsen scanner data, Euromonitor, and Kantar World Panel. A strength of this method was that point-of-sale data avoided common measurement errors. Data was collected independent of the intervention and primary outcomes were assessed objectively. However, sales data do not directly measure changes in calories consumed, diet and health outcomes (17, 26, 51, 58, 61). These datasets rarely include household demographic information or even all the products subject to the tax. For example, Zhong et al. (62) reported that Euromonitor data did not have data on all the products included in the Philadelphia, PA, United States taxes. While point-of-sale data sources can illuminate changes in sales of products, purchases do not provide information on food and beverage intake, diet quality, or consumption patterns within households and neighborhoods. To elucidate the impact of fiscal policies on diet and health outcomes, Hernández-F et al. (32) used data from the Mexican government to connect Mexico’s SSB and sugary food taxes to dental health outcomes. Future research can similarly use population health data to investigate the causal links among fiscal policies, nutrition, and health outcomes.

#### Data sources not suitable to quantify impacts on selected outcomes

In many studies, the data sources used did not have key information needed to quantify impacts and adequately respond to their research questions. Authors report that they did not have requisite purchase data to measure changes in cross-border shopping (15, 35, 56) or purchases or volume sold in non-store venues such as restaurants, kiosks, or vending machines (17, 26, 41). Others could not measure intake of home-cooked foods, or perishable fresh foods such as fruits and meat and lacked household panel data (20, 43). Limited purchase data also prevented a few authors from testing for substitution effects. For instance, Falbe et al. (46) did not have data on purchases of non-SSBs, including diet soda, and could not analyze beverage substitution. Gibson et al. (48) attributed their inability to measure substitution effects to unavailability of data at the individual consumption level. Colchero et al. (43) report that they were not able to account for factors that may impact SSB sales independent of the tax in Mexico, such as temperature or advertising. Missing data on these purchases and consumption trends prevented authors from quantifying changes in their target outcomes.

### Implications for policy and practice

• The evidence base is too limited to make firm conclusions as to whether fiscal policies such as taxes and subsidies improve diet and health outcomes. Considering the widespread adoption of such policies, and the significant costs of subsidies in particular, it would be prudent to integrate rigorous research with the implementation of such policies. Only two studies considered the impacts of these taxes on diet quality.
  ⚬ The evidence suggests that beverage taxes reduce purchases of taxed beverages, but there is considerable heterogeneity in results.
  ⚬ The few studies evaluating subsidies suggest no or very small effects, but the evidence base is too small to draw firm conclusions.
• Taxes do not appear to generate substitution from calorie-dense beverages to relatively more nutritious beverages.
• Tax policymakers should consider conducting needs assessment to better understand health knowledge in their population. If appropriate, they may incorporate health information campaigns to amplify signaling effects of the taxes. This may improve adherence to the tax and reduce avoidance behaviors, such as cross-border shopping.
• Tax policies can trigger reformulation processes that can contribute to an anti-obesogenic food environment and a food systems transformation.
• Tax policies pay for themselves by generating revenue and may be more sustainable than other nutrition interventions. Revenue from taxes can be allocated toward nutrition and health programming.
• Integrating subsidies into existing food support systems may facilitate greater access among low-income populations.
• Because subsidies may require significant financial investment, additional research is needed to justify their implementation.
• Fiscal policies can be more rigorously evaluated if governments share routine monitoring data with researchers.

### Implications for research

• Anticipate limitations in data sources by developing rigorous evaluation design strategies that account for common sources of bias, such as confounding and independence.
  ⚬ Synthetic control analysis may be appropriate in these settings where there is a single intervention unit and non-intervention sites are likely to be fundamentally different than intervention sites (42, 14).
• Evaluations should use all available data and diversify data sources to better understand the impacts of SSB and high-sugar food taxes on diet and health outcomes.
  ⚬ Consider collecting new data or leveraging large scale data sources such those available through DHIS2, DQQ and the FAO (63–65). When using existing data sources, be sure to acknowledge any known limitations in their sampling procedures and data quality.
  ⚬ Consider partnering with government to access nutrition and health information.

• Prioritize evaluations in L&MIC contexts and evaluations of subsidies in all contexts, especially those focusing on nutritious foods, such as fruits, vegetables and pulses (2).
• To the extent practical, include equity aspects, such as subgroup analysis by socioeconomic status, body-mass index or pre-existing health conditions that may correlate with consumption of taxed or untaxed foods and beverages.
• Theory-based evaluations should prioritize measuring the effects of these interventions on health and wellbeing, rather than purchasing behavior.
• Mixed-methods evaluations could elucidate how consumers respond to these fiscal policies.
• Consider investigating variation in impacts based on the size and type of the tax or subsidy.
• From the perspective of policymakers, cost-evidence is needed to justify the use of subsidies, but may not be needed for the implementation of taxes, which generate revenue.
• Long-term outcomes should also be investigated as some consumer behaviors, such as purchases of diet cola, may change over time.

## Data availability statement

The raw data supporting the conclusions of this article will be made available by the authors, without undue reservation.

## Author contributions

CL and SS led the conception and design of the study and the meta-analysis. DA and TK led the search and screening, data extraction, and risk of bias analysis. MB contributed to the quantitative data analysis. VB served as research coordinator. JH performed the qualitative data analysis, served as publication manager, and wrote the first draft. All authors substantively contributed to the manuscript revision, read, and approved the submitted version.

## Footnotes

1 We define subsidies as interventions in which the government pays a *portion* of the price of a good. The evidence base on interventions which provide food, cash, or vouchers as subsidies is extensive. While these programs can all be conceptualized as reducing costs and increasing accessibility of food, the behavioral responses of consumers to the various delivery mechanisms are likely to be different. Therefore, we do not consider food vouchers, in-kind food provision, and cash transfers in our definition of subsidies.

2 Untaxed beverages varied by policy. Commonly, study authors include untaxed beverages such as water or diet versions of soda. Most study authors do not report differential effects of taxes on substitute or complementary untaxed beverages.
