# Corrigendum: Systematic review on fiscal policy interventions in nutrition

**DOI:** 10.3389/fnut.2024.1404372

**Published:** 2024-04-18

**Authors:** Jane Hammaker, Daniela Anda, Tomasz Kozakiewicz, Vinitha Bachina, Miriam Berretta, Shannon Shisler, Charlotte Lane

**Affiliations:** International Initiative for Impact Evaluation (3ie), Washington, DC, United States

**Keywords:** fiscal policies, nutrition, sugar-sweetened beverage consumption, taxes, subsidies

The authors made no mistakes; however, an article that was included in this review has been retracted since the review was published: *Changes in soft drinks purchased by British households associated with the UK soft drinks industry levy: controlled interrupted time series analysis*; BMJ (2021). The study was retracted due to significant methodological issues. The study discusses an intervention which is not the primary focus of our review (Hammaker et al., 2023), and was not included in any of our meta-analyses.

The retracted information does not change the scientific conclusions of our review in any way; however, readers should refer to the corrected citation to learn more about tiered taxes in the UK:

- Rogers NT, Pell D, Mytton OT, Penney TL, Briggs A, Cummins S, et al. Changes in soft drinks purchased by British households associated with the UK soft drinks industry levy: a controlled interrupted time series analysis. *BMJ Open*. (2023) 13:e077059. 10.1136/bmjopen-2023-077059

The authors apologize for this error and state that this does not change the scientific conclusions of the article in any way. The original article has been updated.

