# Supplementary material for: Systematic review on fiscal policy interventions in nutrition
Source: Front Nutr. 2022 Nov 29;9:967494. doi: 10.3389/fnut.2022.967494 (PMC9756132; doi:10.3389/fnut.2022.967494)
Supplement: Supplementary file 1 [file Table_1.DOCX]

## Supplement 0: Appendix Tables 1, 2 & 3

| **Appendix Table 1.** Summary of analyzed studies in the SR | | | | | | |
| --- | --- | --- | --- | --- | --- | --- |
| **First author** | **Year** | **Title** | **Region(s)** | **Study design** | **Intervention(s)** | **Impact(s)** |
| Aguilar | 2019 | The effectiveness of sin food taxes: evidence from Mexico | Mexico | Quasi-experimental | Beverages with added sugar were taxed at one peso per liter. Solid foods with a caloric density >274 kilocalories per 100 grams were also taxed at 8 percent of their pre-tax average price. | Calories purchased from taxed beverages and taxed foods decreased. The tax had no impact on untaxed beverages or food purchases. |
| Alsukait | 2020 | Evaluating Saudi Arabia's 50% carbonated drink excise tax: Changes in prices and volume sales | Saudi Arabia | Quasi-experimental | Carbonated beverages were taxed at 50 percent of their pre-tax price. Energy beverages and tobacco were taxed at 100 percent of their pre-tax price. | The tax decreased sales of taxed beverages. |
| Alvarado | 2019 | Assessing the impact of the Barbados sugar-sweetened beverage tax on beverage sales: an observational study | Barbados | Quasi-experimental | SSBs were taxed at 10 percent of their pre-tax price. | The tax had no impact on purchases of taxed SSBs or untaxed beverages. |
| Bleich | 2021 | Association of a sweetened beverage tax with purchases of beverages and high-sugar foods at independent stores in Philadelphia | Philadelphia, PA US | Quasi-experimental | Sugar and artificially sweetened beverages were taxed at 1.5 cents per fluid ounce. | Taxed SSBs purchased and calories consumed decreased, but the tax had no impact on purchases of untaxed beverages, high-sugar foods or calories consumed from high-sugar foods. |
| Cawley | 2020 | Oakland's sugar-sweetened beverage tax: Impacts on prices, purchases and consumption by adults and children | Oakland, CA US | Quasi-experimental | SSBs were taxed at 1 cent per ounce. | The tax had no impact on volume purchased of taxed and untaxed beverages, or consumption of added sugar for children or adults. |
| Cawley | 2020 | The impact of sugar-sweetened beverage taxes on purchases: evidence from four city-level taxes in the United States | Philadelphia, PA USA; San Francisco, CA US; Seattle, WA US; Oakland, CA US | Quasi-experimental | Philadelphia implemented a tax of 1.5 cents per ounce on SSBs and non-caloric sweetened beverages. Oakland implemented a tax of 1 cent per ounce on SSBs. San Francisco implemented a tax on SSBs of 1 cent per ounce. Seattle implemented a tax on SSBs of 1.75 cents per ounce. | Monthly purchases of taxed beverages decreased, but the tax had no impact on purchases of untaxed beverages. |
| Chakrabarti | 2016 | Effectiveness of food subsidies in raising healthy food consumption: public distribution of pulses In India | Andhra Pradesh, India; Himachal Pradesh, India; Punjab, India; Tamil Nadu, India | Quasi-experimental | Subsidies for pulses through the Public Distribution System. | Subsidies increased household consumption of pulses and daily intake of proteins |
| Chakrabarti | 2019 | Impact of subsidized fortified wheat on anaemia in pregnant Indian women. | Punjab, India; Tamil Nadu, India | Quasi-experimental | Subsidy for fortified wheat flour through Public Distribution System fair price shops. | The tax had no impact on hemoglobin levels of high-risk women in either state. |
| Colchero | 2016a | Beverage purchases from stores in Mexico under the excise tax on sugar sweetened beverages: observational study | Mexico | Quasi-experimental | Beverages with added sugar were taxed at 1 peso per liter. Solid foods with a caloric density >274 kilocalories per 100 grams were also taxed at 8 percent of their pre-tax average price. | The taxes had no impact on volume purchased of taxed and untaxed beverages. |
| Gonçalves | 2020 | Brown sugar, how come you taste so good? The impact of a soda tax on prices and consumption | Portugal | Quasi-experimental | SSBs with <80 grams of sugar per liter were taxed at €0.08 per liter. SSBs with >80 grams per liter were taxed at €0.16 per liter. | The taxes increased the quantity of liters sold for low sugar products, but had no impact on quantity of high, medium or zero sugar products sold. |
| Hernández-F | 2021 | Taxes to unhealthy food and beverages and oral health in Mexico: An observational study | Mexico | Quasi-experimental | Beverages with added sugar were taxed at one peso per liter. Solid foods with a caloric density >274 kilocalories per 100 grams were also taxed at 8 percent of their pre-tax average price. | The taxes had no impact on outpatient visits related to dental caries. |
| Howard | 2011 | Do school lunch subsidies change the dietary patterns of children from low-income households? | US | Quasi-experimental | Subsidy for school lunches for low-income students. | The subsidy had no impact on fruit consumption. |
| Kurz | 2021 | The causal impact of sugar taxes on soft drink sales: evidence from France and Hungary | France; Hungary | Quasi-experimental | In France, SSBs with <11g of sugar were taxed at €0.0755 eurocents per liter; >11g of sugar taxes were progressive and increased by €0.20 cents per liter as grams of sugar increased. In Hungary, SSBs containing syrup were taxed at 200 Forint per liter. Energy beverages containing methyl xanthine and taurine taxed at 250 Forint per liter. Energy beverages containing methyl xanthine were only taxed at 40 Forint per liter. Other soft beverages taxed at 7 Forint per liter. This tax also applied to the salt and caffeine content of pre-packaged foods. | In France, the tax had no impact on sales of taxed SSBs or any soft beverages.    In Hungary, the tax increased sales of taxed SSBs but had no impact on sales of all soft beverages. |
| Law | 2021 | Changes in take-home aerated soft drink purchases in urban India after the implementation of Goods and Services Tax (GST): An interrupted time series analysis | India | Quasi-experimental | Aerated beverages were taxed at 40 percent of their pre-tax price. | The taxes had no impact on volume purchased of taxed or untaxed aerated beverages |
| Nakamura | 2018 | Evaluating the 2014 sugar-sweetened beverage tax in Chile: An observational study in urban areas | Chile | Quasi-experimental | SSBs with >8g of sugar per liter were taxed at €0.12 per liter. SSBs with 5 to <8g of sugar per liter taxed at €0.08 per liter. | The tax decreased purchases of untaxed soft beverages for low and high-income groups, but had no impact for middle income groups. The tax decreased sugar purchases for all groups combined, as well as for high income and middle income participants, but did not reduce sugar consumption for low-income participants when isolated. |
| Øvrebø | 2020 | The effects of an abrupt increase in taxes on candy and soda in Norway: an observational study of retail sales | Norway | Quasi-experimental | High-sugar foods were taxed at 80 percent and beverages at 40 percent of their pre-tax prices. | The taxes had no impact on sales of taxed candy or beverages. |
| Øvrum | 2013 | Evaluating free school fruit: results from a natural experiment in Norway with representative data | Norway | Experimental | Subsidies for fruits and vegetables for school children at 3.5 NOK per day. | The subsidies increased consumption of fruits and vegetables. |
| Pell | 2020 | Anticipatory changes in British household purchases of soft drinks associated with the announcement of the Soft Drinks Industry Levy: A controlled interrupted time series analysis | UK | Quasi-experimental | SSBs with 8g of sugar per 100 ml were taxed at £0.24 per liter. Beverages with 5 to <8 g of sugar per 100 ml were taxed at £0.18 per liter. | The tax decreased purchases of low-tax beverages and untaxed beverages, but had no impact on purchases of high-tax beverages. |
| Pfinder | 2020 | Taxation of unprocessed sugar or sugar-added foods for reducing their consumption and preventing obesity or other adverse health outcomes | Hungary | Systematic review | Taxes on foods high in sugar, salt and caffeine, including SSBs. | The tax decreased the consumption of taxed sugar-added foods. This effect is based on one included impact evaluation only. |
| Powell | 2020 | Evaluation of changes in beverage prices and volume sold following the implementation and repeal of a sweetened beverage tax in Cook County, Illinois | Cook County, IL USA | Quasi-experimental | SSBs and ASBs taxed at 1 cent per fluid ounce. There was a pre-tax media campaign on revenue generation and later on health benefits. | The tax had no impact on purchases of taxed or untaxed beverages, even after the tax was repealed. |
| Powell | 2021 | Evaluation of changes in grams of sugar sold after the implementation of the Seattle sweetened beverage tax | Seattle, WA US | Quasi-experimental | SSBs with at least 40 kcal per 12 ounces were taxed at 1.75 cents per ounce. | The tax had no impact on grams of sugar sold from taxed beverages, untaxed beverages, sweets, or stand-alone sugar. |
| Puig-Codina | 2020 | The impact of taxing sugar-sweetened beverages on cola purchasing in Catalonia: an approach to causal inference with time series cross-sectional data | Catalonia, Spain | Quasi-experimental | SSBs with >8g of sugar per liter were taxed at €0.12 per liter. SSBs with 5 to <8g of sugar per liter taxed at €0.08 per liter. | The tax increased purchases of diet cola and decreased purchases of regular cola. |
| Rojas | 2021 | Do taxes on soda and sugary drinks work? Scanner data evidence from Berkeley and Washington state | Berkeley, CA US & Seattle, WA US | Quasi-experimental | In Berkeley, SSBs taxed at 1 cent per ounce.  In Seattle, SSBs with at least 40 kcal per 12 ounces taxed at 1.75 cents per ounce. | The tax decreased purchases of taxed beverages in both Berkeley and Seattle. |
| Royo-Bordonada et al. | 2019 | Impact of an excise tax on the consumption of sugar-sweetened beverages in young people living in poorer neighbourhoods of Catalonia, Spain: a difference in differences study | Catalonia, Spain | Quasi-experimental | SSBs with >8g of sugar per liter were taxed at €0.12 per liter. SSBs with 5 to <8g of sugar per liter taxed at €0.08 per liter. | The tax had no impact on consumption of taxed or untaxed beverages. |
| Silver | 2017 | Changes in prices, sales, consumer spending, and beverage consumption one year after a tax on sugar-sweetened beverages in Berkeley, California, US: A before-and-after study | Berkeley, US | Quasi-experimental | SSBs taxed at 1 cent per ounce. | The tax decreased sales of taxed and untaxed beverages. |
| Teng | 2019 | Impact of sugar‐sweetened beverage taxes on purchases and dietary intake: Systematic review and meta‐analysis | US; Chile; France; Mexico; Spain | Systematic review and meta-analysis | Equivalent of a 10 percent SSB tax on pre-tax prices. | Taxes decreased consumption (a combined measure of purchases and dietary intake) of taxed beverages, largely weighted to results from France. No impact on the same measure was found for total untaxed beverage consumption though increases were found in three of the four jurisdictions (Berkeley, CA USA, Mexico, and other United States). |

| **Appendix Table 2.** Summary of linked studies reviewing included interventions and outcomes | | | | | | | | | |
| --- | --- | --- | --- | --- | --- | --- | --- | --- | --- |
| **First author** | **Year** | **Title** | **Region(s)** | **Evaluation method** | **Intervention** | **Outcomes** | **Barriers and facilitators to implementation** | **Sources of bias and limitations** | **Brief description of why analyzed study was selected** |
| Alvarado | 2021 | Evidence of a health risk 'signaling effect' following the introduction of a sugar-sweetened beverage tax | Barbados | Interrupted Time Series | Tax | Weekly SSB sales | None reported | This study faced several limitations related to data availability. First, given the short timeframe between the announcement of the tax and implementation (three months), no baseline data on perceptions of different SSBs were collected. Instead, the authors relied on interview data collected 20–25 months after the implementation of the tax, limiting their ability to assess whether perceptions changed over time. | Study period - the selected study collected data over a longer time period or is a more recent analysis of the same intervention and data |
| Bleich | 2020 | The association of a sweetened beverage tax with changes In beverage prices and purchases at independent stores | Philadelphia, PA USA | Difference-in-difference | Tax | Purchased fluid ounces of taxed and non-taxed beverages; total calories from high-sugar food and SSB purchases | The authors report that they did not collect data at stores in neighboring untaxed communities to assess tax avoidance, a potential barrier to implementation of the tax. | Unobserved factors might have influenced socio-demographic characteristics of Philadelphia and Baltimore over the study period, causing time-dependent residual confounding. | Study period - the selected study collected data over a longer time period |
| Capacci | 2019 | The impact of the French soda tax on prices and purchases. An ex post evaluation | France | Difference-in-difference | Tax | Average weekly per capita purchase of soft drinks; average weekly per capita purchase of pure fruit juices; average weekly per capita purchase of water | None reported | There was limited availability of relevant socio-demographic information in the dataset and the use of additional covariates (e.g. income, better geocoding, education levels, etc.) would enable more powerful identification strategies. The study data only covers drinks purchased for home but out-of-home consumption behavior is likely to be very relevant to assess the ultimate weight or health impact of the tax. Study data do not allow inference on the longer term effects of the tax, and even with a longer time series it would become difficult to assume that the DID model can isolate the tax effect from other confounding factors intervening in the four regions. | Internal validity - the selected study used more internal validity checks |
| Caro | 2018 | Chile’s 2014 sugar-sweetened beverage tax and changes in prices and purchases of sugar sweetened beverages: An observational study in an urban environment | Chile | Fixed Effects | Tax | Household average monthly purchased volume of untaxed beverages; Household average monthly purchased volume of taxed 10% beverages (low-SSBs); Household average monthly purchased volume of taxed 18% beverages (high-SSBs); Average monthly purchased calories of untaxed beverages; Average monthly purchased calories of taxed 18% High-SSBs | Authors observe that stores were hesitant to change store prices and incur administrative costs, which might have prevented some brands from increasing prices, especially considering the relatively small size of the tax hike. | Inability to assess a causal relationship between the tax modifications and changes in prices or purchases due to the potential presence of other simultaneous trends affecting underlying preferences and due to the inability of household food purchase data to capture all beverages consumed (particularly those consumed out of the home). The sample is also more likely to represent urban and older households and therefore does not entirely reflect changes in purchases among younger households and the rural population. | Number of controls - the selected study used more controls |
| Castelló | 2020 | Impact of SSB taxes on sales | Catalonia, Spain | Interrupted Time Series | Tax | Total purchased liters SSBs; Total purchased liters zero light (non-taxed) drinks | The study only evaluates the short-run effect of the policy (three months of post-reform data). | The data come from a supermarket chain that is accessible to the entire territory, but only represents 10% of the total Catalan market and may not be representative of the population. | Study design -the selected study used a more rigorous analytical method |
| Cawley | 2018 | The impact of the Philadelphia beverage tax on purchases and consumption by adults and children | Philadelphia, PA USA | Difference-in-difference | Tax | Beverage purchases: volume in ounces ; grams of added sugar consumption from SSBs per day; monthly frequency of consumption of taxed beverages; monthly frequency of consumption of untaxed beverages | None reported | There are a small number of clusters, with only two time periods and a comparison of Philadelphia to comparison communities in the same MSA. The survey data is also self-reported, which may contain some degree of reporting error. To the extent the tax affected the extent of reporting error in Philadelphia relative to the nearby comparison communities, such a change may bias their estimates of the impact of the tax on consumption. | Relevant outcomes - the selected study used more biologically relevant outcomes |
| Colchero | 2016b | Beverages Sales in Mexico before and after Implementation of a Sugar Sweetened Beverage Tax | Mexico | Quasi-experimental | Tax | Sales of sugar-sweetened beverages and plain water (liters/capita) | None reported | Authors report they were not able to control for factors that may influence beverage sales independent of the tax, such as temperature or advertising. However, the model accounts for economic activity and population size. | Study design -the selected study used a more rigorous analytical method |
| Colchero | 2017 | After Mexico implemented a tax, purchases of sugar-sweetened beverages decreased and water increased: Difference by place of residence, household composition, and income level | Mexico | 2-part model | Tax | Purchases of SSBs; Purchases of bottled water; Purchases of SSBs; Purchases of bottled water | None reported | The authors recognize that their data source did not allow them to differentiate between taxed and untaxed beverages. The SSB category includes untaxed beverages such as sodas or flavored waters with artificial sweeteners. However, untaxed beverages in the SSB category represented only 6% of total beverages sales, and there were no substantial increases after 2014. As discussed elsewhere, beverage purchases may have been underestimated, but there is no reason to believe that underreporting changed across rounds. In addition, the consumption of potable water or any homemade beverage was not reported. The authors also acknowledge that in the absence of an experimental design they used cross-sectional surveys, and that their results should be interpreted as associations rather than causal effects. | Relevant outcomes - the selected study used more biologically relevant outcomes |
| Debnam | 2015 | Selection effects and heterogeneous demand responses to the Berkeley Soda Tax Vote | Berkeley, CA USA | Interrupted Time Series | Tax | SSB purchases: % change in volume purchased | None reported | Berkeley is not representative of the US population as a whole. | Study design -the selected study used a more rigorous analytical method |
| Edmondson | 2021 | Association of a sweetened beverage tax with soda consumption in high school students | Philadelphia, PA USA | Difference-in-difference | Tax | Weekly servings of soda (taxed beverage) consumed; weekly servings of 100% juice (nontaxed beverages) consumed; Weekly servings of 100% milk (nontaxed beverages) consumed | None reported | Estimations rely on self report data which is prone to measurement error and recall bias. The 100% juice and milk products were not parallel trended in pre tax period, and the survey only accounts for only soda, 100% juice and milk, leaving out of the scope consumption of other sweetened beverages. The survey may have also underestimated the tax response in Black adolescents given that the study is limited in relevant covariates. Authors did not have information on socioeconomic status or differential fixed effects for cities. | Relevant outcomes - the selected study used more biologically relevant outcomes |
| Falbe | 2016 | Impact of the Berkeley excise tax on sugar-sweetened beverage consumption | Berkeley, CA USA | Difference-in-difference | Tax | SSB consumption | Berkeley is a single city of relatively high socioeconomic status, and results may not generalize to other cities. The study did not assess a comprehensive list of non-SSBs, including diet soda, so it was not possible to examine beverage substitution beyond water. The authors are unaware of concurrent interventions in Berkeley during this time period, and the increase in SSB consumption in comparison cities suggests that external factors may have encouraged higher consumption in the Bay Area. This area also experienced higher-than-average temperatures in the relevant months of 2015 as compared to 2014. The post tax consumption data is less than 6 months after implementation and reflects short-term impacts of the tax. | The authors mention the possibility that their samples were not independent, so their analysis likely overestimated standard errors for pre- versus post tax change (and hence understated statistical significance). They also did not collect measures of self-reported height, weight, or desire to lose or maintain weight, which may have been associated with magnitude of change in SSB consumption in response to the tax. The self-reported behaviors are vulnerable to social desirability bias. | Study period - the selected study collected data over a longer time period or is a more recent analysis of the same intervention and data |
| Fichera | 2021 | How do consumers respond to "sin taxes"? New evidence from a tax on sugary drinks | Catalonia, Spain | Difference-in-difference | Tax | Natural log of sugar purchased via SSBs | The tax was at the state and not national level. | The households may have changed their shopping behavior in anticipation of the tax by, for example, building up a stock of SSBs. The authors only have time-invariant socio-economic characteristics. The results reflect the behavior of households shopping at one supermarket chain which may differ from other households. Information is available is only for sugar content at one point in time, so authors could not test whether product reformulation had occurred. The authors only have data from loyalty card customers who could behave differently from other customers. | Study design -the selected study used a more rigorous analytical method |
| Gibson | 2021 | No evidence of food or alcohol substitution in response to a sweetened beverage tax | Philadelphia, PA USA | Difference-in-difference | Tax | Volume sales of candy; Volume sales of sweet snacks; Volume sales of salty snacks; Volume sales of beverage concentrates; Volume sales of other foods; Volume sales of alcohol per store - wine; Volume sales of alcohol per store - spirit | The authors suggest that effects of that tax on SSBs may be larger in Philadelphia because it is the poorest of the 10 largest U.S. cities, and the only U.S. jurisdiction that also taxes artificially-sweetened beverages, limiting consumers’ options to substitute with other sweet beverages. In addition, evaluations of the Philadelphia tax have not observed increases in nontaxed beverage sales, suggesting people might be spending that money on other products. | This study has several limitations, including only examining data at chain retail stores and not other retailers like independent stores and restaurants. Second, restrictive and changing liquor laws in Pennsylvania precluded examining substitution to beer and confounded wine analyses because beer and wine were not consistently available for sale in food stores. Third, changes in sales were at the store level and not the individual level, so testing for substitution effects among individuals who reduced their taxed beverage purchasing was not possible. Fourth, these data were at the subcategory level (e.g., potato chips), not the individual product level. Therefore, understanding whether people are purchasing more, but cheaper, food items, or stores are lowering prices, cannot be tested. Finally, the very small number of stores in low-income neighborhoods prevented analyses by neighborhood income level. | Relevant outcomes - the selected study used more biologically relevant outcomes |
| Lawman | 2020 | One-year changes in sugar-sweetened beverage consumers’ purchases following implementation of a beverage tax: a longitudinal quasi-experiment | Philadelphia, PA USA | Difference-in-difference | Tax | Purchased ounces of taxed beverages; purchased ounces of nontaxed beverages; Total food and beverage spending ($); Percent spent on taxed beverages (%) | None reported | The study used only 2 weeks of receipts per time point, which may not capture all purchasing behavior. The use of a run-in period may limit generalizability because noncompliant respondents were not enrolled. It is also possible there were receipts that weren't submitted. The study may be underpowered to capture heterogenous outcomes for daily or twice daily consumers, or those living further from the border. The small number of groups may underestimate DiD effect sizes. | Relevant outcomes - the selected study used more biologically relevant outcomes |
| Lee | 2019 | Sugar-sweetened beverage consumption 3 years after the Berkeley, California, sugar-sweetened beverage tax | Berkeley, CA USA | Difference-in-difference | Tax | SSB consumption times/day | None reported | There is a risk of limited generalizability and unmeasured confounding. Berkeley, a small and highly educated city, may not translate to other geographic areas. | Study design -the selected study used a more rigorous analytical method |
| Leger | 2021 | The impact of the Oakland SSB tax on prices and volume sold: A study of intended and unintended consequences | Oakland, CA USA | Difference-in-difference | Tax | Beverage volume sold (oz) of all taxed SSBs; Beverage volume sold (oz) of all -nontaxed beverages | None reported | None reported | Study period - the selected study collected data over a longer time period or is a more recent analysis of the same intervention and data |
| Leider | 2022 | Longer-term impacts of the Oakland, California, sugar-sweetened beverage tax on prices and volume sold at two-years post-tax | Oakland, CA USA | Difference-in-difference | Tax | Beverage volume sold (oz) of all taxed SSBs; Beverage volume sold (oz) of all -nontaxed beverages | Differences in local geography and commuting patterns will likely play an important role in the extent of cross-border shopping in response to a local tax, and policymakers should assess the potential for cross-border shopping in their jurisdiction when planning a new tax. | Data is limited to food stores and does not cover other venues such as restaurants and vending machines which are important sources of SSBs and untaxed beverages. Also, data was aggregated to the site level to protect store confidentiality, so authors were unable to examine differences by store type or assess the representativeness of the data to all food stores. | Relevant outcomes - the selected study used more biologically relevant outcomes |
| Pedraza | 2019 | The caloric and sugar content of beverages purchased at different store-types changed after the sugary drinks taxation in Mexico | Mexico | Fixed Effects | Tax | Predicted mean volume, kilocalories and total sugar of taxed and untaxed beverages purchased by of Nielsen CPS households (per capita/day) from 2012-2016 | Industry reformulation of products facilitated the implementation. The study also mentioned that it is essential that the SSBs tax is accompanied by a set of policy actions including unhealthy food and beverage marketing restrictions, the implementation of a clear front-of pack labeling system, and norms for healthy food and beverages in schools, to shift the food supply and further reduce purchases of SSBs. | The authors used the same nutritional profile for a given product across the entire time-period (the post tax profile) so they do not reflect any reformulation (e.g., reductions in sugar to avoid the tax) that may have occurred. The Nielsen CPS only captures products with barcodes purchased at retail food outlets. Thus, home-prepared drinks with sugar (i.e. aguas frescas), concentrates normal bar-coded containers of taxed beverages bought at restaurants, and sugary drinks purchased from street vendors were not captured. Nielsen CPS collects purchasing data and not intake. | Study design -the selected study used a more rigorous analytical method |
| Pell | 2021 | Changes in soft drinks purchased by British households associated with the UK soft drinks industry levy: controlled interrupted time series analysis | United Kingdom | Interrupted Time Series | Tax | Absolute change in sugar purchased for "high-tier" and "low-tier" taxed SSBs | None reported | Although KWP gathers data on other purchases, this smaller panel was developed in mid-2015 and was therefore unsuitable for our studies due to the inability to estimate strong pre-announcement trends. The KWP data is gathered at the home level and does not account for waste or unequal sharing between households. Nonetheless, the statistics provide a fair consumption estimate. Apart from soft drinks, alcohol, and confectionary, the authors didn't look at any other categories. In interrupted time series investigations, the estimation of effect magnitude is predicated on a modelled counterfactual that may be erroneous. | Relevant outcomes - the selected study used more biologically relevant outcomes |
| Powell | 2021 | Impact of a sugar-sweetened beverage tax two-year post-tax implementation in Seattle, Washington, United States | Seattle, WA USA | Difference-in-difference | Tax | Volume taxed beverages sold in percentage difference in volume; Volume untaxed beverages sold in percentage difference in volume | None reported | The scanner data have limitations due to confidentiality and restrictions issues, so information on all products was not present. The assessment does not measure the extent of tax pass-through. Also, the assessment does not measure the representativeness of the full food store retail sector. The results from this study do not cover prices or volume sold in non-store venues. Results from Seattle may not generalize to other local tax jurisdictions, highlighting the need for additional longer-run studies. | Relevant outcomes - the selected study used more biologically relevant outcomes |
| Powell | 2020 | The impact of a sweetened beverage tax on beverage volume sold in Cook County, Illinois and its border area | Cook County, IL USA | Difference-in-difference | Tax | Volume sold in percentage difference in volume | Authors were unable to determine how the volume of goods sold differed depending on the distance from the tax jurisdiction border but suspect this may have influenced the effects of the tax. | Although they covered a large market, they were unable to stratify their results by store type. Retail shops closer to the taxing border have proved to have reduced tax pass-through. As a result, they would predict a reduced impact on sales in such stores. | Study period - the selected study collected data over a longer time period |
| Roberto | 2019 | Association of a beverage tax on sugar-sweetened and artificially sweetened beverages with changes in beverage prices and sales at chain retailers in a large urban setting | Philadelphia, PA USA | Difference-in-difference | Tax | Taxed beverage volume sales in millions, oz; Untaxed beverage volume sales in millions, oz; Food and beverage sales in thousands, $; Taxed beverage volume sales in millions, oz; Untaxed beverage volume sales in millions, oz; Food and beverage sales in thousands, $ | None reported | This study has several limitations. First, the data only included beverages sold at chain retailers. The study did not include data from New Jersey, where some cross-border shopping may have occurred. Third, the data did not include overall store revenue. Fourth, this study did not report on changes in beverage consumption or health outcomes associated with the tax. | Relevant outcomes - the selected study used more biologically relevant outcomes |
| Schmacker | 2020 | Do prices and purchases respond similarly to soft drink tax increases and cuts? | Denmark | 2-part model | Tax | Quantity of high taxed soft drinks purchased; Quantity of low taxed soft drinks purchased; Quantity of untaxed non-sugary beverages purchased; Quantity of untaxed sugary beverages purchased; Quantity of Milk purchased | None reported | There was no natural control group available since the tax variation is on a national scale. | Study design -the selected study used a more rigorous analytical method |
| Seiler | 2021 | The impact of soda taxes: Pass-through, tax avoidance, and nutritional effects | Philadelphia, PA USA | Difference-in-difference | Tax | Quantity sold (measured in ounces) of taxed beverages; quantity sold (measured in ounces) of untaxed beverages; total number of calories sold via beverage sales; total grams of sugar sold via beverage sales | Authors observe that the tax was not effective in generating revenue because consumers substituted to stores outside of Philadelphia. For taxes that are localized, high tax rates will be sub-optimal for generating revenue because they lead to cross-shopping, which reduces the tax base. Furthermore, altering the tax base along the geographic or product dimension will likely have a different impact on tax revenue and nutritional intake. A larger geographic coverage will make cross-shopping more difficult and therefore generate greater tax revenue. A narrower product coverage instead will improve nutritional intake at the expense of lower tax revenue. | None reported | Relevant outcomes - the selected study used more biologically relevant outcomes |
| Taillie | 2017 | Do high vs. low purchasers respond differently to a nonessential energy-dense food tax? Two-year evaluation of Mexico's 8% nonessential food tax | Mexico | Fixed Effects | Tax | Percent of food purchases that is taxed | None reported | None reported | Relevant outcomes - the selected study used more biologically relevant outcomes |
| Zhong | 2020 | Sugar-sweetened and diet beverage consumption in Philadelphia one year after the beverage tax | Philadelphia, PA USA | Difference-in-difference | Tax | Beverage consumption; Frequency | None reported | The sample size was relatively small, and due to the large variance in consumption data, this study was only powered to detect large changes relative to the baseline consumption. These findings may be applicable to other large US cities with demographically varied populations, but they are unlikely to be applicable to subpopulations such as soda drinkers and low-income inhabitants. With only one pre-tax time period, the authors were unable to test the parallel trends assumption of the difference-in-differences method (which assumes the average outcomes for treated and control groups would have followed parallel paths over time). Self-reported consumption of beverages likely has measurement error. | Relevant outcomes - the selected study used more biologically relevant outcomes |

| **Appendix Table 3.** Extended summary of analyzed studies in the SR | | | | | | | | | | | | | | | |
| --- | --- | --- | --- | --- | --- | --- | --- | --- | --- | --- | --- | --- | --- | --- | --- |
| **First author** | **Year** | **Region(s)** | **Fiscal policy** | **Detailed intervention** | **Collaborating organization** | **Evaluation or synthesis method** | **Hypothesized mechanisms of action** | **Covariates considered** | **Impacts** | **Barriers and facilitators to implementation** | **Equity consideration** | **Sources of bias and limitations** | **Risk of bias** | **Conclusions** | **Effective?** |
| Aguilar | 2019 | Mexico | Tax | Beverages with added sugar were taxed at one peso per liter. Solid foods with a caloric density >274 kilocalories per 100 grams were also taxed at 8 percent of their pre-tax average price. | Instituto Tecnológico Autónomo de México | Interrupted time series | Taxes on beverages with added sugar and high caloric dense foods will reduce obesity. | Household fixed effects, seasonality with 51 week dummies, and dummies for product groups | Calories purchased from taxed beverages and taxed foods decreased. The tax had no impact on untaxed beverages or food purchases. | None reported | Though no sub-group analysis was conducted, authors report that products considered part of the *canasta basic* (basic consumption basket), like oil, milk, bread, among others, were exempt from the tax to limit impact on lower income segments of the population. | Authors report limitations in sources of data. The data measures purchases, which do not necessarily correspond to consumption, and only includes information of processed packaged foods - it excludes fresh foods like fruits and meat. Consumption data limited to KWP records of in-home consumption. | **Some concerns identified** | Taxes on SSBs and high-sugar foods decreased purchases, but it is unlikely that taxes improve diet quality or health. | Yes |
| Alsukait | 2020 | Saudi Arabia | Tax | Carbonated beverages were taxed at 50 percent of their pre-tax price. Energy beverages and tobacco were taxed at 100 percent of their pre-tax price. | Friedman School of Nutrition Science and Policy, Tufts University | Difference-in-differences | The tax increases prices of beverages, decreases beverage sales, decreases beverage consumption, and increases sale of bottled water. | None reported | The tax decreased sales of taxed beverages. | Authors report adherence to the tax was facilitated by limited opportunities for cross-border shopping because neighboring countries implemented similar SSB taxes, and pre-tax ban on the sale of alcohol. | None reported | Authors report several limitations including 1) using annual volume sales data for 2017, when the tax was only partially implemented; 2) limited information on prices for several brands; 3) possible heterogeneity in pass-through changes in beverage prices; and 4) using volume sale data, which may not capture changes in beverage consumption | **High risk of bias** | Taxes on carbonated beverages decreased purchases; authors do not report impact on untaxed beverages such as bottled water, so we cannot conclude that taxes improve diet quality or health. Limited opportunities for cross-border shopping may have facilitated changes in purchases of taxed beverages. | Yes |
| Alvarado | 2019 | Barbados | Tax | SSBs were taxed at 10 percent of their pre-tax price. | Canadian International Development Research Centre and the US Center for Disease Control and Prevention | Fixed-effects models | The tax increases prices of SSBs and decreases consumption. | Linear time trend, month indicators,  monthly tourist arrivals, monthly consumer price index, lag of residual | The tax had no impact on purchases of taxed SSBs or untaxed beverages. | None reported | None reported | Authors report that data may not be representative of all SSB sales and the findings from these data are limited to purchasing behaviors amongst the subset of people who shop at included chains. Because the authors used sales data, they were not able to report purchases in mL per person, and are less comparable with other studies. | **Some concerns identified** | Purchasing behavior did not change as a result of the tax, so it is unlikely that diet or health outcomes changed either. | No |
| Bleich | 2021 | Barbados | Tax | Sugar and artificially sweetened beverages were taxed at 1.5 cents per fluid ounce. | Bloomberg Philanthropies | Difference-in-differences | The authors hypothesized the tax would be associated with significant beverage price increases and reductions in taxed beverage sales, with limited substitution to high-sugar foods. Their secondary aims examined differences by beverage sweetener status and container size, as well as neighborhood income and customer education level. | Gender, race, ethnicity, education, who the purchase was for, frequency visiting the store, city residency, and total reported spending | Taxed beverages purchased and calories consumed from SSBs decreased, but the tax had no impact on purchases of untaxed beverages, high-sugar foods or calories consumed from high-sugar foods. | None reported | Sub-group analyses were conducted for education completed and neighborhood income levels | None reported | **High risk of bias** | Taxes on SSBs and high-sugar foods decreased purchases of SSBs, but had no impact on purchases of high-sugar foods. It is unlikely that taxes improve diet quality or health. | Yes, for SSBs, No for high-sugar foods |
| Cawley | 2020 | Oakland, CA USA | Tax | SSBs were taxed at 1 cent per ounce. | Cornell University, University of Iowa, Mathematica, NBER, Robert Wood Johnson Foundation | Difference-in-differences | Taxes on SSBs will increase retail prices of SSBs for consumers, reduce SSB purchases and reduce SSB consumption. There may be spillover effects that reduce SSB consumption for people who live outside of the tax jurisdiction. | Household fixed effects, month fixed effects | Monthly purchases of taxed beverages decreased, but the tax had no impact on purchases of untaxed beverages | None reported | None reported | The authors report relatively small sample sizes for Oakland, San Francisco and Seattle compared to Philadelphia, and cannot isolate the effect of the tax by city. | **Some concerns identified** | Taxes on SSBs decreased purchases of SSBs, and results were consistent in four major large U.S. cities. However, taxes had no impact on purchases of untaxed beverages. It is unlikely that taxes improve diet quality or health. | Yes |
| Cawley | 2020 | Philadelphia, PA USA; San Francisco, CA USA; Seattle, WA USA; Oakland, CA USA | Tax | Philadelphia implemented a tax of 1.5 cents per ounce on SSBs and non-caloric sweetened beverages. Oakland implemented a tax of 1 cent per ounce on SSBs. San Francisco implemented a tax on SSBs of 1 cent per ounce. Seattle implemented a tax on SSBs of 1.75 cents per ounce. | Cornell University, University of Iowa, Mathematica, NBER, Robert Wood Johnson Foundation | Difference-in-differences | SSB tax results in higher retail prices for consumers, reduced purchases of SSBs and reduced consumption, but possibility of spillover effects | Age, gender, Black, Hispanic, household size, FPL, Oakland residency, indicators for days of the week, time of day, day of the study, interview location, whether the interview occurred after implementation of the tax, and store-type | The tax had no impact on volume purchased of taxed and untaxed beverages, or consumption of added sugar for children or adults | The authors report potential for cross border shopping, in which residents of Oakland evade the tax by purchasing SSBs at stores outside of the city. | Authors conduct subgroup or differential analysis by race, socioeconomic status, and age (adult/child) | Small sample sizes, and potential for reporting bias. | **High risk of bias** | Purchasing behavior did not change as a result of the tax, so it is unlikely that diet or health outcomes changed either. Cross-border shopping may have counteracted impacts of the tax. No impacts on consumption of added sugar were observed for adults or for children. | No |
| Chakrabarti | 2019 | Punjab, India; Tamil Nadu, India | Subsidy | Subsidy for fortified wheat flour through Public Distribution System fair price shops. | International Food Policy Research Institute | Difference-in-differences | Wheat flour that is fortified and sold through public distribution fair price shops will reduce incidence of anemia in pregnant (high-risk) women. | Years of schooling, number of IFA tablets consumed, age, and dummy variables for households that cook with wood, women who were married before 18 years of age, urban households. religion of household (Hindu or Muslim), caste category of the household (scheduled caste, scheduled tribe, or other backward classes), and household wealth quintiles. | The subsidy had no impact on hemoglobin levels of high-risk women in either state. | Authors report that a 2016 publication of guidelines on safe and effective levels of iron, folic acid, and vitamin B12 in wheat may have facilitated implementation. However, the Public Distribution System (PDS) may not be the right channel to distribute subsidized fortified food because of low proportion of purchases and low consumer demand for fortified wheat. | Intervention targets women with high risk of anemia | Delayed data collection (nearly 10 years) could render estimates susceptible to time varying confounders. Improvements in PDS, the public health system, and/or other social assistance programs may have also impacted estimates. Correlation between state‐level changes and introduction of flour fortification could bias wheat fortification estimates. | **High risk of bias** | Subsidy for fortified wheat did not increase hemoglobin levels of high-risk women because wheat consumption was low across the population. It is unlikely that the subsidy improved diet quality or health. | No |
| Chakrabarti | 2016 | Andhra Pradesh, India; Himachal Pradesh, India; Punjab, India; Tamil Nadu, India | Subsidy | Subsidies for pulses through the Public Distribution System | Indian Council of Agricultural Research (ICAR) and the Swiss Agency for Development | Fixed effects model | Introducing pulses into India's PDS will improve nutritional outcomes of the beneficiaries. | Location of the household (rural or urban), main occupation of the family, family size and sex, age, religion, caste group, education level of household head, whether the household purchased rice, wheat, or sugar from the PDS, household source of cooking and lighting fuel, whether any member consumed meals outside of the home and land quintiles | Subsidies increased household consumption of pulses and daily intake of proteins in 4 states | Introducing pulses into PDS made it somewhat easier for households to access and use the PDS. | Subgroup analysis for poor households | None reported | **High risk of bias** | Subsidies for pulses increased household consumption and daily protein intake; authors do not report health impacts. Introducing the subsidies through the Public Distribution System (PDS) facilitated access in four states. | Yes |
| Colchero | 2016 | Mexico | Tax | Beverages with added sugar were taxed at 1 peso per liter. Solid foods with a caloric density >274 kilocalories per 100 grams were also taxed at 8 percent of their pre-tax average price. | Bloomberg Philanthropies; the Robert Wood Johnson Foundation; Instituto Nacional de Salud Pública; Carolina Population Center | Fixed effects model | Tax on SSBs may reduce SSB purchases and increase purchases of untaxed beverages. | Socioeconomic status, age, sex and household characteristics | The taxes had no impact on volume purchased of taxed and untaxed beverages. | Barriers to implementation of the tax included increased advertising of sugar-sweetened beverages and differential pass-through rates of the tax by brand and beverage size, which resulted in consumers shifting to lower-priced versions of taxed carbonated beverages | Sub-group analysis by socio-economic status | Authors report several limitations of their data source. The data only represent consumers in Mexican cities with more than 50,000 residents, and cannot quantify changes in calories and other nutrients purchased. No data on dietary intake or purchases of taxed beverages outside of selected stores was available. The data only included information on taxed beverages, and excluded non-essential energy dense foods that were also subject to the tax. | **High risk of bias** | Taxes on SSBs and high-sugar foods did not change consumer behavior, so it is unlikely that diet quality or health changed either. Lower-priced versions of taxed beverages were available due to differential tax pass-through rates, which may have counteracted the effect of the taxes. | No |
| Gonçalves | 2020 | Portugal | Tax | SSBs with <80 grams of sugar per liter were taxed at €0.08 per liter. SSBs with >80 grams per liter were taxed at €0.16 per liter. | Nova School of Business and Economics | Difference-in-differences | SSB taxes will change consumer behavior, generate revenue, and incentivize manufacturers to reformulate products. Taxes will also reduce diseases such as obesity and diabetes. | Product, store, region, quarter | The taxes increased the quantity of liters sold for low sugar products, but had no impact on quantity of high, medium or zero sugar products sold. | Authors report stockpiling of SSBs in the quarter before the tax was implemented, the possibility of substitution towards diet soda, and potential for cross-border shopping as barriers to the implementation of the tax and its potential impact on health outcomes. Their main facilitator of implementation was manufacturers reformulated SSBs to pay a lower tax. | None reported | None reported | **High risk of bias** | SSB taxes targeting manufacturers had no impact on purchases of high-, medium-, or low-sugar beverages, but increased purchases of low-sugar beverages. Though diet and health outcomes were not assessed, authors report that manufacturers reformulated SSBs to pay a lower tax, which may influence diet quality. | Yes |
| Hernández-F | 2021 | Mexico | Tax | Beverages with added sugar were taxed at one peso per liter. Solid foods with a caloric density >274 kilocalories per 100 grams were also taxed at 8% of their pre-tax average price. | Bloomberg Philanthropies | Interrupted time series | The tax will reduce sugar consumption, which will reduce dental caries and oral health outpatient visits. | Population growth, month indicator, per capita public health spending for population without social security | The taxes had no impact on outpatient visits related to dental caries. | None reported | None reported | Authors report the analysis does not include information on eating habits that could be related to caries, and that data may not be nationally representative and only observes the population that accesses health services. | **Some concerns identified** | Taxes on SSBs and high-sugar foods did not improve health outcomes (outpatient dental visits). There were no findings on consumer purchases or diet quality. | No |
| Howard | 2011 | USA | Subsidy | Subsidy for school lunches for low-income students. | University of Connecticut; Cal State University, Fullerton | Instrumental variable | Subsidies for healthy foods made available at school will increase consumption. | BMI, age, gender, disability, parental years of schooling, household income, weekly exercise, TV time, availability of food within zip code | The subsidy had no impact on fruit consumption. | None reported | None reported | Authors caution against drawing strong conclusions on the overall effect of the program on children’s nourishment because the dietary survey data analyzed are limited. | **Some concerns identified** | The school-lunch subsidy did not increase children's consumption of healthy foods. It is unlikely that the subsidy improves diet quality or health. | No |
| Kruz | 2021 | France; Hungary | Tax | In France, SSBs with <11g of sugar were taxed at €0.0755 eurocents per liter; >11g of sugar taxes were progressive and increased by €0.20 cents per liter as grams of sugar increased. In Hungary, SSBs containing syrup were taxed at 200 Forint per liter. Energy beverages containing methyl xanthine and taurine taxed at 250 Forint per liter. Energy beverages containing methyl xanthine were only taxed at 40 Forint per liter. Other soft beverages taxed at 7 Forint per liter. This tax also applied to the salt and caffeine content of pre-packaged foods. | Institute of Health Economics and Health Care Management, Helmholtz Zentrum; Munich School of Management, Munich Center of Health Sciences, Ludwig-Maximilians-Universität München | Synthetic control | The tax will increase SSBs prices and lowers SSB consumption. | None reported | In France, the tax had no impact on sales of taxed SSBs or any soft beverages. In Hungary, the tax increased sales of taxed SSBs but had no impact on sales of all soft beverages. Sales may have increased relative to other European countries that did not implement a sugar tax. | None reported | None reported | Authors use small sample sizes, which generated large confidence intervals, and Euromonitor data that was only available annually. They conclude that changes in sales after the sugar tax intervention were not definitely a result of the levy, and that soft drink categories, as defined by Euromonitor, may have failed to capture some beverages with added sugar. | **N/a** | Taxes on SSBs decreased purchases in Hungary, but not in France. The data used in the analysis may have failed to capture all beverages with added sugar. | No |
| Law | 2021 | India | Tax | Aerated beverages were taxed at 40 percent of their pre-tax price. | Welcome Trust’s Our Planet, Our Health program | Interrupted time series | The GST will increase prices of SSBs, reduce SSB consumption, and reduce noncommunicable disease. | Month fixed effects, State-level CPI, state FE | The taxes had no impact on volume purchased of taxed and/or untaxed aerated beverages | Barriers include low public awareness of detrimental health outcomes associated with consuming SSBs. The tax was not specifically introduced as a health-related tax, and while some media highlighted the high tax of GST on aerated drinks, no clear rationale on the health impacts was provided. Authors did not observe “signaling effects” that may have encouraged people to be more conscious about their beverage choices. | Sub-group analysis was conducted for higher and lower income states. Findings were consistent with main findings. | No data on beverage prices, so cannot test how taxes influenced prices of aerated beverages. | **Some concerns identified** | Taxes on aerated beverages had no impact on consumption of taxed and untaxed beverages, but the taxes were a part of larger tax reforms (India's Goods and Services Tax (GST)) and not introduced to improve health. | No |
| Nakamura | 2018 | Chile | Tax | SSBs with >6.25 grams added sugar concentration per 100 mL were taxed at 18% (previously 13%). SSBs with <6.25 grams added sugar concentration per 100mL were taxed at 10% (previously 13%). | Centre for Health Economics, University of York, York, United Kingdom | Fixed-effects models | Consumers respond more strongly to price increases (taxes) than price decreases (subsidies) relative to their internal reference price. | General time trend, month, temperature, unemployment rate | The taxes decreased purchases of untaxed soft beverages for low and high-income groups, but had no impact for middle income participants. The tax decreased sugar consumption for all groups combined, as well as for high income and middle income participants, but did not reduce sugar consumption for low-income participants alone. | The SSB tax was only a minor part of a major tax reform, and public visibility was limited. High-income individuals may have better access to information on detrimental health outcomes associated with SSB consumption relative to low-income individuals, and may be more likely to reduce purchases upon implementation of the tax. Also, a new tax on alcoholic beverages was implemented at the same time, and there could have been substitution between soft drinks and alcohol items. | Sub-group analysis by socioeconomic status and weight | Authors report that the use of non-randomized control groups may not capture the influence of time-varying unobservable factors, and cannot rule out endogeneity in the analysis due to several other changes in tax codes introduced by the same time. The data used to classify the beverages into the relevant tax categories was not originally part of the Kantar World Panel data (the main data source), but was collected by the research team using national databases and does not represent all consumer purchases. The data also only report household purchasing rather than actual consumption. Moreover, given that the data are at household level, authors do not know how beverages were distributed within the household. | **Some concerns identified** | Taxes on SSBs decreased sugar consumption for all income levels, but impacts were not observed for low-income participants in isolation. There is some evidence that the taxes increase consumption of healthy foods but none on improvement in health. A new tax on alcohol was implemented at the same time, which may have influenced consumer behavior. | Partially, but did not impact sugar consumption for low-income constituents |
| Øvrebø | 2020 | Norway | Tax | High-sugar foods were taxed at 80 percent and beverages at 40 percent of their pre-tax prices. | National Institute of Public Health | Interrupted time series | The tax will increase prices of taxed products and decrease consumption. | Week fixed effects, shop fixed effects, a season dummy, dummies for Halloween and Easter, and the value of sales of nonedible products | The taxes had no impact on sales of taxed candy or soda. | None reported | None reported | While authors modeled the pre-existing trends in sales and controlled for a proxy of total sales, they did not control for unknown factors that selectively affect sales of the taxed product groups in the year of the intervention. Their data did not include retail data from small, independent stores, kiosks or gas stations. | **Some concerns identified** | Taxes on high-sugar foods did not change consumer behavior, so it is unlikely that taxes influenced diet quality or health. | No |
| Øvrum | 2013 | Norway | Subsidy | Subsidies for fruits and vegetables for school children at 3.5 NOK per day. | Research Council of Norway | Randomized control trial | Subsidies for fruits and vegetables offered at school will increase children's consumption of healthy foods. | Child, parent and household characteristics | The subsidies increased consumption of fruits and vegetables. | None reported | None reported | The results are limited to primary school children and their parents surveyed while the children attended primary school. Assignment to treatment and control conditions were not randomized. Authors report bias may also arise from the relatively crude healthy food consumption measures that were used in the survey and from the fact that child F&V intakes were reported only by the parents and not by the children themselves. Parents in sample were less likely to have attended college than on average in Norway. | **High risk of bias** | Subsidies for fruits and vegetables through a school-lunch program did increase consumption of healthy foods in children, but there was no impact on health. | Yes |
| Pell | 2020 | UK | Tax | SSBs with 8g of sugar per 100 ml were taxed at £0.24 per liter. Beverages with 5 to <8 g of sugar per 100 ml were taxed at £0.18 per liter. | Centre for Diet and Activity Research, MRC Epidemiology Unit, University of Cambridge School of Clinical Medicine, Institute of Metabolic Science, Cambridge, United Kingdom | Interrupted time series | The taxes will increase prices and decrease purchases and consumption. The tax may also generate 'signaling effects' of the health risks associated with SSBs that could influence social norms around SSBs, portion sizes, and reformulation of drinks. | Dummy variables for weeks before Christmas, dummy for weeks immediately after Christmas, dummy for easter, monthly temperature | The tax decreased purchases of low-levy tier beverages and untaxed beverages, but had no impact on purchases of high-tax beverages. | Authors note that anticipatory effects may have led to anticipatory changes, both by industry in reformulating product and in consumer awareness, attitudes, or beliefs. | None reported | Authors report that the before-after study design was not able to distinguish the impact of the SDIL from other trends in soft drinks purchases. | **Some concerns identified** | Tier taxes on SSBs decreased purchases of relatively lower SSBs, but did not increase consumption of untaxed beverages. Manufacturers reformulated products to pay low-levy taxes, which may have influenced diet quality, but diet and health outcomes were not evaluated. | Yes |
| Pfinder | 2020 | Hungary | Tax | Taxes on foods high in sugar, salt and caffeine, including SSBs. | Scottish Institute for Research in Economics (SIRE) | Systematic review | The taxation of unprocessed sugar or sugar-added foods is expected to cause an increase in price, which will decrease sales and consumption. | A number of sub-group analyses were planned, but not conducted due to including only one study. | The tax decreased the consumption of taxed sugar-added foods. This effect is based on one included impact evaluation only. | None reported | None reported | The review itself is likely to be free from bias. The evidence presented in the review is rated as very low quality due to the bias in the one included study. Authors state additional studies are likely to substantially change the effect presented. | **High confidence in the review** | The tax decreased the consumption of taxed sugar-added foods. This effect is based on one included impact evaluation only. | Yes |
| Powell | 2021 | Seattle, WA USA | Tax | SSBs and ASBs taxed at 1 cent per fluid ounce. There was a pre-tax media campaign on revenue generation and later on health benefits. | Bloomberg Philanthropies’ Obesity Prevention Initiative and Arnold Ventures Philanthropy | Difference-in-differences | Tax will reduce grams of sugar sold. | None reported | The tax had no impact on grams of sugar sold from taxed or untaxed beverages, sweets, and stand-alone sugar. | None reported | None reported | The authors report the study was unable to address potential differences in changes in consumption patterns across populations, differential changes based on the store characteristics, or grams of sugar sold in non-store venues. | **High risk of bias** | Taxes on SSBs did not change consumer behavior, so it unlikely that the tax improves diet quality or health. | No |
| Powell | 2020 | Cook County, IL USA | Tax | SSBs with at least 40 kcal per 12 ounces were taxed at 1.75 cents per ounce. | Bloomberg philanthropies | Interrupted time series | The tax will increase prices and decrease consumption. The tax may also generate 'signaling effects' of the health risks associated with SSBs that will decrease consumption. | None reported | The tax had no impact on purchases of taxed or untaxed beverages, even after the tax was repealed. | Public health emerged more prominently in campaigns just prior to implementation and during the 4 months that the tax was in place as part of a strategy to ward off pressure from antitax stakeholders. Pro-tax stakeholders had limited to no time (2 to 3 months) to implement a well-planned campaign. Additionally, the tax ordinance required that the shelf price indicate the total price inclusive of the tax, but retailers may not have complied with this before the tax was repealed. | None reported | Tax was only in place for 4 months, which may not have been enough time to cement behavioral changes related to newly signaled information on health harms associated with SSB consumption. No robust public health campaign, so results may not be generalizable to jurisdictions that provide strong explicit signals on SSB consumption-related health harms. Data do not cover restaurant purchases and do not allow us to assess differences in volume sold across different household types. | **High risk of bias** | The tax was only in place for four months before repeal, which may not have been enough time for signaling effects to occur. There were information campaigns in support and against the tax. The tax had no impact on purchases of taxed or untaxed beverages, even after the tax was repealed. | No |
| Puig-Codina | 2020 | Catalonia, Spain | Tax | SSBs with >8g of sugar per liter were taxed at €0.12 per liter. SSBs with 5 to <8g of sugar per liter taxed at €0.08 per liter. | Department of the Agriculture Ministry | Synthetic control | The taxes will decrease SSB consumption, reduce negative health outcomes, generate revenues, and incentivize product reformulation. Reducing SSB consumption will also generate positive externalities such as reducing public health care costs, non-health care costs, and productivity costs associated with obesity, diabetes, cardiovascular diseases, cancers, and dental caries. | AC and Month fixed effects | The tax increased purchases of diet cola and decreased purchases of regular cola. | None reported | None reported | Authors report lack of robustness for the impact on diet colas but recommend caution about always assuming a significant substitution effect between regular and diet cola beverages 2 years after the intervention. | **N/a** | Taxes on SSBs decreased purchases of regular cola (taxed) and increased purchases of diet cola (untaxed). Authors do not report changes in diet quality or health. | Yes |
| Rojas | 2021 | Berkley, CA USA & Seattle, WA USA | Tax | In Berkley, SSBs taxed at 1 cent per ounce.  In Seattle, SSBs with at least 40 kcal per 12 ounces taxed at 1.75 cents per ounce. | University of Massachusetts Amherst | Difference-in-differences | Taxing SSBs may reduce obesity. | Store zip code, SSB brand, SSB size | The tax decreased purchases of taxed beverages in both Berkley and Seattle. | In Berkley, authors argue that the small geographic coverage led to low pass-thru of tax to consumers, which did not change prices of SSBs very much, a barrier to implementation of the tax. Authors suspect that the application of a tax to a relatively small geographic area such as Berkeley makes cross-border shopping easier, thereby undermining the policy objective of observing large price increases at the retail level. Also, affected beverage companies (and stores) might be less willing to pass on the tax in order to avoid losing consumers to nearby (untaxed) stores. | None reported | Authors report serially correlated errors, but suggest a method for addressing them. | **High risk of bias** | Taxes on SSBs in two U.S. cities decreased purchases of taxed beverages, but there were no reported changes in diet quality or health. | Yes |
| Royo-Bordonada et al. | 2019 | Catalonia, Spain | Tax | SSBs with >8g of sugar per liter were taxed at €0.12 per liter. SSBs with 5 to <8g of sugar per liter taxed at €0.08 per liter. | Spanish Health Research Fund (Fondo de Investigación Sanitaria - FIS) of the Carlos III Institute of Health and the Spanish Consumers’ Organization (Organización de Consumidores y Usuarios - OCU) | Difference-in-differences | SSB tax will increase prices of SSBs, reduce SSB consumption, and increase health awareness of health risks associated with SSB consumption. | Age, sex, educational level, nationality, occupational status | The tax had no impact on consumption of taxed or untaxed beverages. | None reported | None reported | Authors report significant socioeconomic differences in treated and control populations; the method of recruitment into the study and varying conditions may explain this. To control for confounding factors, authors adjusted the regression models for socio-demographic variables using propensity scores. Authors also report the common trends assumption may not be representative of SSBs sold in local or regional supermarkets, food and grocery stores, and/or bars and restaurants. Finally, authors measured the frequency of consumption instead of the amount consumed, limiting comparison with other studies. | **High risk of bias** | Taxes on SSBs did not change consumer behavior, so it is unlikely taxes influence diet quality or health. | No |
| Silver | 2017 | Berkeley, USA | Tax | SSBs taxed at 1 cent per ounce. | Chain stores sharing sales-point data are not identified in the paper | Fixed-effects models | Taxes will decrease SSB consumption, prevent obesity and diabetes, and generate positive health and social impacts. | Store ID, day of week, holiday and holiday eve, month, year, number of transactions (linear and quadratic) | The tax decreased sales of taxed and untaxed beverages. | Consumer spending declined after the tax, belying concerns about increased consumer spending. | None reported | Authors report the observational study cannot establish causal links between the SSB tax implementation and changes in health outcomes. The alternative of a more distant control would have better captured the combined effects of the campaigns and the tax itself. | **High risk of bias** | Taxes on SSBs decreased sales of both taxed and untaxed beverages, indicative of an overall decrease in consumer spending. Diet quality or health outcomes are not reported. | Yes |
| Teng | 2019 | USA; Chile; France; Mexico; Spain | Tax | Equivalent of a 10 percent SSB tax on pre-tax prices. | Health Research Council of New Zealand | Systematic review | Taxation can decrease consumption through price changes, public health messaging, product reformulation. | Subgroup analysis was conducted based on jurisdiction, study quality, consumption measure, age group, peer review, and funding source. | Taxes decreased a combined measure of purchases and dietary intake of taxed beverages, largely weighted to results from France. No impact on the same measure for total untaxed beverage consumption though increases were found in three of the four jurisdictions (Berkeley, Mexico, and the U.S.) | None reported | Subgroup analysis by age group. | Authors note that the use of bespoke quality appraisal tool limits the ability to compare their results with other similar reviews. | **High-confidence in results** | Taxes on SSBs decreased consumption of taxed beverages, but had no impact on consumption of untaxed beverages. Diet quality or health outcomes are not reported. | Yes |
