# Supplementary material for: Systematic review on fiscal policy interventions in nutrition
Source: Front Nutr. 2022 Nov 29;9:967494. doi: 10.3389/fnut.2022.967494 (PMC9756132; doi:10.3389/fnut.2022.967494)
Supplement: Supplementary file 2 [file Table_2.DOCX]

## Supplement 1: Rapid Evidence Assessment on Fiscal Policies – Protocol*

*Note: We subsequently changed the name of the rapid evidence assessment to systematic review. The systematic review followed the rigorous Campbell Collaboration and Cochrane approaches to systematic reviewing (Higgins et al. 2019.; Hammerstrøm et al. 2010). We did not shorten steps as proposed in Barends et al. 2017.

### 1 Background

- 1. **The problem, condition or issue**

Both the WHO and the Lancet Series on Adolescent nutrition recommend that governments adopt fiscal policies, such as taxes and subsidies, to combat the double burden of malnutrition (World Health Organization, 2017; Hargreaves et al., 2022). However, rigorous, systematic evidence regarding the effects of these interventions is lacking in both high- income countries (HICs) and low- and middle-income countries (L&MICs). The 3ie Food Systems Evidence Gap Map (EGM) includes 22 impact evaluations on governmental price manipulations L&MICs (Moore et al., 2021). Among these, nine fiscal policies support the consumption of a healthy diet. This rapid evidence assessment will identify, appraise and synthesise evidence on the effects of fiscal policies on diet related outcomes.

- 1. **The interventions**

There are two types of fiscal policies interventions, implemented by governments, that form the scope of this rapid evidence assessment. They are:

- Taxes that increase prices of unhealthy foods and beverages to discourage consumption
- Subsidies to decrease prices of healthy foods and beverages to encourage consumption

**1.3**  **Expected theory of change**

FIGURE 1: Fiscal Policy Theory of Change


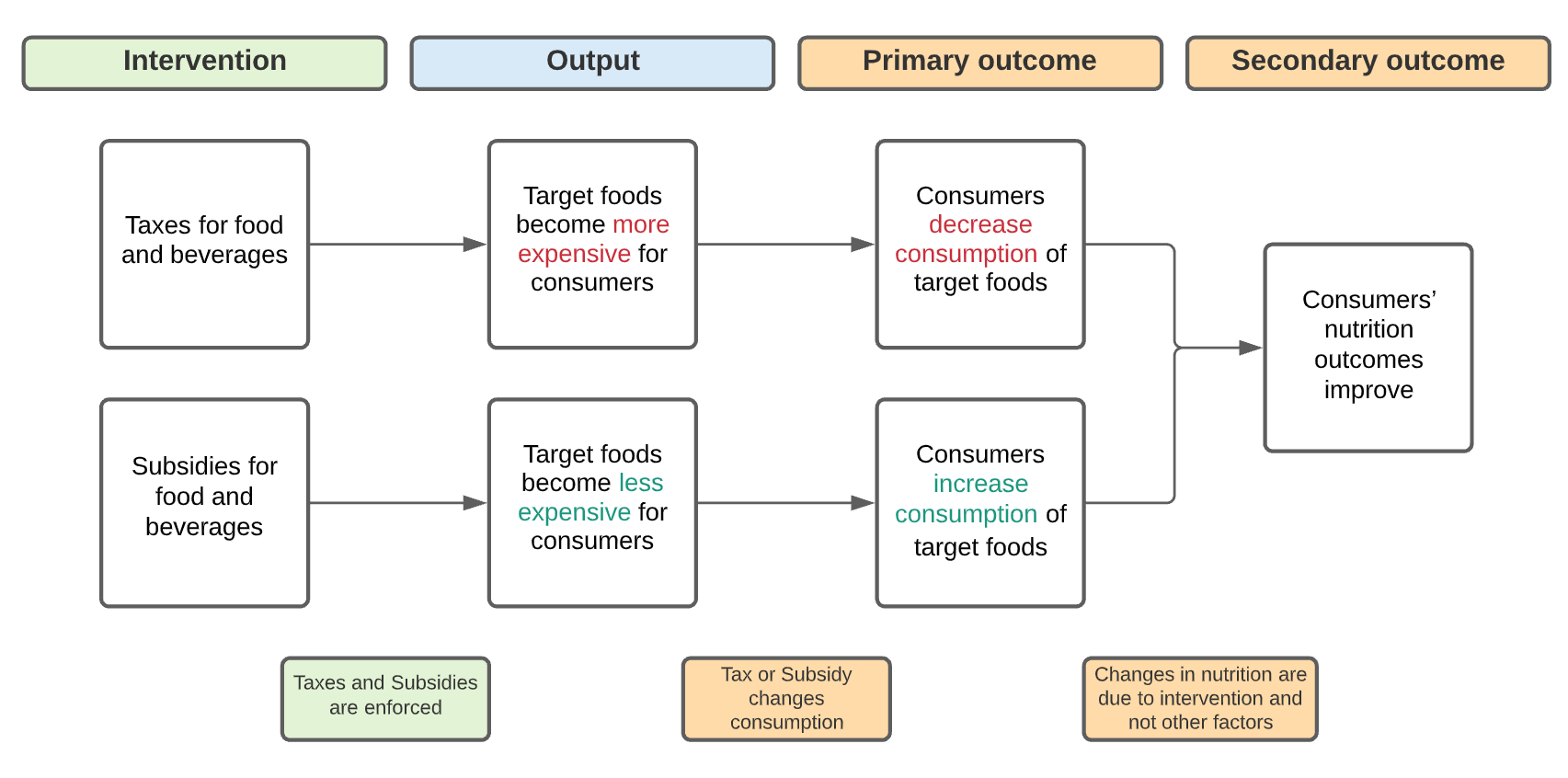


*Source: 3ie*

**1.4 Rationale for the review**

Given ongoing international debates regarding the use of these policies, GIZ and other key decisionmakers have indicated interest in this policy area. This rapid evidence assessment (REA) is expected to inform decisions on the use of fiscal policies to combat malnutrition in L&MICs.

### 2 Research questions

1. What are the effects of fiscal policies on food and beverages on the availability, access and affordability of healthy diets?
2. Are there unintended consequences of these actions, such as food substitutions or any regressive effects?
3. What policy design features moderate impact? For example, do effects vary by the specific approach taken, food targeted, socio-economic status, or context, including the joint implementation of fiscal policies with other initiatives?
4. What evaluation design strategies are used? What relationships and data sources are key to allowing for evaluation?
5. What are common implementation challenges, sustainability issues, and implications for practitioners in both HICs and L&MICs?

### 3 Method

To respond to these research questions, we will conduct a rapid evidence assessment based on topically relevant studies identified by the FS EGM and a supplementary systematic literature search of key academic databases. Literature will be screened for quality and summarized visually and in a narrative format. A rapid evidence assessment is based upon the rigorous methodology adopted in a systematic review; however, many steps are shortened (Barends et al. 2017).

**3.1 Criteria for including and excluding studies in the review (PICOS)**

| Criteria | Included | Excluded |
| --- | --- | --- |
| Participants | Individuals in L&MICs and HICs | Niche populations, such as astronauts, people in the military, professional athletes, etc. |
| Intervention | Taxes for unhealthy food and beverages (e.g. sugar-sweetened)  Consumer subsidies for healthy foods (e.g. fruits, vegetables, legumes, pulses, fortified wheat) and beverages (e.g. fortified milk for pregnant mothers) | In-kind food provision (e.g. free school meal handouts) or fully-subsidised products  Food vouchers or cash transfers /cashbacks/food stamps even if they are referred to as subsidies (e.g. SNAP in US). These interventions do not explicitly decrease the market price of healthy foods and beverages  Fiscal polices that are not subsidies or taxes, such as price ceilings  Reduction on import taxes for vegetables and fruits.  General consumption behavior change interventions  Agricultural input subsidies  Consumer subsidies not directly aimed at supporting a healthy diet (e.g. for corn, rice, wheat, salt, wine grapes unless fortified with micronutrients)  Price changes, which would not be considered an intervention by itself  Lab in the field experiments such as virtual supermarkets |
| Comparison | Business as usual, including pipeline and waitlist controls  An alternate intervention | If there is no comparison |
| Outcome | Availability and accessibility of healthy foods and beverages  Purchasing behavior/patterns  Diet quality and adequacy  Anthropometrics  Health, such as:   - Diseases associated with nutrition, such as diabetes and heart disease   Well-being, such as:   - Overall well-being metrics, such as psychological measures and the acceptability of diet | Affordability of healthy foods and beverages, such as:   - Post-tax or post-subsidy changes in price   Outcomes unrelated to nutrition, such as tax revenue, public finance |
| Study designs | Experimental and quasi-experimental impact evaluations  Systematic reviews of experimental and quasi-experimental impact evaluations  Ex-post cost evidence | Qualitative impact evaluations  Descriptive or observational studies that do not assess effectiveness  Modelling studies  Ex-ante cost evidence |

- - 1. **Types of study participants**

All participants except those from niche populations will be considered, regardless of country of origin, gender, ethnicity, age, or other demographic trait.

- - 1. **Types of interventions**

While taxes are generally easy to identify, defining subsidies can be challenging. We are defining subsidies as interventions in which the government pays a *portion* of the price of a food. Studies must explicitly mention that they evaluate subsidies; however, there are many interventions which are called subsidies but instead directly provide food, cash, or vouchers. While these programs can all be conceptualized as reducing costs and increasing accessibility of food, the behavioural responses of consumers to the various delivery mechanisms are likely to be different. Subsidies can be implemented alongside other governmental programs, such as subsidising a portion of the cost of a school meal which students are expected to pay.

| Intervention definition | Examples |
| --- | --- |
| Taxes on food and beverages | Excise tax on sugar-sweetened beverages in Mexico |
| Subsidies for food and beverages | Subsidies to lower the price of pulses for consumers in India |

- - 1. **Types of outcome measures**

The table below outlines outcome indicators that will be extracted. These outcomes can be measured using a variety of indicators. We have indicated the preferred outcomes and alternate outcomes which could be used if preferred outcomes are not reported. Composite measures will always be preferred over disaggregated ones.

| Outcome definition | Examples |
| --- | --- |
| Availability and accessibility of foods and beverages | Preferred outcomes: food assets, production (community gardens), and stores  Other measures: distance and accessibility to markets, will be considered if these are not available |
| Purchasing behaviour | Preferred outcome: sales of food in monetary units  Secondary outcome: frequency/change of purchase of foods |
| Diet quality and adequacy | Preferred outcomes: composite diet scores such as the nutrient rich food index  Secondary outcome: dietary diversity and other food variety measures  Tertiary outcome: intake of specific foods |
| Anthropometrics | Preferred outcomes: body mass index, weight for length, length for age, weight for age  Other measures, such as MUAC and ponderal index, will be considered if these are not available |
| Health | Incidence of diseases *directly* tied to nutrition, especially with regard to nutrition-related NCDs, will be considered. These will include diabetes, anaemia, metabolic syndrome, and cardiovascular disease. Indirect diseases, such as cancer, will not be considered. |
| Well-being | Preferred outcome: perceived well-being  Secondary outcome: anxiety |

**Types of Comparators**

- Business as usual, including pipeline and waitlist controls
- An alternate intervention
- Studies with no comparator are excluded

**3.1.4**  **Types of study design**

Experimental, quasi-experimental, systematic review, and cost evidence will be considered. The following study designs will be included.

- Randomized controlled trial
- Regression discontinuity design
- Controlled before-and-after studies, including
  - Propensity-weighted multiple regression
  - Instrumental variable
  - Difference-in-differences (and any mathematical equivalents)
  - Matching techniques
- Interrupted time series
- Systematic reviews that include a quantitative or narrative synthesis
- Ex-post cost-effectiveness analyses

**3.1.5 Date, language, and form of publication**

- Date: 2000
- Language: English
- Publication forms: only completed studies, not protocols
- Published and unpublished studies (if identified)

**3.2**  **Search strategy**

The search string was developed by an information specialist with subject-matter input by the technical team of this project. Search terms are provided in Appendix 1. Studies of fiscal policies (excluding tariffs) from the evidence gap map by Moore and colleagues (2021) were used to calibrate

the search.

**3.2.1 Electronic searches of bibliographic databases and library catalogues**

The following twelve databases were searched:

- CAB Abstract (EBSCO)
- Agricola (EBSCO)
- Medline (EBSCO)
- Academic Search Complete (EBSCO)
- PsycInfo (EBSCO)
- Africa-Wide (EBSCO)
- CINAHL (EBSCO)
- Scopus
- Embase (Ovid)
- CAB Global Health (Ovid)
- Cochrane Library (this contains 2 d/bases - Trials Register and the SR database)

**3.2.2**  **Other searches**

In addition to the search of academic databases, we will search for additional, relevant studies that had been previously identified from the search by Moore and colleagues (2021) and its recent update. These studies may have been excluded because they considered participants from high-income countries or used ineligible study designs. The following studies will be added to the search:

- studies from original map excluded using the code ‘High income country’ on title and abstract or full text, with the term ‘tax*’ or ‘subsid*’ on title or abstract
- studies from original map included on title and abstract that have the term ‘tax’ or ‘subsid*’ in title or abstract
- studies from the EGM update (as of 21/02/2022) with the code ‘Exclude- High income country’ on title and abstract or full text that have the term ‘subsid*’ or ‘tax*’ on title or abstract
- studies from the EGM update (as of 19/02/2022) with the code 'FSN marker TA screening - FSN relevant' with subsid* or tax* on title or abstract
  1. **Selection of studies**

**3.3.1**  **Screening**

Study screening will adopt a rapid synthesis approach.

1. A sample of 1,000 studies (including studies from other searches) will be screened at title and abstract by two reviewers for inclusion in this REA to ensure that studies meet inclusion criteria as expected^^[[1]](#footnote-2)^^. This will provide high quality screening data that will maximise the chances for the machine learning classifier to exclude a large chunk of studies.
2. The machine learning classifier will then be developed in EPPI Reviewer. Only studies with a prioritization score of 0.3 or higher will be screened at title and abstract. If less than 40% of the remaining studies have a score 0.3 and above, title and abstract screening by two reviewers will be implemented. Otherwise, single screening (safety first approach) may be used for a subset of studies. Studies will not be excluded on their outcomes at the title and abstract stage.
3. Studies included at title and abstract will be screened by two reviewers at full text.

Reconciliations between reviewers will be conducted to resolve disagreements at title and abstract and full text. If disagreements cannot be reconciled between two reviewers, a third reviewer will be consulted.

Studies that do not meet full inclusion criteria but are likely to provide relevant descriptive and qualitative information will be tagged for inclusion in the qualitative portion of the REA during the screening process.

**3.3.2**  **Data extraction and coding procedures**

Data extraction templates will be modified from 3ie’s repository coding protocol and the coding protocols typically used for systematic reviews (Supplement 2). This includes bibliographic, geographic information and substantive data, as well as standardized methods information. In addition, we will extract data on interventions, outcomes, population (disaggregated by gender/age, where possible), and effect sizes for parameters relevant to the Theory of Change. Qualitative information on barriers and facilitators to implementation, sustainability and equity implications, and other considerations for practitioners will also be extracted. For this REA, data extraction will be double-coded by two reviewers.

**3.3.3 Critical appraisal**

All included systematic reviews and quantitative impact evaluations will be appraised using a critical appraisal tool based on their study design. Supplement 3 contains three critical appraisal tools: for randomised controlled trials, quasi-experimental methods and systematic reviews. .

**3.4 Analytical approach for quantitative data**

If sufficient data is available, we will conduct meta-analysis to provide summary effect estimates. We will choose the appropriate formulae for effect size calculations in reference to, and dependent upon, the data provided in included studies. We will conduct random effects meta-analyses when we identify two or more studies that we assess to be sufficiently similar. We will assess heterogeneity by calculating the Q statistic, I2, and τ2 to provide an estimate of the amount of variability in the distribution of the true effect sizes (Borenstein et al., 2021). We will explore heterogeneity using moderator analyses if the data allow. Moderators to be considered will include taxes vs. subsidies, food targeted, social economic status (SES), joint implementation with other initiatives, etc. if analyses are sufficiently powered. We will also test for the presence of publication bias if at least 10 studies are included in the analysis.

**3.5 Data presentation**

We will provide a narrative summary of the papers identified. This will include an overall description of the available literature and a general synthesis of findings. Key information from each study, such as intervention type, study design, country, outcomes, measurement type, effect sizes, and confidence rating will be summarized in a table. Results from meta-analyses and their associated forest plot will be presented when the data is sufficient. Qualitative information will be summarized narratively in a practitioner’s brief to support project design and implementation. An updated theory of change will be developed based on the combination of qualitative and quantitative data.

**3.6 Limitations**

Due to the rapid nature of this work, results should be interpreted more cautiously than those of a systematic review. The *abridged search process* and use of some *single screening* may result in some relevant studies being omitted from this evidence assessment. The small number of studies which are expected to be retrieved through this REA may restrict the possibility of using meta-analysis and our ability to draw generalizable conclusions.

### 4 References

Barends, E., Rousseau, D. M. & Briner, R. B (2017) CEBMa Guideline for Rapid Evidence Assessments in Management and Organizations. Amsterdam. Available from: https://www. cebma.org/wp-content/uploads/CEBMa-REA-Guideline.pdf

Borenstein, M., Hedges, L. V., Higgins, J. P., & Rothstein, H. R. (2021) ‘Introduction to meta-analysis’. John Wiley & Sons.

Chakrabarti, S., Kishore, A. and Roy, D. (2018) ‘Effectiveness of food subsidies in raising healthy food consumption: public distribution of pulses in India’, American Journal of Agricultural Economics, 100(5), pp. 1427–1449.

Colchero, M.A. et al. (2016) ‘Beverage purchases from stores in Mexico under the excise tax on sugar sweetened beverages: observational study’, bmj, 352.

Hammerstrøm, K., Wade, A., Jørgensen, A. M. K., & Hammerstrøm, K. (2010). Searching for studies. Education, 54(11.3).

Higgins, J. P., Thomas, J., Chandler, J., Cumpston, M., Li, T., Page, M. J., & Welch, V. A. (Eds.). (2019). Cochrane handbook for systematic reviews of interventions. John Wiley & Sons.

Hargreaves, Dougal, Emily Mates, Purnima Menon, Harold Alderman, Delan Devakumar, Wafai Fawzi, Geva Greenfield, et al. “Strategies and Interventions for Healthy Adolescent Growth, Nutrition, and Development.” *The Lancet* 399, no. 10320 (January 8, 2022): 198–210. <https://doi.org/10.1016/S0140-6736(21)01593-2>.

Li, N. et al. (2013) ‘A large-scale cluster randomized trial to determine the effects of community-based dietary sodium reduction—the China Rural Health Initiative Sodium Reduction Study’, American heart journal, 166(5), pp. 815–822.

Moore, N, Lane, C, Storhaug, I, Franich, A, Rolker, H, Furgeson, J, Sparling, T and Snilstveit, B. (2021) ‘The effects of food systems interventions on food security and nutrition outcomes in low- and middle-income countries’, 3ie Evidence Gap Map Report 16. New Delhi: International Initiative for Impact Evaluation (3ie). Available at: <https://doi.org/10.23846/EGM016>

World Health Organization (2017) Taxes on Sugary Drinks: Why do it? WHO/NMH/PND/16.5 Rev.1. Available at: https://apps.who.int/iris/bitstream/handle/10665/260253/WHO-NMH-PND-16.5Rev.1-eng.pdf;sequence=1 (Accessed: 24 January 2022).

## Appendix 1: Search strategy

Embase (Ovid) <1980 to 2021 Week 49> & CAB Global Health (Ovid)<1910 to 2021 Week 49> - Searched 16th December 2021

1 (((sweeten* or sugary) adj2 (drink* or beverage*)) or "nonessential energy-dense" or "non-essential energy-dense" or "sin food*").ti,ab,sh. (11858)

2 tax*.ti,ab,sh. (275359)

3 1 and 2 (1074)

4 limit 3 to yr="2000 -Current" (1072)

5 limit 4 to exclude medline journals [Limit not valid in Global Health; records were retained] (430) – Sweet Drinks/Taxes

6 (health* adj3 (food* or diet* or eating)).ti,ab,sh. (255536)

7 (fruit* or vegetable*).ti,ab,sh. (394426)

8 (fruit* or vegetable* or legum* or pulses or chickpea* or bean or beans or pea or peas or peanut* or soybean* or lentil*).ti,ab,sh. (621804)

9 6 or 7 or 8 (837289)

10 (subsidies or subsidy or subsidiz* or subsidis* or "tax* incentiv*").ti,ab,sh. (15375)

11 9 and 10 (1190)

12 limit 11 to exclude medline journals [Limit not valid in Global Health; records were retained] (603)

limit 12 to yr="2000 -Current" (493) – HealthyFoods/Subsidies

Scopus – Searches 16th December 2021

10 ( ( TITLE-ABS-KEY ( ( health* W/3 ( food* OR diet* OR eating ) ) ) ) OR ( TITLE-ABS-KEY ( ( fruit* OR vegetable* ) ) ) OR ( TITLE-ABS-KEY ( ( fruit* OR vegetable* OR legum* OR pulses OR chickpea* OR bean OR beans OR pea OR peas OR peanut* OR soybean* OR lentil* ) ) ) ) AND ( TITLE-ABS-KEY ( subsidies OR subsidy OR subsidiz* OR subsidis* OR "tax* incentiv*" ) ) AND ( LIMIT-TO ( PUBYEAR , 2022 ) OR LIMIT-TO ( PUBYEAR , 2021 ) OR LIMIT-TO ( PUBYEAR , 2020 ) OR LIMIT-TO ( PUBYEAR , 2019 ) OR LIMIT-TO ( PUBYEAR , 2018 ) OR LIMIT-TO ( PUBYEAR , 2017 ) OR LIMIT-TO ( PUBYEAR , 2016 ) OR LIMIT-TO ( PUBYEAR , 2015 ) OR LIMIT-TO ( PUBYEAR , 2014 ) OR LIMIT-TO ( PUBYEAR , 2013 ) OR LIMIT-TO ( PUBYEAR , 2012 ) OR LIMIT-TO ( PUBYEAR , 2011 ) OR LIMIT-TO ( PUBYEAR , 2010 ) OR LIMIT-TO ( PUBYEAR , 2009 ) OR LIMIT-TO ( PUBYEAR , 2008 ) OR LIMIT-TO ( PUBYEAR , 2007 ) OR LIMIT-TO ( PUBYEAR , 2006 ) OR LIMIT-TO ( PUBYEAR , 2005 ) OR LIMIT-TO ( PUBYEAR , 2004 ) OR LIMIT-TO ( PUBYEAR , 2003 ) OR LIMIT-TO ( PUBYEAR , 2002 ) OR LIMIT-TO ( PUBYEAR , 2001 ) OR LIMIT-TO ( PUBYEAR , 2000 ) ) ...

1,372 document results (Healthy Foods/Subsidies)

9 ( ( TITLE-ABS-KEY ( ( health* W/3 ( food* OR diet* OR eating ) ) ) ) OR ( TITLE-ABS-KEY ( ( fruit* OR vegetable* ) ) ) OR ( TITLE-ABS-KEY ( ( fruit* OR vegetable* OR legum* OR pulses OR chickpea* OR bean OR beans OR pea OR peas OR peanut* OR soybean* OR lentil* ) ) ) ) AND ( TITLE-ABS-KEY ( subsidies OR subsidy OR subsidiz* OR subsidis* OR "tax* incentiv*" ) )

1,518 document results

8 TITLE-ABS-KEY ( subsidies OR subsidy OR subsidiz* OR subsidis* OR "tax* incentiv*" )

58,023 document results

7 ( TITLE-ABS-KEY ( ( health* W/3 ( food* OR diet* OR eating ) ) ) ) OR ( TITLE-ABS-KEY ( ( fruit* OR vegetable* ) ) ) OR ( TITLE-ABS-KEY ( ( fruit* OR vegetable* OR legum* OR pulses OR chickpea* OR bean OR beans OR pea OR peas OR peanut* OR soybean* OR lentil* ) ) )

1,898,621 document results

6 TITLE-ABS-KEY ( ( fruit* OR vegetable* OR legum* OR pulses OR chickpea* OR bean OR beans OR pea OR peas OR peanut* OR soybean* OR lentil* ) )

1,825,896 document results

5 TITLE-ABS-KEY ( ( fruit* OR vegetable* ) )

609,498 document results

4 TITLE-ABS-KEY ( ( health* W/3 ( food* OR diet* OR eating ) ) )

90,332 document results

3 ( TITLE-ABS-KEY ( ( ( sweeten* OR sugary ) W/2 ( drink* OR beverage* ) ) OR "nonessential energy-dense" OR "non-essential energy-dense" OR "sin food*" ) ) AND ( TITLE-ABS-KEY ( tax* ) )

780 document results (Sweet Drinks/Taxes)

2 TITLE-ABS-KEY ( tax* )

656,004 document results

1 TITLE-ABS-KEY ( ( ( sweeten* OR sugary ) W/2 ( drink* OR beverage* ) ) OR "nonessential energy-dense" OR "non-essential energy-dense" OR "sin food*" )

7,096 document results

Africa-Wide, CINAHL (Ebsco) – Searched 16th December 2021

S9 (S4 or S5 or S6) AND S7 Limiters - Published Date: 20000101-20211231

275 – Healthy Foods/Subsidies

S8 (S4 or S5 or S6) AND S7

340

S7 TI ( subsidies or subsidy or subsidiz* or subsidis* or "tax* incentiv*" ) OR AB ( subsidies or subsidy or subsidiz* or subsidis* or "tax* incentiv*" ) OR SU ( subsidies or subsidy or subsidiz* or subsidis*or "tax* incentiv*" )

10,345

S6 TI ( ( fruit* or vegetable* or legum* or pulses or chickpea* or bean or beans or pea or peas or peanut* or soybean* or lentil*) ) OR AB ( ( fruit* or vegetable* or legum* or pulses or chickpea* or bean or beans or pea or peas or peanut* or soybean* or lentil*) ) OR SU ( ( fruit* or vegetable* or legum* or pulses or chickpea* or bean or beans or pea or peas or peanut* or soybean* or lentil*) )

142,131

S5 TI ( fruit* or vegetable* ) OR AB ( fruit* or vegetable* ) OR SU ( fruit* or vegetable* )

83,765

S4 TI ( (health* N3 (food* or diet* or eating)) ) OR AB ( (health* N3 (food* or diet* or eating)) ) OR SU ( (health* N3 (food* or diet* or eating)) ) Limiters - Published Date: 20000101-20211231

38,333

S3 S1 AND S2 Limiters - Published Date: 20000101-20211231

540 – Sweet Drinks/Taxes

S2 TI tax* OR AB tax* OR SU tax*

79,220

S1 TI (((sweeten* or sugary) N2 (drink* or beverage*)) or "nonessential energy-dense" or "non-essential energy-dense" or "sin food*") OR AB (((sweeten* or sugary) N2 (drink* or beverage*)) or "nonessential energy-dense" or "non-essential energy-dense" or "sin food*") OR SU (((sweeten* or sugary) N2 (drink* or beverage*)) or "nonessential energy-dense" or "non-essential energy-dense" or "sin food*" )

4,376

Agricola, Academic Search Complete, PsycInfo, Medline (Ebsco) – Searched 16th December 2021

S9 (S4 or S5 or S6) AND S7 Limiters - Published Date: 20000101-20211231

1,547 – Healthy Foods/Subsidies

S8 (S4 or S5 or S6) AND S7

1,707

S7 TI ( subsidies or subsidy or subsidiz* or subsidis* or "tax* incentiv*" ) OR AB ( subsidies or subsidy or subsidiz* or subsidis* or "tax* incentiv*" ) OR SU ( subsidies or subsidy or subsidiz* or subsidis*or "tax* incentiv*" )

58,113

S6 TI ( ( fruit* or vegetable* or legum* or pulses or chickpea* or bean or beans or pea or peas or peanut* or soybean* or lentil*) ) OR AB ( ( fruit* or vegetable* or legum* or pulses or chickpea* or bean or beans or pea or peas or peanut* or soybean* or lentil*) ) OR SU ( ( fruit* or vegetable* or legum* or pulses or chickpea* or bean or beans or pea or peas or peanut* or soybean* or lentil*) )

1,627,763

S5 TI ( fruit* or vegetable* ) OR AB ( fruit* or vegetable* ) OR SU ( fruit* or vegetable* )

705,364

S4 TI ( (health* N3 (food* or diet* or eating)) ) OR AB ( (health* N3 (food* or diet* or eating)) ) OR SU ( (health* N3 (food* or diet* or eating)) ) Limiters - Published Date: 20000101-20211231

137,598

S3 S1 AND S2 Limiters - Published Date: 20000101-20211231

1,360 – Sweet Drinks/Taxes

S2 TI tax* OR AB tax* OR SU tax*

748,180

S1 TI (((sweeten* or sugary) N2 (drink* or beverage*)) or "nonessential energy-dense" or "non-essential energy-dense" or "sin food*") OR AB (((sweeten* or sugary) N2 (drink* or beverage*)) or "nonessential energy-dense" or "non-essential energy-dense" or "sin food*") OR SU (((sweeten* or sugary) N2 (drink* or beverage*)) or "nonessential energy-dense" or "non-essential energy-dense" or "sin food*" )

13,362

Cochrane Library – Searched 16th December 2021

#1 (fruit* or vegetable* or legum* or pulses or chickpea* or bean or beans or pea or peas or peanut* or soybean* or lentil*):ti,ab,kw 17998

#2 (subsidies or subsidy or subsidiz* or subsidis* or "tax* incentiv*"):ti,ab,kw 511

#3 #1 and #2 37 - Health Foods/Subsidies (Trials only)

#4 (((sweeten* or sugary) adj2 (drink* or beverage*)) or "nonessential energy-dense" or "non-essential energy-dense" or "sin food*"):ti,ab,kw 0 – Sweet Drinks

CAB Abstracts (Ebsco) – Searched 9th December 2021

S14 S6 AND S13 Limiters - Publication Year: 20000101-20211231

1,422

S13 S5 OR S9 OR S12

898,006

S12 S10 OR S11

859,206

S11 DE "grain legumes" OR DE "black gram" OR DE "chickpeas" OR DE "cluster beans" OR DE "cowpeas" OR DE "faba beans" OR DE "green gram" OR DE "lentils" OR DE "Lima beans" OR DE "lupins" OR DE "mung beans" OR DE "pigeon peas" OR DE "soyabeans"

182,688

S10 TI ( ( fruit* or vegetable* or legum* or pulses or chickpea* or bean or beans or pea or peas or peanut* or soybean* or lentil*) ) OR AB ( ( fruit* or vegetable* or legum* or pulses or chickpea* or bean or beans or pea or peas or peanut* or soybean* or lentil*) ) OR SU ( ( fruit* or vegetable* or legum* or pulses or chickpea* or bean or beans or pea or peas or peanut* or soybean* or lentil*) )

830,207

S9 S7 OR S8

530,230

S8 TI ( fruit* or vegetable* ) OR AB ( fruit* or vegetable* ) OR SU ( fruit* or vegetable* )

530,230

S7 (DE "fruit" OR DE "dried fruit" OR DE "frozen fruit" OR DE "fruit crops" OR DE "fruits") AND (DE "vegetables" OR DE "bulbous vegetables" OR DE "fruit vegetables" OR DE "leafy vegetables" OR DE "root vegetables" OR DE "stem vegetables" OR DE "leafy vegetables" OR DE "broccoli" OR DE "Brussels sprouts" OR DE "cabbages" OR DE "cauliflowers" OR DE "celery" OR DE "Chinese cabbages" OR DE "cress" OR DE "endives" OR DE "globe artichokes" OR DE "kale" OR DE "lettuces" OR DE "roselle" OR DE "spinach" OR DE "spinach beets" OR DE "watercress" OR DE "stem vegetables" OR DE "bamboo shoots" OR DE "cardoons" OR DE "kohlrabi" OR DE "rhubarb" OR DE "root vegetables" OR DE "beetroots" OR DE "carrots" OR DE "celeriac" OR DE "fodder beet" OR DE "Jerusalem artichokes" OR DE "mangolds" OR DE "parsnips" OR DE "potatoes" OR DE "radishes" OR DE "salsify" OR DE "swedes" OR DE "sweet potatoes" OR DE "turnips" OR DE "yams" OR DE "fruit vegetables" OR DE "aubergines" OR DE "cucurbit vegetables" OR DE "okras" OR DE "sweetcorn" OR DE "tomatoes" OR DE "vegetable legumes" OR DE "cucurbit vegetables" OR DE "cucumbers" OR DE "marrows" OR DE "pumpkins" OR DE "squashes" OR DE "bulbous vegetables" OR DE "chives" OR DE "garlic" OR DE "leeks" OR DE "onions" OR DE "shallots" OR DE "Welsh onions")

52,914

S6 TI ( subsidies or subsidy or subsidiz* or subsidis* or "tax* incentiv*" ) OR AB ( subsidies or subsidy or subsidiz* or subsidis* or "tax* incentiv*" ) OR SU ( subsidies or subsidy or subsidiz* or subsidis*or "tax* incentiv*" )

22,650

S5 TI ( (health* N3 (food* or diet* or eating)) ) OR AB ( (health* N3 (food* or diet* or eating)) ) OR SU ( (health* N3 (food* or diet* or eating)) ) Limiters - Publication Year: 20000101-20211231

49,851

S4 S1 AND S2 Limiters - Publication Year: 20000101-20211231

396

S3 S1 AND S2 Limiters - Publication Year: 20000101-20211231

396

281,594

S1 TI (((sweeten* or sugary) N2 (drink* or beverage*)) or "nonessential energy-dense" or "non-essential energy-dense" or "sin food*") OR AB (((sweeten* or sugary) N2 (drink* or beverage*)) or "nonessential energy-dense" or "non-essential energy-dense" or "sin food*") OR SU (((sweeten* or sugary) N2 (drink* or beverage*)) or "nonessential energy-dense" or "non-essential energy-dense" or "sin food*" )

4,174

Web of Science (SSCI) – Searched 9th December 2021

6 #2 AND #3

447

5 #4 AND #1

634

4 TS=(tax*)

90,871

3 TS=(subsidies or subsidy or subsidiz* or subsidis* or "tax* incentiv*")

20,580

2 TS=(( (health* NEAR/3 (food* or diet* or eating) ) or fruit* or vegetable* or legum* or pulses or chickpea* or bean or beans or pea or peas or peanut* or soybean* or lentil*))

52,455

1 TS=((((sweeten* or sugary) NEAR/2 (drink* or beverage*) ) or soda or (sin NEAR/2 (drink* or beverage* or food*) ) or ((nonessential or non-essential) N2 (energy-dense or food*) ) ) )

3,785

____________________________________________________________________________

Web of Science (Social Sciences Citation Index) – Draft strategy – Searched 6th December 2021

# 20 37

#19 OR #18 OR #17

# 19 31

#15 AND #14 AND #4 AND #3

# 18 27

#15 AND #13 AND #4 AND #3

# 17 25

#15 AND #12 AND #4 AND #3

# 16 61

#11 AND #4 AND #3

# 15 20,573

TS=(subsidies or subsidy or subsidiz* or subsidis* or "tax* incentiv*")

# 14 36,115

TS=( fruit* or vegetable* or legum* or pulses or chickpea* or bean or beans or pea or peas or peanut* or soybean* or lentil*)

# 13 27,084

TS=( fruit* or vegetable* )

# 12 20,960

TS=( (health* NEAR/3 (food* or diet* or eating) ) )

# 11 633

#10 AND #9

# 10 64,522

TS=(tax*)

# 9 3,779

TS=(((sweeten* or sugary) NEAR/2 (drink* or beverage*) ) or soda or (sin NEAR/2 (drink* or beverage* or food*) ) or ((nonessential or non-essential) N2 (energy-dense or food*) ) )

# 8 2,281

#6 OR #5

# 7 0

AU=Campos-Vasquez*

# 6 2,202

AU=aguilar

# 5 79

AU=taillie

# 4 899,528

TS=(andorra or antigua or "baltic states" or barbuda or aruba or australia or austria or bahamas or bahrain or barbados or belgium or bermuda or "virgin islands" or brunei or canada or "cayman islands" or "channel islands" or chile or croatia or curacao or cyprus or "czech republic" or denmark or Estonia or "faroe islands" or finland or france or "French Polynesia" or Germany or gibraltar or Greece or Greenland or guam or "hong kong" or hungary or Iceland or Ireland or eire or "isle of man" or Israel or Italy or japan or korea or Kuwait or Latvia or Liechtenstein or Lithuania or Luxembourg or macao or macau or malta or Monaco or Nauru or Netherlands or "New Caledonia" or "new zealand" or "northern mariana islands" or Norway or oman or palau or Poland or Portugal or "Puerto rico" or Qatar or "san marino" or "saudi arabia" or Seychelles or Singapore or "sint maarten" or "Slovak republic" or Slovakia or Slovenia or spain or "st kitts and nevis" or "saint martin" or "st martin" or Sweden or Switzerland or Taiwan or "Trinidad and Tobago" or "Turks and caicos" or "united arab emirates" or "Trucial States" or "Abu Dhabi" or Ajman or Dubai or Fujairah or "Ras al-Khaimah" or Sharjah or "Umm al-Qaiwain" or "united kingdom" or uk or Britain or England or Scotland or wales or ulster or "united states" or usa or uruguay)

# 3 695,404

#2 OR #1

# 2 101,901

TS=("systematic review" or "literature review" or "gap map" or "evidence map" or EGM or "scoping review" or "rapid review" or "evidence review")

# 1 617,379

TS=( (random* or experiment* or (match* NEAR/2 (propensity or coarsened or covariate) ) or "propensity score" or "difference in difference*" or "difference-in-difference*" or "differences in difference*" or "differences-in-difference*" or "double difference*" or "quasi-experimental" or "quasi experimental" or "quasi-experiment" or "quasi experiment" or ((estimator or counterfactual) and evaluation*) or "instrumental variable*" or (IV NEAR/2 (estimation or approach) ) or "regression discontinuity" or "time series" or "fixed effects" or counterfactual or "natural experiment*" or "segment* regression" or (non NEAR/2 participant*) or ((control or comparison) NEAR/2 (group* or condition* or area* or intervention) )) )

1. *Unless 90% accuracy of include/exclude decisions is achieved in the training, then single screening with safety first approach would be used (in this approach unclear cases are flagged for review by experienced reviewers).* [↑](#footnote-ref-2)
