# Supplementary material for: Systematic review on fiscal policy interventions in nutrition
Source: Front Nutr. 2022 Nov 29;9:967494. doi: 10.3389/fnut.2022.967494 (PMC9756132; doi:10.3389/fnut.2022.967494)
Supplement: Supplementary file 3 [file Table_3.DOCX]

## Supplement 2: Data extraction tools

### Qualitative data extraction tool

| **Variable group** | **Variable Label** |
| --- | --- |
| **Publication info** | Record type |
|  | Record Title |
|  | Record authors |
|  | Publication year |
|  | URL link |
| **Intervention and implementation considerations** | Intervention |
|  | Intervention details |
|  | Unintended consequences |
|  | Barriers and facilitators to implementation |
| **Evaluation considerations** | Study design |
|  | Covariates |
|  | Outcomes |
| **Sustainability and financial considerations** | Sustainability comments |
|  | Cost effectiveness comments |
| **Other** | Other |
|  | Confidence rating (srr only) |

### Quantitative data extraction tool

| Study ID | Estimate ID | STUDY AND INTERVENTION DESCRIPTIVES | | | | | | | | | |
| --- | --- | --- | --- | --- | --- | --- | --- | --- | --- | --- | --- |
|  |  | Author | Year | Location | Design | How Counterfactual is Chosen? | Analysis type for this effect size | Estimate Type | Comparison | Describe Comparison Group | Subgroup |
|  |  |  |  |  |  |  |  |  |  |  |  |

| STUDY AND INTERVENTION DESCRIPTIVES | | | | | | | | OUTCOMES | | |
| --- | --- | --- | --- | --- | --- | --- | --- | --- | --- | --- |
| If yes to subgroup, describe | Source | Treatment Effect | Intervention description | Intervention Code | Exposure to intervention | Evaluation period | Post-intervention or change from baseline? | Outcome description | Outcome code | Dataset |
|  |  |  |  |  |  |  |  |  |  |  |

| EFFECT SIZE DATA EXTRACTION | | | | | | | | | | | | | | |
| --- | --- | --- | --- | --- | --- | --- | --- | --- | --- | --- | --- | --- | --- | --- |
| Reverse Sign (i.e., decrease is good) | Unit of analysis | mean_t | sd_t | mean_c | sd_c | mean_overall_diff | diff_se | diff _t | diff _p-value | Odds ratio | OR_se | Risk ratio | RR_se | reg_coeff |
|  |  | ***Only applies to RCT's | | | |  |  |  |  |  |  |  |  |  |

| EFFECT SIZE DATA EXTRACTION | | | | | | | | | | | | | |
| --- | --- | --- | --- | --- | --- | --- | --- | --- | --- | --- | --- | --- | --- |
| reg_SE | reg_t | reg_CI_LB | reg_CI_UB | p value | clust_t | clust_c | clust_T | n_t | n_c | n_T | periods (1 if cross sectional) | Does the sample size need to be corrected? | Treatment Variable |
|  |  |  |  |  |  |  |  |  |  |  |  |  |  |

| USED FOR DATA ANALYSIS | | | | | | | | | | | | | | | |
| --- | --- | --- | --- | --- | --- | --- | --- | --- | --- | --- | --- | --- | --- | --- | --- |
| n_T_revised | sp | d | d_rev | g | var(d) | vi | se(d) | CI_l | CI_u | Formula Used | 95ci_lower | 95ci_upper | Checked | ROB Category |  |
|  |  |  |  |  |  |  |  |  |  |  |  |  |  |  |  |
