# Supplementary material for: Systematic review on fiscal policy interventions in nutrition
Source: Front Nutr. 2022 Nov 29;9:967494. doi: 10.3389/fnut.2022.967494 (PMC9756132; doi:10.3389/fnut.2022.967494)
Supplement: Supplementary file 4 [file Table_4.DOCX]

## Supplement 3: Critical appraisal tools

### Appraisal of Risk of Bias for Impact Evaluations using RCT Designs

| General | ID | EPPI ID |  |  |
| --- | --- | --- | --- | --- |
| General | Study first author | Open answer |  |  |
| General | Design type: What type of study design is used? | 1= Randomised controlled trial (RCT) (random assignment to households/individuals) or quasi-RCT  2= Cluster-RCT (quasiRCT) | - |  |
| General | Methods used for analysis: Which methods are used to control for selection bias and confounding? | 1 = Statistical matching (PSM, CEM, covariate matching) 2 = Difference in differences (DID) estimation methods 3 = IV-regression (2stage least squares or bivariate probit) 4 = Heckman selection model  = Fixed effects regression  = Covariate adjusted estimation  = Propensity weighted regression  = Comparison of  means  = Other (please state) | - |  |
| General | Design and analysis method description | Open answer | Briefly describe the study design and analysis method undertaken by the authors. |  |
| General | Study population | Open answer | Provide any details in the paper that describe how the study population was selected, covering:  a) How is the population selected? what is the sampling strategy to recruit participants from that population into the study?  b) What are the characteristics of that study participants?  Was this a pilot programme aimed at being scaled up? d) Were there specific factors of success or failure in the implementation? |  |
| General | Type of comparison group | 1=No intervention  (Service delivery as usual)  2=Other intervention 3=Pipeline (waitlist) control (still service delivery as usual) | Indicate type of comparison group |  |
| General | Type of  comparison group  (If other) | Open answer |  |  |
| General | Ethical clearance | Open answer | Provide any details of ethical research clearances granted. Report unclear if this information is not available. |  |
| General | Study registration | Open answer | Provide any details of study registration, including registry IDs, etc. |  |
| 1: Assignment mechanism - Assessment | Assignment mechanism: Was  the allocation or identification mechanism random or as good as random? | 1= Yes, 2 = Probably  Yes, 3 = Probably No, 4  = No, 8 = Unclear | The authors describe a random component in sequence generation/ randomisation method (e.g.  lottery, coin toss,  random number generator) and assignment is performed for all units at the start of the study centrally or using a method concealed from participants and intervention delivery  If public lottery  is used for the sequence generation, authors provide detail on the exact settings and participants attending the lottery.  If a special  randomization procedure is used to ensure balance, it is well described and justified given the study setting (stratification, pairwise matching, unique random draw, multiple random draws etc).  d) A balance table is reported suggesting that allocation was random between all groups including subgroup receiving different treatment within control or treatment groups (if the comparison is relevant for this assessment). | Score “Yes” if all criterion a), b), c) and d) are satisfied.    Score "Probably Yes" if only criterion a) and b) are not satisfied OR if only criteria c) is not satisfied.    Score “Unclear” if d) is not satisfied because no balance table is reported.    Score "Probably No" if d) is not satisfied because there is no balance table reported and there is evidence suggesting a problem in the randomization, such as baseline coefficients in a diff-in-diff regression table are very different or sample size is too small for the procedure used (using stratification when there are less than two units for each intervention and control group in each strata can lead to imbalance).    Score “No” if d) is not satisfied because there are large imbalances concerning a large number of variables, providing evidence that the assignment was not random. If this is scored as no, use the NRS tool. |
| 1: Assignment mechanism - Justification | Assignment justification | Open answer | Justification for coding decision (Include a brief summary of justification for rating, mentioning your response to all sub questions, cite relevant pages). |  |
| 2: Unit of analysis - Assessment | Unit of analysis: Is unit of analysis in cluster allocation addressed in standard error calculation? | 1=Yes 2=No 3=Not reported/unclear 4=Not applicable | Score "Yes" if UoA = UoR OR if UoA ≠ UoR and standard errors are clustered at the UoR level OR data is collapsed to the UoR level    Score "Not reported/unclear" if  not enough information is provided on the way the standard errors were calculated or what the unit of analysis is.    Score "Not applicable" if it is not a cluster RCT.    Score "No" otherwise. |  |
| 3: Selection bias -  Assessment | Selection bias Was any differential selection into or out of the study (attrition bias) adequately resolved? | 1= Yes, 2 = Probably  Yes, 3 = Probably No, 4  = No, 8 = Unclear | Score "Yes" if there is no attrition or attrition falls into the green zone and the study establishes that attrition is randomly  distributed (e.g. by presenting balance by key characteristics across groups) AND if survey respondents were randomly sampled.    Score "Probably yes" if attrition falls into the green zone AND if survey respondents were randomly sampled.    Score "Unclear" if there is an attrition problem but no information provided on the relationship between attrition and treatment status, OR if there is not enough information on how the population surveyed was sampled.    Score "Probably no" if there is attrition which is likely to be related to the intervention OR there is some indication that the survey respondents were purposely sampled in a way that might have led the sampling to be different between treatment and control groups, or attrition falls into the yellow zone.    Score "No" if attrition falls into the red zone. |  |
| 3: Selection bias -  Justification | Selection bias justification | Open answer | Justification for coding decision (Include a brief summary of  justification for rating, mentioning your response to all sub questions, cite relevant pages). |  |
| 4: Confounding  - Assessment | Confounding and group  equivalence: Was the method of analysis executed adequately to ensure comparability of groups throughout the study and prevent confounding | 1= Yes, 2 = Probably  Yes, 3 = Probably No, 4  = No, 8 = Unclear | Baseline characteristics are similar in magnitude;  Unbalanced covariates at the individual and cluster level are controlled in adjusted analysis; c) Adjustments to the randomization were taken into account in the analysis (stratum fixed effects, pairwise matching variables)? (Bruhn and McKenzie  2009) | Score “Yes” if criterion a) and b) are satisfied;    Score "Probably yes" if a) is not satisfied but b) is satisfied and imbalances are small in magnitude OR if only a) is satisfied.    Score “Unclear” if no balance table is provided or if imbalances are controlled for but they are very large in magnitude and assignment mechanism is not coded as "Yes" or "Probably yes"    Score "Probably no" if a) and b) are not satisfied and the magnitude of imbalances are small    Score “No” if a) and b) are not satisfied and the magnitude of imbalances are large and covariates are clear determinant of the outcomes. |
| 4: Confounding  - Justification | Confounding  justification | Open answer | Justification for coding decision (Include a brief summary of justification for rating, mentioning your response to all sub questions, cite relevant pages). |  |
| 5: Deviations from intended interventions - Assessment | Deviations from intended interventions: Spillovers, crossovers and contamination: was the study adequately protected against spillovers, crossovers and contamination? | 1= Yes, 2 = Probably  Yes, 3 = Probably No, 4  = No, 8 = Unclear | There were no implementation issues that might have led the control participants to receive the treatment (implementer's mistake).  The intervention is unlikely to spillover to comparisons (e.g. participants and non-participants are geographically or socially separated from one another and general equilibrium effects are not likely) or the potential effects of spill overs were measured (e.g. variation in the % of unit within a cluster receiving the treatment).  There is no risk of contamination by external programmes: the treatment and comparisons are isolated from other interventions which might explain changes in outcomes.  d) There is nothing in the surveys that might have given the control participants an idea of what the other group might receive OR they did but there is no risk that this has changed their behaviours; AND the survey process did not reveal information to the control group that they did not have before (e.g. the study aims to measure increase in take up of a service or product that participants might not know about) Authors might put something in place in the design of the study that allows to control for that survey effect (e.g. a pure control with no monitoring except baseline end line) | Score “Yes” if criterion a), b), c) and d) are satisfied;    Score "Probably yes" if there is no obvious problem but there is no information reported on potential risks related to spill overs, contamination, or survey effects in the control group OR if there were issues with spillovers but they were controlled for or measured.    Score “Unclear” if spillovers, crossovers, survey effects or contamination are not addressed clearly.    Score "Probably no" if any of the criterion a), b), c) or d) are not satisfied but the scale of the issue is not clear.    Score “No” if any of the criterion a), b), c) or d) are not satisfied and happened at a large scale in the study. |
| 5: Deviations from intended interventions - Justification | Deviations justification | Open answer | Justification for coding decision (Include a brief  summary of  justification for rating, mentioning your response to all sub questions, cite relevant pages).    For example, intervention groups are geographically separated, authors use intention to treat estimation or instrumental variables to account for non-adherence, and survey questions are not likely to expose individuals in the control group to information about  desirable behaviours (‘survey effects’). |  |
| 6. Performance bias -  Assessment | Performance bias: Was the process of monitoring individuals unlikely to introduce motivation bias among participants? | 1= Yes, 2 = Probably  Yes, 3 = Probably No, 4  = No, 8 = Unclear | The authors state explicitly that the process of monitoring the intervention and outcome measurement is blinded and conducted in the same frequency for treatment and control groups, or argue convincingly why it is not likely  that being monitored could  affect the performance of participants in treatment and comparison groups in different ways (such as resulting in  Hawthorne or John Henry effects).    The outcome is based on data collected in the context of a survey, and not associated with a particular intervention trial, or data are collected from administrative records or in the context of a retrospective (ex post) evaluation. | Score “Yes” if either criterion a) or b) are satisfied;    Score "Probably yes" if the study is based on data collected during a trial and there is no obvious issue with the monitoring processes but authors do not mention potential risks.    Score “Unclear” if it is not clear whether the authors use an appropriate method to prevent Hawthorne and John Henry Effects (e.g. blinding of outcomes and, or enumerators, other methods to ensure consistent monitoring across groups).  Hawthorne effects may result where participants know that they are being observed and John Henry Effects may result from participant knowledge of being compared.    Score "Probably no" if there was imbalance in the frequency of monitoring in intervention groups, which might have influenced participants' behaviours.    Score "No" if neither criterion a) or b) are satisfied. |
| 6. Performance bias -  Justification | Performance bias justification | Open answer | Justification for coding decision (Include a brief summary of justification for rating, mentioning your response to all sub questions, cite relevant pages). |  |
| 7. Outcome measurement  bias -  Assessment | Outcome measurement bias: Was the study free from biases in outcome measurement? | 1= Yes, 2 = Probably  Yes, 3 = Probably No, 4  = No, 8 = Unclear | Outcome assessors are blinded, or the outcome measures are not likely to be biased by their judgement.  For self-reported outcomes:  respondents in the intervention group are not more likely to have accurate answers due to recall bias;  For self-reported outcomes:  respondents do not have incentives to over/under report something related to their performance or actions, OR researchers put in place mechanisms to reduce the risk of reporting bias (researchers not strongly involved in the implementation of the programme and it is clear that their answers to the survey will not affect what they receive in the future) OR authors  have measured the risks of bias through  falsification tests or measuring the effect on placebo outcomes in cases where there was a risk of reporting bias.  d) Timing issue: the data collection period did not differ between intervention and comparison group; the baseline data is not likely to be affected by the beginning of the intervention or affects a small percentage of the study participants. | Score “Yes” if criterion a), b), c) and d) are satisfied:    Score "Probably yes" if there is a small risk related to any of a), b), c) or d) and there is no more information provided to justify the absence of bias OR if there was a high risk of bias but authors have either controlled it in their design or measured  it with a placebo outcomes.    Score “Unclear” if it there is a high risk related to any of a), b), c) or d) and there is no more information provided to justify the absence of bias.  Score "Probably no" if there are high risk related to a), b), c) or d) and it is clear that authors were not able to control for this bias.    Score “No” if there is evidence of bias. |
| 7. Outcome measurement  bias -  Justification | Outcome measurement  justification | Open answer | Justification for coding decision (Include a brief summary of justification for rating, mentioning your response to all sub questions, cite relevant pages). |  |
| 8. Reporting  bias -  Assessment | Analysis reporting: Was the study free from selective analysis reporting? | 1= Yes, 2 = Probably  Yes, 3 = Probably No, 4  = No, 8 = Unclear | A pre-analysis plan or trial protocol is published and referred to or the trial was preregistered, or the outcomes were preregistered;  Authors report results corresponding to the outcomes announced in the method section (there is no outcome reporting bias);  Authors report results of unadjusted analysis and intention to treat (ITT) estimation, alongside any adjusted and treatment-on-the treated/complier average-causal effects analysis.)  Authors use the appropriate analysis method (use baseline data when available) and different treatment arms are  differentiated in the analysis  Authors have reported all the analysis which could help understand the results and no other bias is assessed as unclear due to the  lack of an important analysis (e.g. a balance table or a subgroup analysis) | Score "Yes" if all the criterion a), b), c), d), and e) are satisfied; Score "Probably yes" if all the conditions are met except a), or if all the conditions are met but there is some element missing that could have helped understand the results  better (e);  Score "Unclear" if there is not enough information to determine that there is an analysis missing; Score "Probably no" if any of the criterion b), c) or d) are not satisfied; Score "No" if any of the criterion b), c) or d) are not satisfied and there is evidence that the analysis results would be different because large imbalances were not controlled for, compliance was very low and ITT estimation was not reported or different treatment arms were pooled. |
| 8. Reporting  bias -  Justification | Analysis reporting justification | Open answer | Justification for coding decision (Include a brief summary of justification for rating, mentioning your response to all sub questions, cite relevant pages). |  |
| 9. Other bias -  Assessment | Other risks of bias Is the study free from other sources of bias? | 1= Yes, 4 = No |  |  |
| 9. Other bias -  Justification | Other bias justification | Open answer | Justification for coding decision |  |
| 10. Blinding - observers - Assessment | Blinding of participants? | 1=Yes 2=No 8=unclear  9= N/A | If there is no information, code NO. If there is information but it is ambiguous, code UNCLEAR. |  |
| 10. Blinding - observers - Assessment | Blinding of outcome assessors? | 1=Yes 2=No 8=unclear  9= N/A | If there is no information, code NO. If there is information but it is ambiguous, code UNCLEAR. |  |
| 10. Blinding - analysts - Assessment | Blinding of data analysts? | 1=Yes 2=No 8=unclear  9= N/A | If there is no information, code NO. If there is information but it is ambiguous, code UNCLEAR. |  |
| 10. Blinding - method(s) | Method(s) used to blind | Open answer (including describe method of placebo control) No 9=  N/A | Describe method(s) used to blind |  |
| 11. External validity - Assessment | External validity | Open answer | a) What do authors say about external validity? | Include all information that can help assess the external validity of the results. |

### Appraisal of Risk of Bias for Impact Evaluations using Difference-in-Differences, Instrumental variable design

| Code | Question | Coding | Criteria | Decision-rules |
| --- | --- | --- | --- | --- |
| General | ID | EPPI ID |  |  |
| General | Time taken to complete assessment | Minutes |  |  |
| General | Study first author | Open answer |  | |
| General | Outcomes assessed | Open answer |  |  |
| General | Study design: What type of study design is used? | 1= Natural experiment:  randomised or as-if randomised  2= Natural experiment: regression discontinuity  (RD)  3= CBA (non-randomised  assignment with treatment and contemporaneous comparison group, baseline and end line data collection) – individual repeated measurement 4= CBA pseudo panel (repeated measurement for groups but different individuals)  5= Interrupted time series (with or without contemporaneous control group)  6= Panel data, but no baseline (pre-test)  7 = Comparison group with end line data only |  |  |
| General | Methods used for analysis: Which methods are used to control for selection bias and confounding? | 1 = Statistical matching (PSM, CEM, covariate matching) 2 = Difference in differences (DID) estimation methods 3 = IV-regression (2-stage least squares or bivariate probit)  = Heckman selection model  = Fixed effects regression  = Covariate adjusted estimation  = Propensity weighted regression  = Comparison of means  = Other (please state) | - |  |
| General | Study population | Open answer | Provide any details in the paper that describe how the study population was selected, covering:  How is the population selected? what is the sampling strategy to recruit participants from that population into the study?  What are the characteristics of that study participants?  Was this a pilot programme aimed at being scaled up?  Were there specific factors of success or failure in the implementation? |  |
| General | Ethical clearance | Open answer | Provide any details of ethical research clearances granted. Report unclear if this information is not available. |  |
| 1: Selection bias  - Assessment | 1 - Mechanism of assignment: was the allocation or identification mechanism able to  control for selection bias? | 1= Yes, 2 = Probably Yes,  3 = Probably No, 4 = No,  8 = Unclear |  |  |
| 1: Selection bias  - Justification | For regression discontinuity designs | Open answer | Allocation is made based on a predetermined discontinuity on a continuous variable  (Regression discontinuity design) and blinded to participants or;  if not blinded, individuals reasonably cannot  affect the assignment variable in response to knowledge of the participation decision rule;  and the sample size immediately at both sides of the cutoff point is sufficiently large to equate groups on average. | Score “Yes” if criteria a), b), c) are all satisfied    Score "Probably Yes" if there are minor differences in between both sides of the cut-off point but authors convincingly argue that the differences are unlikely to affect the outcome, OR individuals are not blinded and there are low risk of them affecting the assignment, but the authors do not mention it.    Score “Unclear” if it is unclear whether participants can affect it in response to knowledge of the allocation mechanism.    Score "Probably No" if there are differences between individuals on both sides of the cut-off point, and there are doubts that the differences are due to individuals altering the assignment OR the participants are blinded but there is evidence that the decisions that determined the discontinuity is based on differences between the two groups or differences in time.    Score “No” if the sample size is not sufficient OR there is evidence that participants altered the assignment variable prior to assignment. If the research has serious concerns with the validity of the assignment process or the group equivalence completely fails, we recommend assessing risk of bias of the study using the relevant questions for the appropriate methods of analysis (cross-sectional regressions, difference-in-difference, etc.) rather than the RDDs questions. |
| 1: Selection bias  - Justification | For assignment based nonrandomised programme placement and self-selection (studies using a matching strategy or regression analysis, excluding  IV) | Open answer | Participants and non-participants are either matched based on all relevant characteristics explaining participation and outcomes, or;    all relevant characteristics are accounted for.** and  the data set used contains relevant variables that are measured in a relevant way (i.e.  they were not collected for a different purpose initially and therefore are good proxy for some characteristics).  **Accounting for and matching on all relevant characteristics is usually only feasible  when the  programme  allocation rule is known and there are  no errors of targeting. It is unlikely that studies not based on randomisation or regression discontinuity can score “YES” on this criterion. There are different ways in which covariates can be taken into account. Differences across groups in observable characteristics can be taken into account as covariates in the framework of a regression analysis or can be assessed by testing equality of means between groups. Differences in unobservable characteristics can be taken into account through the use of instrumental variables (see also question 1.d) or proxy variables in the framework of a regression analysis, or using a fixed effects or difference-in-differences model if the only characteristics which are unobserved are time-invariant | Score “Yes” if a) or b) and c) are satisfied    Score "Probably yes" if a) or b) are addressed for but there is some doubt related to c), OR authors combined statistical matching and difference-in-difference to cope with unobservable differences, OR they only did statistical matching and there were clear rules for selection into the program (no self-selection).    Score “Unclear” if · it is not clear whether all relevant characteristics (only relevant time varying characteristics in the case of panel data regressions) are controlled.    Score "Probably no" if only a statistical matching was done and there was self-selection into the program.    Score “No” if relevant characteristics are omitted from the analysis. |
| 1: Selection bias  - Justification | For identification based on an instrumental variable (IV estimation) | Open answer | Score “Yes” if an appropriate instrumental variable is used which is exogenously generated: for example, due to a ‘natural’ experiment  or random allocation.    Score "Probably yes" if there is less evidence (no balance table showing differences between the intervention and comparison group).    Score “Unclear” if the exogeneity of the instrument is unclear (both externally as well as why the variable should not enter by itself in the outcome equation).    Score "Probably no" if there is evidence that enrolment in the program is correlated with a variable that might also have an effect on outcome and on the instrumental variable.    Score “No” if it is clear that the instrument is not exogenous and affect the outcome through other channels than the program. |  |
| 2: Confounding  - Assessment | 2 - Group equivalence: was the method of analysis executed adequately to ensure comparability of groups throughout the study and prevent confounding? | 1= Yes, 2 = Probably Yes,  3 = Probably No, 4 = No, 8 = Unclear |  |  |
| 2: Confounding  - Justification | For regression discontinuity design | Open answer | The interval for selection of treatment and control group is reasonably small OR authors have weighted the matches on their distance to the cutoff point; and  the mean of the covariates of the individuals immediately at both sides of the cut-off point (selected sample of participants and non-participants) are overall not statistically different based on t-test or  ANOVA for equality of means;  Significant differences in covariates of the individuals have been controlled in multivariate analysis; and for cluster-assignment, authors control for external cluster-level factors that might confound the impact of the programme. | Score "Yes, if criterion a), b), c) and d) are addressed.    Score "Probably yes" if b) is not addressed but c) is  addressed and differences in means are not large.    Score “Unclear” if insufficient details are provided on controls; or if insufficient details are provided on cluster controls.    Score "Probably no" if b) is not addressed (absence of a difference test or balance table) and there are doubt regarding the continuity on both sides of the cut-off point (a).    Score “No” otherwise. |
| 2: Confounding  - Justification | For non-randomised trials using difference-in-differences methods of analysis | Open answer | a) The authors use a difference-in-differences (or fixed effects) multivariate estimation method;  b) the authors control for a comprehensive set of individual time varying characteristics, and for cluster assignment, authors control for external cluster-level factors that might confound the impact of the programme**;  c) and the attrition rate is sufficiently low and similar in treatment and control, or the study assesses that dropouts are random draws from the sample (for example, by examining correlation with determinants of outcomes, in both treatment and comparison groups);  **Knowing  allocation rules for the programme – or even whether the non-participants were individuals that refused to participate in the programme, as opposed to individuals that were not given the opportunity to participate in the programme – can help in the assessment of whether the covariates accounted for in the regression capture all the relevant characteristics that explain differences between treatment and comparison groups. | Score "Yes, if a, b, c, d (if relevant) is addressed and baseline imbalances between groups were relatively low OR the method was combined by a statistical matching.    Score "Probably yes" if all possible variables are controlled for and the selection into the program was done according to clear rules, but baseline imbalances between groups were very large.    Score “Unclear” if insufficient details are provided; or if insufficient details are provided on cluster controls.    Score "Probably no" if some time-varying characteristics are not controlled for and the program was self-selected by the intervention groups.    Score “No” if any of the criterion is not addressed. |
| 2: Confounding  - Justification | For statistical matching studies including  propensity scores (PSM) and covariate matching**    **Matching strategies are sometimes complemented with difference-indifference only uses in the estimation the common support region of the sample size, reducing the likelihood of existence of time variant unobservable differences across groups affecting outcome of interest and removing biases arising from time invariant unobservable characteristics. regression estimation methods. This combination approach is superior since it | Open answer | Matching is either on baseline characteristics or time-invariant characteristics which cannot be affected by participation in the programme; and the variables used to match are relevant (for example, demographic and socio-economic factors) to explain both participation and the outcome (so that there can be no evident differences across groups in variables that might explain outcomes); and, for cluster assignment, authors control for external cluster-level factors that might confound the impact of the programme  in addition, for PSM Rosenbaum’s  test suggests the results are not sensitive to the existence of hidden bias; and,  with the exception of Kernel matching, the means of the individual covariates are equated for treatment and comparison groups after matching;  different matching methods including varying sample sizes yields the same results and authors take into account the use of control observations multiple times against the same treatment in their standard error calculation. | Score "Yes, if a, b, c, and d (if relevant) are addressed.    Score "Probably yes" if the selection into the program was done according to clear rules, which are used for the matching but there are slight imbalances remaining after matching.    Score “Unclear” if relevant variables are not included in the matching equation, or if matching is based on characteristics collected at end line; or if insufficient details are provided on cluster controls.    Score "Probably no" if the program was self-selected by the intervention groups or participants OR if the selection into the program was done according to clear rules but there is no baseline data available to match the participants or groups on.    Score “No” if matching was done based on variables that are likely to be affected by the program or any other scenario that affect a), b) c) or d). |
| 2: Confounding  - Justification | For regression-based studies using cross sectional data (excluding IV) | Open answer | a) The study controls for relevant confounders that may be correlated with both participation and explain outcomes (for example, demographic and socio-economic factors at individual and community  level) using multivariate methods with appropriate proxies for unobservable covariates, and, for cluster-assignment, authors control particularly for external cluster-level factors that might confound the impact of the programme;  b) and a Hausman test with an appropriate instrument suggests there is no evidence of endogeneity**;  c) and none of the covariate controls can be affected by participation;  d) and either, only those observations in the region of common support for participants and non-participants in terms of covariates are used, or the distributions of covariates are balanced for the entire sample population across groups;    **The Hausman test explores endogeneity in the framework of regression by comparing whether the OLS and the IV approaches yield significantly different estimations. However, it plays a different role in the different methods of analysis. While in the OLS regression framework the Hausman test mainly explores endogeneity and therefore is related with the validity of the method, in IV approaches it explores whether the author has chosen the best available strategy for addressing causal attribution (since in the absence of endogeneity OLS yields more precise estimators) and therefore is more related with analysis reporting bias. | Score "Yes, if a, b, c and d are addressed.    Score "Probably yes" if all criteria are addressed but authors did not report the Hausman test  (b).    Score “Unclear” if relevant confounders are controlled but appropriate proxy variables or statistical tests are not reported; or if insufficient details are provided on cluster controls.    Score "Probably no" if any of the criterion other than b) is not addressed.    Score “No" if none of the criterion are addressed. |
| 2: Confounding  - Justification | For identification based on an instrumental variable (IV estimation) | Open answer | a) The instrumenting equation is significant at the level of F≥10 (or if an F test is not reported, the authors report and assess whether the R-squared (goodness of fit) of the participation equation is sufficient for appropriate identification); b) the identifying instruments are individually significant (p≤0.01); for Heckman models, the identifiers are reported and significant (p≤0.05);  c) where at least two instruments are used, the authors report on an overidentifying test (p≤0.05 is required to reject the null hypothesis); and none of the covariate controls can be affected by participation and the study, and authors convincingly assesses qualitatively why the instrument only affects the outcome via participation. If the instrument is the random assignment of the treatment, the reviewer should also assess the quality and success of the randomisation procedure in part a).  d) and, for cluster assignment, authors particularly control for external cluster level factors that might confound the impact of the programme (for example, weather, infrastructure, community fixed effects, and so forth) through multivariable analysis. | Score "Yes, if a, b, c, d (if relevant) is addressed.    Score "Probably yes" if one of the tests required for criterion a) or b) is not reported but the other is, and the rest of the criterion are addressed, and the instrument is convincing.    Score “UNCLEAR” if relevant confounders are  controlled for but appropriate statistical tests are not reported; or if insufficient details are provided on cluster controls    Score "Probably no" if exogeneity of the instrument is not convincing and appropriate tests are not reported.    Score “No” otherwise if any of the tests required for criterion a), b) or c) are reported and not satisfied. |
| 3: Performance bias -  Assessment | 3 - Performance bias: was the process of being observed free from motivation bias? | 1= Yes, 2 = Probably Yes,  3 = Probably No, 4 = No,  8 = Unclear | a) For data collected in the context of a particular  intervention trial (randomised or nonrandomised assignment), the authors state explicitly that the process of monitoring the intervention and outcome measurement is blinded, or argue convincingly why it  is not likely that being monitored could affect the performance of participants in treatment and comparison groups in different ways (such as resulting in Hawthorne or John Henry effects).    b) The study is based on data collected in the context of a survey, and not associated with a particular  intervention trial, or data are collected from administrative records or in the context of a retrospective (ex post) evaluation. | Score “Yes” if either criterion a) or b) are satisfied;    Score "Probably yes" if the study is based on survey data collected during a trial and there is no obvious issue with the monitoring processes, but authors do not mention potential risks.    Score “Unclear” if it is not clear whether the authors use an appropriate method to prevent Hawthorne and John Henry Effects (e.g. blinding of outcomes and, or enumerators, other methods to ensure consistent monitoring across groups).  Hawthorne effects may result where participants know that they are being observed and John Henry Effects may result from participant knowledge of being compared.  Score "Probably no" if there was imbalance in the frequency of monitoring in intervention groups, which might have influenced participants' behaviours.    Score "No" if both criterion a) and b) are not satisfied. |
| 3: Performance bias -  Justification | Performance bias -  Justification | Open answer | Justification for coding decision (Include a brief summary of justification for rating, mentioning your response to all sub questions, cite relevant pages). |  |
| 4: Spillovers, crossovers and contamination - Assessment | 4 - Spillovers, crossovers and contamination: was the study adequately protected against spillovers, crossovers and contamination? | 1= Yes, 2 = Probably Yes,  3 = Probably No, 4 = No,  8 = Unclear | There were no implementation issues that might have led the control participants to receive the treatment (implementer's mistake).  The intervention is unlikely to spillover to comparisons (e.g. participants and non-participants are geographically or socially separated from one another and general equilibrium effects are not likely) or the potential effects of spill overs were measured (e.g. variation in the % of unit within a cluster receiving the treatment).  There is no risk of contamination by external programs: the treatment and comparisons are isolated from other interventions which might explain changes in outcomes.  There is nothing in the surveys that might have given the control participants an idea of what the other group might receive OR they did but there is no risk that this has changed their behaviours; AND the survey process did not reveal information to the control group that they did not have before (e.g. the study aims to measure increase in take up of a service or product that participants might not know about) Authors might put something in place in the design of the study that allows to control for that survey effect (e.g. a pure control with no monitoring except baseline end line) | Score “Yes” if criterion a), b), c) and d) are satisfied;    Score "Probably yes" if there is no obvious problem but there is no information reported on potential risks related to spill overs,  contamination, or survey effects in the control group OR if there were issues with spillovers but they were controlled for or measured.  Score “Unclear” if spillovers, crossovers, survey effects or contamination are not addressed clearly.    Score "Probably no" if any of the criterion a), b), c) or d) are not satisfied but the scale of the issue is not clear.    Score “No” if any of the criterion a), b), c) or d) are not satisfied and happened at a large scale in the study. |
| 4: Spillovers, crossovers and contamination - Justification | Spillovers, crossovers and contamination - Justification | Open answer | Justification for coding decision (Include a brief summary of justification for rating, mentioning your response to all sub questions, cite relevant pages). |  |
| 5: Outcome measurement  bias -  Assessment | 5 - Outcome measurement bias | 1= Yes, 2 = Probably Yes,  3 = Probably No, 4 = No,  8 = Unclear | Outcome assessors are blinded or the outcome measures are not likely to be biased by their judgement.  For self-reported outcomes: respondents in the intervention group are not more likely to have accurate answers due to recall bias;  For self-reported outcomes:  respondents do not have incentives to over/under report something related to their performance or actions, OR researchers put in place mechanisms to reduce the risk of reporting bias (researchers not strongly involved in the implementation of the program and it is clear that their answers to the survey will not affect what they receive in the future) OR authors have measured the risks of bias through falsification tests or measuring the effect on placebo outcomes in cases where there was a risk of reporting bias.  Timing issue: the data collection  period did not differ between intervention and comparison group; the baseline data is not likely to be affected by the beginning of the intervention or affects a small percentage of the study participants. | Score “Yes” if criterion a), b), c) and d) are satisfied:    Score "Probably yes" if there is a small risk related to any of a), b), c) or d) and there is no more information provided to justify the absence of bias OR if there was a high risk of bias but authors have either controlled it in their design or measured  it with a placebo outcomes.    Score “Unclear” if it there is a high risk related to any of a), b), c) or d) and  there is no more information provided to justify the absence of bias.    Score "Probably no" if there are high risk related to a), b), c) or d) and it is clear that authors were not able to control for this bias.    Score “No” if there is evidence of bias. |
| 5: Outcome measurement  bias -  Justification | Outcome  measurement bias  - Justification | Open answer | Justification for coding decision (Include a brief  summary of justification for rating, mentioning your response to all sub questions, cite relevant pages). |  |
| 6: Reporting  bias -  Assessment | 6 - Selective analysis reporting: was the study free from selective analysis reporting? | 1= Yes, 2 = Probably Yes,  3 = Probably No, 4 = No,  8 = Unclear | a) a pre-analysis plan is published, especially for prospective NRS but it should also be for retrospective studies b) authors use  ‘common’ methods of estimation (i.e. credible analysis method to deal with attribution given the data available); c) There is no evidence that outcomes were selectively reported (e.g. results for all relevant outcomes in the methods section are reported in the results section) ;  d) Requirements for specific methods of analysis:  - For PSM and covariate matching: (a) Where over 10%  of participants fail to be matched, sensitivity analysis is used to re-estimate results using different matching methods (Kernel Matching techniques); (b) For matching with replacement, no single observation in the control group is matched with a large number of observations in the treatment group. - For IV (including Heckman) models, (a) The authors test and report the results of a Hausman test for exogeneity (p≤0.05 is required to reject the null hypothesis of exogeneity); (b) the coefficient of the selectivity correction term (Rho) is significantly different from zero (P<0.05) (Heckman approach).  - For studies using multivariate regression analysis, authors conduct appropriate specification tests (e.g. testing robustness of results to the inclusion of additional variables, or (very rare) reporting results of multicollinearity test etc). | Score “Yes” if a), b), c) and d) are satisfied OR if a) is not met and it is a retrospective NRS.  Score "Probably Yes" if authors combined methods and reported relevant tests (d) only for one method OR if all the criteria are met except for a) and it is a prospective NRS  Score "Unclear" if intended outcomes not specified in the paper OR if any of the requirements for d) are not reported.  Score "Probably No" if b) is addressed, but authors did not present results for all outcomes announced in the method section OR did not meet requirement d) although reported.  Score “No” if authors use uncommon or less rigorous estimation methods such as failure to conduct multivariate analysis for outcomes equations OR if some important outcomes are subsequently omitted from the results or the significance and magnitude of important outcomes was not assessed. |
| 6: Reporting  bias -  Justification | Analysis reporting bias - Justification | Open answer | Justification for coding decision (Include a brief summary of  justification for rating, mentioning your response to all sub questions, cite relevant pages). |  |
| 7: Other bias -  Assessment | 7 - Other risks of bias: Is the study free from other sources of bias? | 1= Yes, 4 = No | Score “Yes” if the reported results do not suggest any other sources of bias. Score “No” if other potential threats to validity are present, and note these here (e.g. coherence of results, survey instruments used are not reported) |  |
| 7: Other bias -  Justification | Other risks of bias  - Justification | Open answer | Justification for coding decision (Include a brief summary of  justification for rating, mentioning your response to all sub questions, cite relevant pages). |  |
| 8: External validity | 8 - External validity | Open answer | Open answer- what do authors say about external validity, if anything? |  |

### Appraisal of Risk of Bias for Impact Evaluations Using Interrupted Time Series methods

| Code | Question | Coding | Criteria | Decision-rules |
| --- | --- | --- | --- | --- |
| General | ID | EPPI ID |  |  |
| General | Time taken to complete assessment | Minutes |  |  |
| General | Study first author | Open answer |  |  |
| General | Outcome | Open answer |  |  |
| General | Study design: What type of study design is used? | 1= Natural experiment: randomised or as-if randomised  2= Natural experiment: regression discontinuity (RD)  3= CBA (non-randomised assignment with treatment and contemporaneous comparison group, baseline and end line data collection) – individual repeated measurement  4= CBA pseudo panel (repeated measurement for groups but different individuals)  5= Interrupted time series (with or without contemporaneous control group)  6= Panel data, but no baseline (pre-test)  7 = Comparison group with end line data only |  |  |
| General | Methods used for analysis: Which methods are used to control for selection bias and confounding? | 1 = Statistical matching (PSM, CEM, covariate matching) 2 = Difference in differences (DID) estimation methods 3 = IV-regression (2-stage least squares or bivariate probit) 4 = Heckman selection model 5 = Fixed effects or random effects regression 6 = Covariate adjusted estimation 7 = Propensity weighted regression 8 = Comparison of means 9 = Other (please state) | - |  |
| General | Study population | Open answer | Provide any details in the paper that describe how the study population was selected, answering the question: what is the sampling strategy to recruit participants from that population into the evaluation? |  |
| General | Ethical clearance | Open answer | Provide any details of ethical research clearances granted. Report unclear if this information is not available. |  |
| General | Study registration | Open answer | Provide any details of study registration, including registry IDs, etc. |  |
| 1: Confounding | 1.1 Was the identification method free from any sources of bias | 0=Low ROB 1=Some concerns 2=High ROB | The authors use an interrupted time series estimation method and whether the following were addressed: 1. Measurements of outcomes was made for at least three time points per period to permit characterization of pre and post intervention trends and patterns  2. There are compelling arguments that the intervention occurred independently of other changes over time and the outcome was not influenced by other confounding variables/historic events during study period.  3. ARIMA models were used or time series regression models were used to analyse the data and authors have tested/adjusted for potential biases that might be present in the data due to, for instance, autocorrelation, seasonality, or heteroskedasticity | Score 0 Low risk of bias:  If the measurement of outcomes was made at three or more time points per period. AND  there are compelling arguments that the intervention occurred independently of other changes over time and the outcome was not influenced by other confounding variables/historic events during study period AND  authors use either ARIMA models or time series regression models testing/adjusting for potential biases that might be present in the data due to, for instance, autocorrelation, seasonality, or heteroskedasticity  Score 1 Some concerns: The authors have not discussed if the intervention occurred independently of other changes over time  OR Authors have used time series regression models but testing or adjusting for potential biases due to presence of autocorrelation, seasonality or heteroskedasticity have not been reported.   Score 2 High risk of bias: Measurement of outcomes was made at less than three time points per period  OR  the authors do not make a compelling case for the intervention to have occurred independently of other changes over time  OR  authors use only ANOVA or t-tests for the analysis |
| 1: Confounding | Justification for 1.1 |  |  |  |
| 2: Unit of analysis - Assessment | Unit of analysis: Is unit of analysis in cluster allocation addressed in standard error calculation ? | 1=Yes  2=No  3=Not reported/unclear  4=Not applicable | Score "Yes" if Unit of Analysis = Unit of Randomization OR if UoA ≠ UoR and standard errors are clustered at the UoR level OR data is collapsed to the UoR level   Score "Not reported/unclear" if not enough information is provided on the way the standard errors were calculated or what the unit of analysis is.   Score "Not applicable" if it is not a cluster RCT.   Score "No" otherwise. | |
| 2: Unit of analysis - Justification | Question 2 answer justification (provide page numbers) | Open answer |  | |
| 3. Bias due to missing outcome data | 3.1 Did the types of participants/units included in repeated surveys change over time? Was the outcome data missing for whole clusters (unit of multiple individuals) as well as for individual participants? | (0=Low ROB 1=Some concerns 2=High ROB | Did the types of participants/units included in repeatetd surveys change over time? Was the outcome data missing for whole clusters (unit of multiple individuals) as well as for individual participants?   Note - the intervention itself might lead to attrition from the framework or system used to measure outcomes | Score 0 Low risk of bias:  If the types of participants/units included in repeated surveys do not change over time AMD/OR if the outcome data is not missing for whole clusters (unit of multiple individuals) as well as for individual participants OR If missing outcome data were unlikely to bias the results (e.g. the proportion of missing data was similar in the pre- and post-intervention periods)  Score 1 Some concerns: If nothing is reported in the paper on the data being complete across time periods (Do not assume 100% follow up unless stated explicitly)  Score 2 High risk of bias: If the types of participants/units included in repeated surveys change over time OR if the outcome data is missing for whole clusters (unit of multiple individuals) as well as for individual participants and likely to bias the results (e.g. the proportion of missing data was dissimilar in the pre- and post-intervention periods) |
| 3. Bias due to missing outcome data - Justification | Question 3.1 answer justification | Open answer | Justification for coding decision  (Include a brief summary of justification for rating, mentioning your response to all sub questions, cite relevant pages). | |
| 4: Bias due to deviations from intended interventions - Assessment | Whether the effects of any preparatory (pre-interruption) phases of the intervention were accounted for? | (0) Low risk of bias (1) Medium risk of bias/Some concerns (2) High risk of bias | Whether the effects of any preparatory (pre-interruption) phases of the intervention were accounted for?  Note: The interruption time point might be after important feature of the intervention have been implemented. For example, if anticipation of a change in policy or law alters people's behaviour so that there is early impact of the intervention before its main implementation. Such effects will attenuate different between pre and post intervention outcomes. | Score 0 If this issue has been accounted for compellingly or If there is an adequate reason to be believe that this could not be an issue.   Score 1 If there is an adequate reason to believe that this could be an issue and it has not been discussed  Score 2 If there is an adequate reason to believe that this could be an issue and it has not been accounted for compellingly. |
| 4: Bias due to deviations from intended interventions - Justification | Deviations justification | Open answer | Justification for coding decision  (Include a brief summary of justification for rating, mentioning your response to all sub questions, cite relevant pages). | |
| 5. Outcome measurement bias | 5.1 Could the measurement of the outcome be different between the study arms? | (1) Yes; (2) Probably yes; (3) Probably no; (4) No; (5) No information; | 5.1 Were the data collection methods different across time periods? (high rob if they are not, low if they are, and some concerns if there is no info). Similarly, is the group selection similar before and after?  Comparable methods of outcome measurement (data collection) involve the same measurement methods and thresholds, used at comparable time points.   Score 'yes' or 'probably yes' if the data collection methods were different across different time periods   Score 'no' or 'probably no' if it is clearly stated that the sources and methods of data collection are the same both before and after the "interruption"  If no information is provided on the methods of data collection across time periods, then score 'no information' |  |
| 5. Outcome measurement bias - Justification | Question 5.1 answer justification | Open answer | Justification for coding decision  (Include a brief summary of justification for rating, mentioning your response to all sub questions, cite relevant pages). | |
| 5. Outcome measurement bias | 5.2. Were the outcome assessors (enumerators) blinded to the intervention assignment? | (1) Yes; (2) Probably yes; (3) Probably no; (4) No; (5) No information; (6) Not applicable | 5.2. b. Was the primary outcome assessed blindly or measured objectively?  Score ‘yes’ or 'probably yes' if primary outcomes were assessed blindly or measured objectively (e.g., from immunisation card), 'no' or 'probably no' otherwise.   If no information is provided on measurement of the outcomes then score 'no information'.   Note that for participant-reported outcomes, the outcome assessor is the study participant. For participant-reported outcomes, the assessment of outcome is potentially influenced by knowledge or administration of intervention received, leading to a judgement of at least ‘Some concerns’. Review authors will need to judge whether it is likely that participants’ reporting of the outcome was influenced by knowledge or administration of intervention received, in which case risk of bias is considered to be high. |  |
| 5. Outcome measurement bias - Justification | Question 5.2 answer justification | Open answer | Justification for coding decision  (Include a brief summary of justification for rating, mentioning your response to all sub questions, cite relevant pages). | |
| 5. Outcome measurement bias - Assessment | Outcome measurement bias: Was the study free from biases in outcome measurement? | (0) Low risk of bias (1) Medium risk of bias/Some concerns (2) High risk of bias | Utilise your answers for questions 5.1 to 5.3 to determine the overall score using the criteria below | Score 0 Low risk of bias:  If 5.1 is "No" or "Probably no" indicating there is no difference data collection methods across time periods AND the 5.2 is "Yes" or "Probably Yes" indicating primary outcomes were assessed blindly or objectively   Score 1 Some concerns: Scenario 1: If 5.1 is "No" or "Probably no" indicating there is no difference data collection methods across time periods AND 5.2 is "No information" indicating there is no information on the measurement of primary outcomes. . or 5.1 is "No information" on data collection methods across time periods AND 5.2 is ""Yes" or "Probably Yes" indicating primary outcomes were assessed blindly or objectively   Score 2 High risk of bias: If 5.1 is "Yes" or "Probably yes" indicating there is a difference in data collection methods across time periods OR 5.2 is "No" or "Probably no" indicating the assessment of the outcomes is either not blinded or is not objective |
| 5. Outcome measurement bias - Justification | Outcome measurement justification | Open answer | Justification for coding decision  (Include a brief summary of justification for rating, mentioning your response to all sub questions, cite relevant pages). | |
| 6. Reporting bias | 6.1. Is a pre-analysis plan or protocol available which provides sufficient detail? | (1) Yes; (2) Probably yes; (3) Probably no; (4) No; (5) No information; | 6.1. Is a pre-analysis plan or protocol available which provides sufficient detail?  Score 'yes' if they reference a pre-analysis plan, and 'no' otherwise. |  |
| 6. Reporting bias - Justification | Question 6.1 answer justification | Open answer | Justification for coding decision  (Include a brief summary of justification for rating, mentioning your response to all sub questions, cite relevant pages). | |
| 6. Reporting bias | 6.2. Were all primary and secondary outcomes reported as per the pre-analysis plan/protocol or as per the methods description in case of retrospective studies? | (1) Yes; (2) Probably yes; (3) Probably no; (4) No; (5) No information; | 6.2. Were all primary and secondary outcomes reported as per the pre-analysis plan/protocol or as per the methods description in case of retrospective studies?  Score “Yes” or "probably yes" if there is no evidence that outcomes were selectively reported (e.g. results for all relevant outcomes in the methods section are reported in the results section)   Score “No” or "probably no" if some important outcomes are subsequently omitted from the results or the significance and magnitude of important outcomes was not assessed or if multiple measurements of an outcome were made but only one or a subset is reported on the basis of the results (e.g. statistical significance)   Score “No information” if pre-analysis not available or the outcome intentions are not reported in sufficient detail to enable an assessment |  |
| 6. Reporting bias - Justification | Question 6.2 answer justification | Open answer | Justification for coding decision  (Include a brief summary of justification for rating, mentioning your response to all sub questions, cite relevant pages). | |
| 6. Reporting bias | 6.3. Do reported results for the outcomes correspond to all intended analyses? | (1) Yes; (2) Probably yes; (3) Probably no; (4) No; (5) No information; | 6.3. Do reported results for the outcomes correspond to all intended analyses?  A particular outcome domain may be analysed in multiple ways. Examples include: unadjusted and adjusted models; final value vs change from baseline vs analysis of covariance; transformations of variables; different definitions of composite outcomes (e.g. ‘major adverse event’); conversion of continuously scaled outcome to categorical data with different cut-points; different sets of covariates for adjustment; and different strategies for dealing with missing data. Application of multiple methods generates multiple effect estimates for a specific outcome domain. If multiple estimates are generated but only one or a subset is reported on the basis of the results (e.g. statistical significance), there is a high risk of bias in the fully reported result.  Answer ‘No’ or ‘Probably No’ if: There is clear evidence (usually through examination of a trial protocol or statistical analysis plan or methods section in the retrospective studies) that a domain was analysed in multiple ways, but data for only one or a subset of analyses is fully reported (without justification), and the fully reported result is likely to have been selected on the basis of the results. Selection on the basis of the results arises from a desire for findings to be newsworthy, sufficiently noteworthy to merit publication, or to confirm a prior hypothesis. For example, the researchers have done sensitivity analysis for different interruption points, but those who have a preconception or vested interest in showing that an intervention is beneficial may be inclined to selectively report analyses that are favourable to that intervention.   Answer ‘Yes’ or ‘Probably Yes’ if: There is clear evidence (usually through examination of a trial protocol or statistical analysis plan or methods section in the retrospective studies) that all reported results for the outcome domain correspond to all intended analyses, especially the researchers have done sensitivity analysis for different interruption points and reported it appropriately.  or There is only one possible way in which the outcome domain can be analysed (hence there is no opportunity to select from multiple analyses).AC5 Answer ‘No information’ if: Analysis intentions are not available, or the analysis intentions are not reported in sufficient detail to enable an assessment, and there is more than one way in which the outcome domain could have been analysed. |  |
| 6. Reporting bias - Justification | Question 6.3 answer justification | Open answer | Justification for coding decision  (Include a brief summary of justification for rating, mentioning your response to all sub questions, cite relevant pages). | |
| 6. Reporting bias - Assessment | Analysis reporting: Was the study free from selective analysis reporting? | (0) Low risk of bias (1) Medium risk of bias/Some concerns (2) High risk of bias | Utilise your answers for questions 6.1 to 6.3 to determine the overall score using the criteria below | Score 0 Low risk of bias: 6.1 is yes for prospective studies or no for retrospective studies AND 6.2 is "Yes" or "Probably yes", indicating there is no evidence that outcomes were selectively reported AND 6.3 is "Yes" or "Probably yes" indicating all reported results for the outcome domain correspond to all intended analyses.  Score 1 Some concerns:  Scenario 1 - There is no evidence that outcomes were selectively reported (6.2 is "Yes" or "Probably yes") AND there is 'no information' on intended analyses (6.3 is "No information") Scenario 2 - There is 'no information' on outcomes being selectively reported (6.2 is "No information") AND all reported results for the outcome domain correspond to all intended analyses (6.3 is "Yes" or "Probably yes") Scenario 3 - 6.1 is no for prospective studies  Score 2 High risk of bias: If some important outcomes are omitted from the results (6.2 is "No or Probably no") or the significance and magnitude of important outcomes was not assessed OR multiple analyses were made, but only one or a subset is reported on the basis of the results (e.g. statistical significance) (6.3 is "No or Probably no") |
| 6. Reporting bias - Justification | Analysis reporting justification | Open answer | Justification for coding decision  (Include a brief summary of justification for rating, mentioning your response to all sub questions, cite relevant pages). | |
| 7. Other bias - Assessment | Other risks of bias Is the study free from other sources of bias? | 1= Yes,  4 = No |  |  |
| 7. Other bias - Justification | Other bias justification | Open answer | Justification for coding decision  (Include a brief summary of justification for rating, mentioning your response to all sub questions, cite relevant pages). For example, information is collected using a different survey instrument in different intervention groups; measurement of the intervention received in unclear. | |
| 8. Blinding - observers - Assessment | Blinding of participants? | 1 = Yes  2 = No  8 = unclear  9 = N/A | If there is no information, code NO. If there is information but it is ambiguous, code UNCLEAR. | |
| 8. Blinding - analysts - Assessment | Blinding of data analysts? | 1 = Yes  2 = No  8 = unclear  9 = N/A | If there is no information, code NO. If there is information but it is ambiguous, code UNCLEAR. | |
| 8. Blinding - method(s) | Method(s) used to blind | Open answer (including describe method of placebo control) 9 = N/A | Describe method(s) used to blind | |
| 9. External validity - Assessment | External validity | Open answer | a) What do authors say about external validity? Note any additional information related to generalizability. | Include all information that can help assess the external validity of the results. |

### Appraisal of Risk of Bias for Systematic Review methods

| **Assessed by**: |
| --- |
| **Date**: |

**Section A:** *Methods used to identify, include and critically appraise studies*

| **A.1 Were the criteria used for deciding which studies to include in the review reported?**  Did the authors specify:   - Types of studies - Participants/ settings/ population - Intervention(s) - Outcome(s) | - Yes - Partially - No         *Coding guide - check the answers above*  *YES: All four should be yes*  *NO: All four should be no*  *PARTIALLY: Any other* |
| --- | --- |
| *Comments (note important limitations or uncertainty)* | |
| **A.2 Was the search for evidence reasonably comprehensive?**  Were the following done:   - Language bias avoided (no restriction of inclusion based on language) - No restriction of inclusion based on publication status - Relevant databases searched (Minimum criteria: All reviews should search at least one source of grey literature such as Google; for health: Medline/ PubMed + Cochrane Library; for social sciences IDEAS + at least one database of general social science literature and one subject specific database) - Reference lists in included articles checked - Authors/experts contacted | - Yes - Partially - No - Can’t tell                 *Coding guide - check the answers above:*  *YES: All five should be yes*  *PARTIALLY: Relevant databases and reference lists are both reported*  *NO: Any other* |
| *Comments (note important limitations or uncertainty)* | |
| **A.3 Does the review cover an appropriate time period?**  *Is the search period comprehensive enough that relevant literature is unlikely to be omitted?* | - Yes - Can't tell (only use if no information about time period for search) - No    Unsure      *Coding guide:*  *YES: Generally this means searching the literature at least back to 1990*  *NO: Generally if the search does not go back to 1990 CAN’T TELL: No information about time period for search*    *Note: With reference to the above – there may be important reasons for adopting different dates for the search, e.g. depending on the intervention. If you think there are limitations with the timeframe adopted for the search which have not been noted and justified by the authors, you should code this item as a NO and specify your reason for doing so in the comment box below. Older reviews should not be downgraded, but the fact that the search was conducted some time ago should be noted in the quality assessment. Always report the time period for the search in the comment box.* |
| *Comments (note search period, any justification provided for the search period, or uncertainty)* | |

| **A.4 Was bias in the selection of articles avoided?**  Did the authors specify:   - Independent screening of full text by at least 2 reviewers - List of included studies provided - List of excluded studies provided | - Yes - Partially - No       *Coding guide:*  *YES: All three should be yes, although reviews published in journals are unlikely to have a list of excluded studies (due to limits on word count) and the review should not be penalised for this. PARTIALLY: Independent screening and list of included studies provided are both reported*  *NO: All other. If list of included studies provided, but the authors do not report whether or not the screening has been done by 2 reviewers review is downgraded to NO.* |
| --- | --- |
| *Comments (note important limitations or uncertainty):* |  |
| **A.5 Did the authors use appropriate criteria to assess the quality and risk of bias in analysing the studies that are included? ^ii^**   - The criteria used for assessing the quality/ risk of bias were reported - A table or summary of the assessment of each included study for each criterion was reported - Sensible criteria were used that focus on the quality/ risk of bias (and not other qualities of the studies, such as precision or applicability/external validity).   “Sensible” is defined as a recognised quality appraisal tool/ checklist, or similar tool which assesses bias in included studies. Please see footnotes for details of the main types of bias such a tool should assess. | - Yes - Partially - No       *Coding guide:*  *YES: All three should be yes*  *PARTIALLY: The first and third criteria should be reported. If the authors report the criteria for assessing risk of bias and report a summary of this assessment for each criterion, but the criteria may be only partially sensible (e.g. do not address all possible risks of bias, but do address some), we downgrade to PARTIALLY.*  *NO: Any other* |
| *Comments (note important limitations or uncertainty)* |  |

**Section B:** *Methods used to analyse the findings*

| **B.1 Were the characteristics and results of the included studies reliably reported?**  Was there:   - Independent data extraction by at least 2 reviewers  A table or summary of the characteristics of the participants, interventions and outcomes for the included studies - A table or summary of the results of all the included studies | - Yes - No - Partially - Not applicable (e.g. no included studies)     *Coding guide:*  *YES: All three should be yes*  *PARTIALLY: Criteria one and three are yes, but some information is lacking on second criteria.*  *No: None of these are reported. If the review does not report whether data was independently extracted by 2 reviewers (possibly a reporting error), we downgrade to NO.*  *NOT APPLICABLE: if no studies/no data* |
| --- | --- |
| *Comments (note important limitations or uncertainty)* |  |
| **B.2 Are the methods used by the review authors to analyse the findings of the included studies clear, including methods for calculating effect sizes if applicable?** | - Yes - Partially - No - Not applicable (e.g. no studies or no data)     *Coding guide:*  *YES: Methods used clearly reported. If it is clear that the authors use narrative synthesis, they don't need to say this explicitly.*  *PARTIALLY: Some reporting on methods but lack of clarity*  *NO: Nothing reported on methods*  *NOT APPLICABLE: if no studies/no data* |
| *Comments (note important limitations or uncertainty)* |  |
| **B.3 Did the review describe the extent of heterogeneity?**   - Did the review ensure that included studies were similar enough that it made sense to combine them, sensibly divide the included studies into homogeneous groups, or sensibly conclude that it did not make sense to combine or group the included studies? - Did the review discuss the extent to which there were important differences in the results of the included studies? - If a meta-analysis was done, was the I^2^, chi square test for heterogeneity or other appropriate statistic reported? If no statistical test was reported, is a qualitative justification made for the use of random effects? | - Yes - Partially - No - Not applicable (e.g. no studies or no data)         *Coding guide:*  *YES: First two should be yes, and third category should be yes if applicable should be yes*  *PARTIALLY: The first category is yes*  *NO: Any other*  *NOT APPLICABLE: if no studies/no data* |
| *Comments (note important limitations or uncertainty)* |  |

| **B.4 Were the findings of the relevant studies combined (or not combined) appropriately relative to the primary question the review addresses and the available data?**    How was the data analysis done?   - Descriptive only - Vote counting based on direction of effect - Vote counting based on statistical significance - Description of range of effect sizes - Meta-analysis - Meta-regression - Other: specify - Not applicable (e.g. no studies or no data)   How were the studies weighted in the analysis?   - Equal weights (this is what is done when vote counting is used) - By quality or study design (this is rarely done)  Inverse variance (this is what is typically done in a meta-analysis) - Number of participants (sample size) - Other: specify - Not clear - Not applicable (e.g. no studies or no data)   Did the review address unit of analysis errors?   - Yes - took clustering into account in the analysis (e.g.   used intra-cluster correlation coefficient)   - No, but acknowledged problem of unit of analysis errors - No mention of issue - Not applicable - no clustered trials or studies included | - Yes - Partially - No - Not applicable (e.g. no studies or no data) - Can’t tell     *Coding guide:*  *YES: If appropriate table, graph or metanalysis AND appropriate weights AND unit*  *of analysis errors addressed (if appropriate).*  *PARTIALLY: If appropriate table, graph or meta-analysis AND appropriate weights AND unit of analysis errors not addressed (and should have been).*  *NO: If narrative OR vote counting (where quantitative analyses would have been possible) OR inappropriate reporting of table, graph or meta-analyses.*  *NOT APPLICABLE: if no studies/no data CAN’T TELL: if unsure (note reasons in*  *comments below)* |
| --- | --- |

| *Comments (note important limitations or uncertainty)* | |
| --- | --- |
| **B. 5 Does the review report evidence appropriately?**     - The review makes clear which evidence is subject to low risk   of bias in assessing causality (attribution of outcomes to intervention), and which is likely to be biased, and does so appropriately   - Where studies of differing risk of bias are included, results are reported and analysed separately by risk of bias status | - Yes - No - Partially - Not applicable     *Coding guide:*  *YES: Both criteria should be fulfilled (where applicable)*  *NO: Criteria not fulfilled*  *PARTIALLY: Only one criteria fulfilled, or when there is limited reporting of quality appraisal (the latter applies only when inclusion criteria for study design are appropriate)*  *NOT APPLICABLE: No included studies*    *Note on reporting evidence and risk of bias: For reviews of effects of ‘large n’ interventions, experimental and quasi-experimental designs should be included (if available). For reviews of effects of ‘small n’ interventions, designs appropriate to attribute changes to the intervention should be included (e.g. pre-post with assessment of confounders)* |
| *Please specify included study designs and any other comments (note important limitations or uncertainty):* | |
| **B.6 Did the review examine the extent to which specific factors might explain differences in the results of the included studies?**   - Were factors that the review authors considered as likely explanatory factors clearly described? - Was a sensible method used to explore the extent to which key factors explained heterogeneity? - Descriptive/textual - Graphical - Meta-analysis by sub-groups - Meta-regression - Other | - Yes - Partially - No - Not applicable     *Coding guide:*  *YES: Explanatory factors clearly described and appropriate methods used to explore heterogeneity*  *PARTIALLY: Explanatory factors described but for meta-analyses, sub-group analysis or meta-regression not reported (when they should have been)*  *NO: No description or analysis of likely explanatory factors*  *NOT APPLICABLE: e.g. too few studies, no important differences in the results of the included studies, or the included studies were so dissimilar that it would not make sense to explore heterogeneity of the results* |
| *Comments (note important limitations or uncertainty)* | |

Section C: Overall assessment of the reliability of the review

| **C.1 Are there any other aspects of the review not mentioned before which lead you to question the results?** | - Additional methodological concerns – only one person reviewing  Robustness - Interpretation - Conflicts of interest (of the review authors or for included studies) - Other - No other quality issues identified |
| --- | --- |
| **C.2 Are there any mitigating factors which should be taken into account in determining the reviews reliability?** | - Limitations acknowledged - No strong policy conclusions drawn (including in abstract/ summary)  Any other factors |
| *Use comments to specify if relevant, to flag uncertainty or need for discussion* | |
| C.3 **Based on the above assessments of the methods please provide a summary of the quality of the review**      Strengths and limitations should be summarised above, based on what was noted in Sections A, B and C. | |

NOTES

1. Adapted from Supporting the Use of Research Evidence (SURE) Collaboration. SURE checklist for making

judgements about how much confidence to place in a systematic review. In: SURE guides for preparing and using policy briefs. [www.evipnet.org/sure](http://www.evipnet.org/sure)

1. **Risk of bias** is the extent to which bias may be responsible for the findings of a study.

**Bias** is a systematic error or deviation from the truth in results or inferences. In studies of the effects of social, economic and health care interventions, the main types of bias arise from systematic differences in the groups that are compared (selection bias), the intervention that is provided, or exposure to other factors apart from the intervention of interest (performance bias/contamination), withdrawals or exclusions of people entered into a study (attrition bias) or how outcomes are assessed (detection bias) and reported (reporting bias). Reviews of social science studies may be particularly affected by reporting bias, where a biased subset of all the relevant data and analyses is presented.

Assessments of the risk of bias are sometimes also referred to as assessments of the **validity** or **quality** of a study.

**Validity** is the extent to which a result (of a measurement or study) is likely to be true.

**Quality** is a vague notion of the strength or validity of a study, often indicating the extent of control over bias.
