# Supplementary material for: Systematic review on fiscal policy interventions in nutrition
Source: Front Nutr. 2022 Nov 29;9:967494. doi: 10.3389/fnut.2022.967494 (PMC9756132; doi:10.3389/fnut.2022.967494)
Supplement: Supplementary file 5 [file Table_5.DOCX]

## Supplement 4: Additional risk of bias results

We found high risk of bias for all but one difference-in-difference study. The most common risk was confounding, where authors did not include relevant time-varying characteristics or external cluster-level factors that might confound the impact of the programme (Supplement 4, Figure 1; Supplement 4, Figure 2). Most interrupted time series studies (n = 9) were rated as having some concerns related to confounding and reporting bias, where authors did not report all outcomes for all analysed data (Supplement 4, Figure 3; Supplement 4, Figure 4). We assessed the sole randomised control as high-risk of bias due to concerns related the assignment mechanism, selection bias, confounding and reporting (Supplement 4, Figure 5). We assessed the instrumental variable study as having some concerns of bias associated with selection bias, confounding and reporting bias (Supplement 4, Figure 6). We did not assess the synthetic control studies for risk of bias because the method is new and quality standards are not yet established.

Both systematic reviews were rated with high confidence. There were minimal concerns with the causal chain used in the review to analyse studies and the type of evidence incorporated to inform the analysis and reporting (Supplement 4, Figure 7).

### Study quality and risk of bias of difference-in-difference studies

Even though most of the difference-in-difference studies reported some sort of parallel trend verification, the most common issue we observed was associated with confounding (Supplement 4, Figure 1). Authors did not include relevant time-varying characteristics and/or external cluster level factors that might confound the impact of the programme in their specifications. We identified deviations from the intended intervention as another source of concern given that most studies did not account for potential contamination confounding impacts of other programs or information campaigns associated with the tax policy, nor did they consider cross-border purchases of taxed products in comparison sites.

Supplement 4, figure 1: Frequency of bias in DiD studies


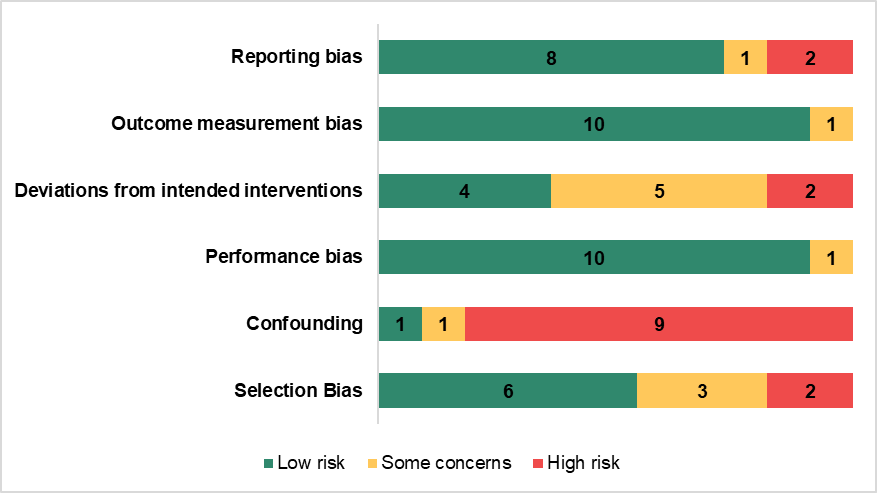


Supplement 4, Figure 2: Risk ob Bias of included DiD studies

### Study quality and risk of bias of interrupted time series studies

We rated most interrupted time series studies as having some concerns of bias related to confounding and reporting bias (Supplement 4, Figure 3). For confounding, there was limited discussion of independence of interventions from other factors, such as historic events occurring during the evaluation period, which may have confounded the impact of the policies. The only study accounting for this was Alvarado and colleagues (2019) who controlled for purchases of vinegar to account for a change in VAT-exempt products as a potential time-varying confounder. Some taxes were implemented as part of larger fiscal reforms with the purpose of increasing revenues for the government. Others were implemented following the advice of the WHO to prevent obesity and were usually accompanied by information campaigns on the adverse health effects of consuming taxed high-sugar foods and beverages. For reporting bias, authors often reported primary outcomes related to purchases, but did not use all pre-specified data to measure secondary outcomes. For instance, authors sometimes use body-mass index (BMI) for subgroup analysis, but do not report the effects of taxes on BMI.

Supplement 4, Figure 3: Frequency of bias in ITS studies


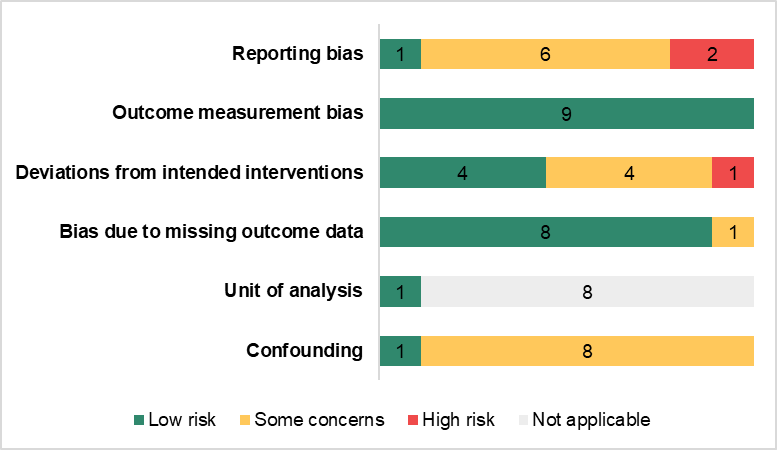


Supplement 4, Figure 4: Risk ob Bias of included ITS studies

* Assessment of the unit of analysis was not applicable for most studies because either all observations were exposed to treatment after the interruption point or there were only two clusters.

### Study quality and risk of bias of RCT study

In the only included RCT (Øvrum and Bere 2013), the assignment mechanism was not clearly described (Supplement 4, Figure 4). Even if schools were randomly assigned to the programme, the students who chose to subscribe were not. The authors did not report a baseline table testing for the balance of observed characteristics or any other evidence to rule out problems in the randomisation. They also did not account for this self-selection when relying on multivariate analysis. Further, ex-post survey data is used for the analysis where social desirability bias might influence the self-reported consumption of fruits and vegetables, the main outcome of interest.

Supplement 4, Figure 5: Risk ob Bias of included RCTs

### Study quality and risk of bias of instrumental variable study

Concerns from the instrumental variable study come from the lack of a clear justification for the selection of instruments and their exogeneity and limited reporting of tests for the validity of the instruments (Howard and Prakash 2011; Supplement 4, Figure 6).

Supplement 4, Figure 6: Risk ob Bias of included IV studies

### Study quality and risk of bias of Systematic reviews

Both included systematic reviews (SRs) were ranked ‘high-confidence’ (Supplement 4, Figure 7). Both reviews reported comprehensive search methods that included grey literature and relevant data bases, contacted authors and experts, and checked reference lists.

Authors also used rigorous methods to include and critically appraise studies, including independent screening and data extraction. Pfinder and colleagues (2020) could not perform meta-analysis because only one study was included and therefore used narrative methods to analyse the outcomes of interest. Teng and colleagues (2019) performed meta-analysis comparing similar enough studies by outcomes and measure. They also report an extensive sub-group analysis and explored the extent to which specific factors might explain differences in the results of included studies.

Supplement 4, Figure 7: Systematic Review Critical Appraisal criteria

| **Review Criterion** | **Teng et al. 2019** | **Pfinder et al. 2020*** |
| --- | --- | --- |
| Methods used to identify, include and critically appraise studies | High confidence | High confidence |
| Methods used to analyse the findings relative to the primary question addressed in the review | High confidence | High confidence |
| **Overall reliability of the review** | **High confidence** | **High confidence** |

**Note.* Pfinder and colleagues (2020) only includes one evaluation on the Hungary tax, which was evaluated in one analysed study (Kurz and König 2021).
