# Supplementary material for: Systematic review on fiscal policy interventions in nutrition
Source: Front Nutr. 2022 Nov 29;9:967494. doi: 10.3389/fnut.2022.967494 (PMC9756132; doi:10.3389/fnut.2022.967494)

## Supplemental Material 5: Additional meta-analysis results

**Detailed results for the impacts of taxes on purchasing of taxed and untaxed beverages**

We included $k=5$ studies in the analysis. The estimated average outcome based on the random-effects model was $\hat{\mu}=-0.07$ (95% CI: $-0.25$ to $0.11$). Therefore, the average outcome did not differ significantly from zero ($z=-0.80$, $p=0.42$). According to the $Q$-test, the true outcomes appear to be heterogeneous ($Q\left( 4 \right)=22.17$, $p<0.01$, $\hat{\tau}^{2}=0.02$, $I^{2}=81.96$%). We examined the studentized residuals and found that two studies (Aguilar et al. 2019; Nakamura et al. 2018) had values larger than $\pm2.58$ and may be potential outliers in the context of this model. According to the Cook’s distances, none of the studies could be considered overly influential.

**Detailed results for the impacts of taxes on purchasing of taxed beverages**

A total of $k=15$ studies were included in the analysis. The observed outcomes ranged from $-2.51$ to $0.91$. The estimated average outcome based on the random-effects model was $\hat{\mu}=-0.18$ (95% CI: $-0.29$ to $-0.07$). Therefore, the average outcome differed significantly from zero ($z=-3.23$, $p<0.01$). According to the $Q$-test, the true outcomes appear to be heterogeneous ($Q\left( 14 \right)=335.19$, $p<0.01$, $\hat{\tau}^{2}=0.03$, $I^{2}=95.82$%).

We examined the studentized residuals revealed that one study (Powell et al. 2021) had a value larger than $\pm2.94$ and may be a potential outlier in the context of this model. According to the Cook’s distances, one study (Powell et al. 2021) could be overly influential.

A funnel plot of the estimates is shown in Figure 5. Neither the rank correlation nor the regression test indicated any funnel plot asymmetry ($p=0.38$ and $p=0.29$, respectively).

**Figure 1: Funnel plot on purchasing of taxed beverages**


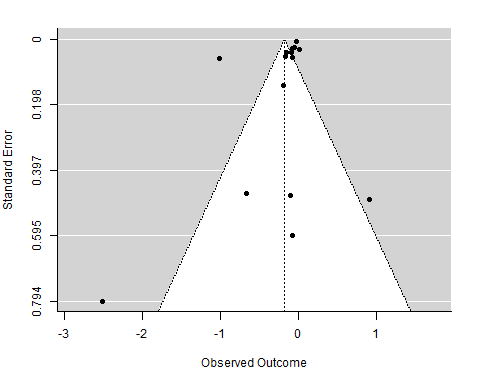


**Detailed results for the impacts of taxes on purchasing of untaxed beverages**

We included $k=11$ studies in the analysis. The estimated average outcome based on the random-effects model was $\hat{\mu}=-0.02$ (95% CI: $-0.06$ to $0.02$). Therefore, the average outcome did not differ significantly from zero ($z=-0.98$, $p=0.33$). According to the $Q$-test, the true outcomes appear to be heterogeneous ($Q\left( 10 \right)=19.61$, $p=0.03$, $\hat{\tau}^{2}=0.00$, $I^{2}=49.01$%).

We examined the studentized residuals revealed that none of the studies had a value larger than $\pm2.84$ and hence there was no indication of outliers in the context of this model. According to the Cook’s distances, none of the studies could be considered to be overly influential. A funnel plot of the estimates is shown in Figure 6. Neither the rank correlation nor the regression test indicated any funnel plot asymmetry ($p=0.45$ and $p=0.08$, respectively).

**Figure 2: Funnel plot on purchases of untaxed beverages**


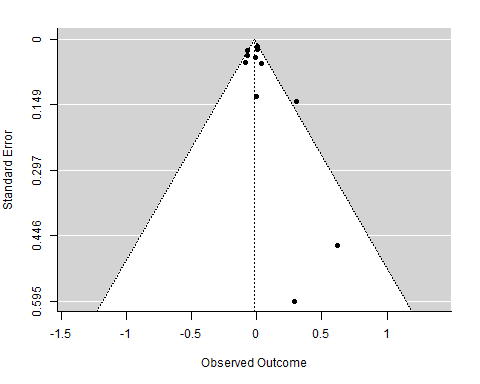


**Detailed results for the impacts of subsidies on diet quality**

We included $k=3$ studies in the analysis. The observed outcomes ranged from $0.01$ to $0.22$. The estimated average outcome based on the random-effects model was $\hat{\mu}=0.06$ (95% CI: $-0.01$ to $0.14$). Therefore, the average outcome did not differ significantly from zero ($z=1.62$, $p=0.10$). According to the $Q$-test, the true outcomes appear to be heterogeneous ($Q\left( 2 \right)=12.79$, $p<0.01$, $\hat{\tau}^{2}=0.00$, $I^{2}=84.37$%). An examination of the studentized residuals revealed that one study (Øvrum and Bere 2013) had a value larger than $\pm2.39$ and may be a potential outlier in the context of this model. According to the Cook’s distances, none of the studies could be considered to be overly influential.

**Figure 3: The evidence is too limited to draw conclusions about effects of taxes on SSBs on the purchasing of sugary foods**
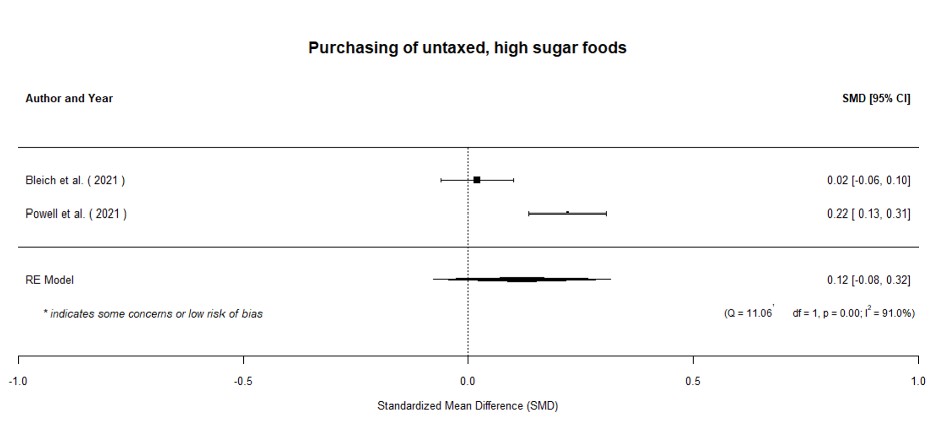


**Figure 4: The evidence base is too limited to draw conclusion about effects of taxes on SSBs have on evaluated measures of diet quality**


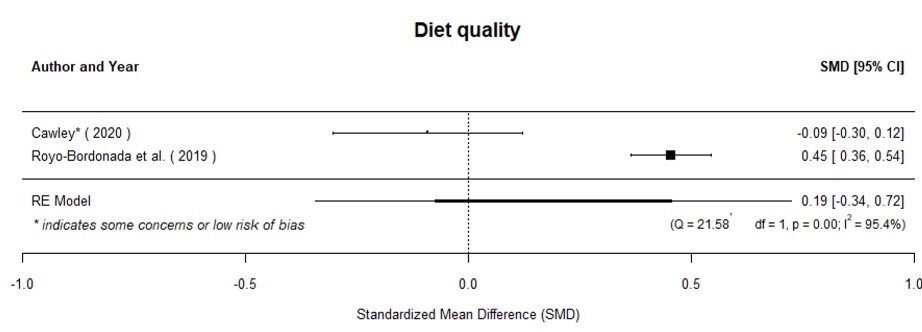


**Figure 5: There is limited evidence on the effects of SSB and high-sugar food taxes on purchases of sugary foods**


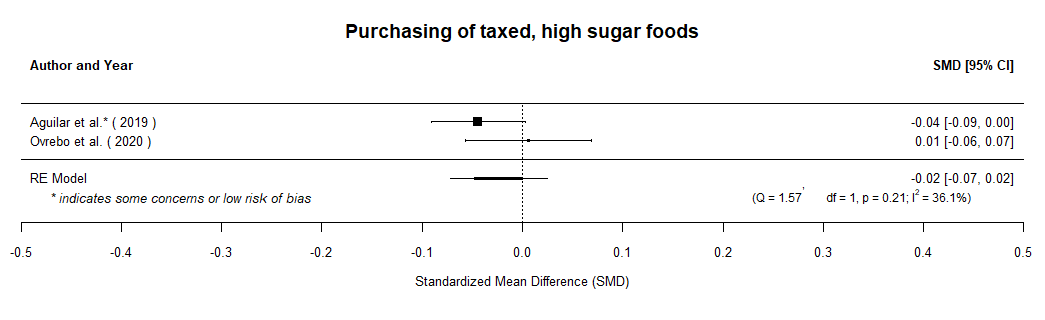


**Figure 6: There is limited evidence on the effect of subsidies**

Panel A


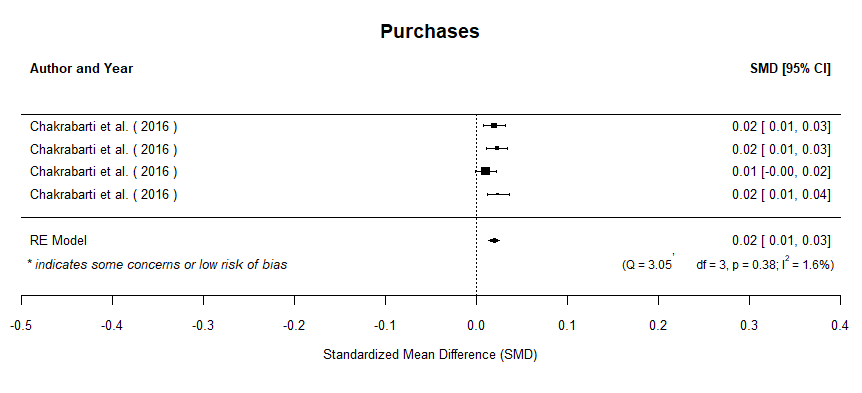


Panel B


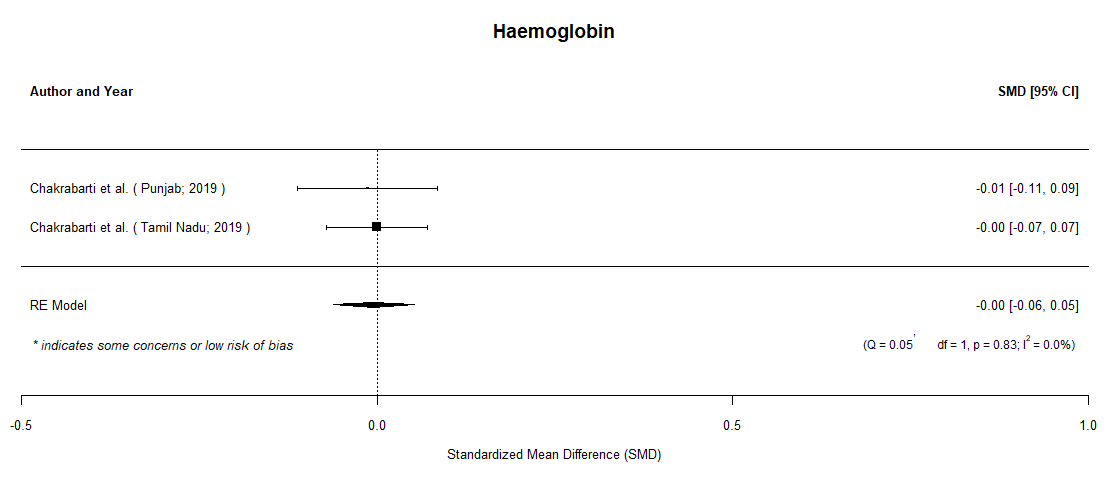


Panel C


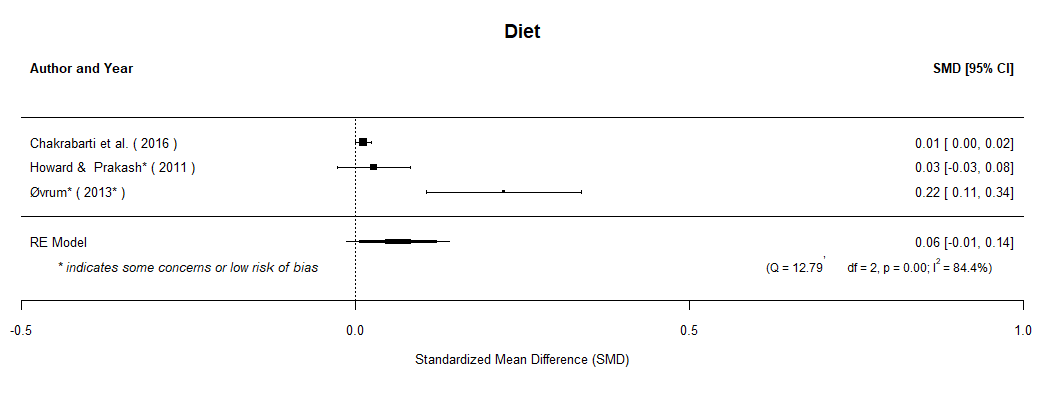

Supplement: Supplementary file 6 [file Table_6.docx]
